# SWIM: a computational tool to unveiling crucial nodes in complex biological networks

**DOI:** 10.1038/srep44797

**Published:** 2017-03-20

**Authors:** Paola Paci, Teresa Colombo, Giulia Fiscon, Aymone Gurtner, Giulio Pavesi, Lorenzo Farina

**Affiliations:** 1 Institute for Systems Analysis and Computer Science “Antonio Ruberti”, National Research Council, Rome, Italy; 2 SysBio Centre for Systems Biology, Rome, 00185, Italy; 3 Department of Research, Advanced Diagnostics, and Technological Innovation, Translational Research Area, Regina Elena National Cancer Institute, Rome, Italy; 4 Department of Biosciences, University of Milan, Italy; 5 Department of Computer, Control and Management Engineering, “Sapienza” University, Rome, Italy

## Abstract

SWItchMiner (SWIM) is a wizard-like software implementation of a procedure, previously described, able to extract information contained in complex networks. Specifically, SWIM allows unearthing the existence of a new class of hubs, called “fight-club hubs”, characterized by a marked negative correlation with their first nearest neighbors. Among them, a special subset of genes, called “switch genes”, appears to be characterized by an unusual pattern of intra- and inter-module connections that confers them a crucial topological role, interestingly mirrored by the evidence of their clinic-biological relevance. Here, we applied SWIM to a large panel of cancer datasets from The Cancer Genome Atlas, in order to highlight switch genes that could be critically associated with the drastic changes in the physiological state of cells or tissues induced by the cancer development. We discovered that switch genes are found in all cancers we studied and they encompass protein coding genes and non-coding RNAs, recovering many known key cancer players but also many new potential biomarkers not yet characterized in cancer context. Furthermore, SWIM is amenable to detect switch genes in different organisms and cell conditions, with the potential to uncover important players in biologically relevant scenarios, including but not limited to human cancer.

Real-world networks (such as technological, social, and biological networks) are virtually always organized in cohesive groups of nodes (communities, modules, clusters) that often correspond to distinct functional units^1^,^2^,^3^,^4^. This confers a sort of modular organization to these networks where the graph ‘modularity’ can be used to quantify the extent to which nodes are ‘close’ to each others. The concept of proximity is measured by a distance metric (*i.e.* weights of the edges) used by the myriad of existing algorithms for detecting communities in networks^2^,^3^,^5^. The community structure of real-word networks is one of the non-trivial topological features (including also a heavy-tailed degree distribution, a high clustering coefficient, and assortativity or disassortativity among nodes) that do not occur in simple networks such as random graphs, but are characteristic of complex networks, whose study was indeed inspired by the empirical study of real-world networks.

One of the key problems in complex networks analysis is to classify nodes in the network as a whole. Usually, this problem is solved by using different centrality measurements (degree, closeness, betweenness, eigenvector centrality, etc …). An alternative approach is the categorization of hubs according to the date/party dichotomy, defined in ref. 6 for protein-protein interaction (PPI) networks in yeast, that assigns roles to hubs (*i.e.* nodes with degree at least equal to 5, where degree refers to the number of links outgoing from a node) purely on the basis of gene expression data rather than on the basis of network topology. The authors in ref. 6 examined the extent to which hubs are co-expressed with their linked nodes (interaction partners) in the yeast interactome. By computing the averaged Pearson correlation coefficient (APCC) of expression over all interaction partners of each hub, they concluded that hubs fall into two distinct categories: date hubs that display low co-expression with their partners (low APCC) and party hubs that have high co-expression (high APCC). It was proposed that date and party hubs play different roles in the modular organization of the network: party hubs are thought to coordinate single functions performed by a group of proteins (*i.e.* nodes in the PPI network) that are all expressed at the same time (*i.e.* party hubs are local coordinators), whereas date hubs are described as higher-level connectors between groups that perform varying functions and are active at different times or under different conditions (*i.e.* date hubs are global connectors).

By computationally partitioning metabolic networks into functionally coherent subnetworks, the authors in refs 7 and 8 show that the roles of nodes could be more diverse than allowed by a binary classification and could be related to the group structure of the network. In particular, nodes are classified into a small number of system-independent ‘universal roles’ based on the connectivity of each node both within its own community and to other communities. This enables a coarse-grained, and thus simplified, description of the network that the authors in refs 7 and 8 called ‘cartographic representation’ of complex networks. This role assignment is based on the general idea that nodes with the same role should have similar topological properties. In ref. 5 the authors examined the extent to which these structural roles match up with the date/party hypothesis, finding little evidence to support it.

Inspired by the Guimerà-Amaral approach^7^,^8^ and by the node-based date/party categorization, we have recently proposed^9^ a new approach to the problem of nodes classification in the context of the modular organization of gene expression networks. By combining topological role classification and gene expression data, our methodology paves the way for a reconciliation of the date/party hypothesis with the topology. Most importantly, our methodology provides a fast and systematic way to identify a small pool of key regulatory genes, that we called *switch genes*, which are likely to be critically associated with drastic changes in many biological settings. We set our approach by studying the transition between mature to immature phase of the developmental program of grapevine (*Vitis Vinifera*)^9^ and found that switch genes are master regulators of the previously reported transcriptome remodeling^9^ that marks the developmental shift from immature to mature growth^10^.

Here, taking advantage from the vast human cancer data available from The Cancer Genome Atlas^11^, we present an application of the algorithm to the identification of switch genes in several human cancers along with its wizard-like Graphical User Interphase (GUI) implementation called SWIM (for Switch Miner).

## Results

### Identification of fight-club hubs in human cancer gene correlation networks

The date/party distinction was originally motivated by an approximately bimodal distribution of hub co-expression (*i.e.* APCC) in PPI networks. Recently, we applied this hub classification to study the topological properties of genes, as opposed to proteins, in the gene expression network of *Vitis Vinifera*^9^. A detailed description on how to build a gene expression network using the Pearson correlation, in general and not limited to the case of grapevine, is provided in the User Guide (Supplementary File 1, step 3 of the SWIM algorithm), a practical document that describes how SWIM work and to use it. Shortly, in a gene expression network nodes are RNA transcripts and an edge occurs between two nodes if their expression profiles are highly correlated or anti-correlated (*i.e.* if the absolute value of the Pearson correlation coefficient between the expression profiles of two nodes exceeds a given cutoff).

Differently to PPI networks, the hub classification based on the APCC in the gene expression network of *Vitis Vinifera* strikingly led to a trimodal distribution of the hub APCC values^9^, with two peaks in correspondence to low and high positive APCC values that resemble date and party hubs of the PPI networks, respectively. However, we also discovered a new third peak, which is unmatched in PPI networks and represents negative APCC values. We called nodes populating this peak *fight-club hubs*^9^.

Here, we perform the same analysis as for *Vitis Vinifera* by exploiting the large panel of TCGA cancer datasets^11^. Strikingly, we recovered an approximately trimodal distribution of the hub APCC values (see Supplementary Fig. 1) in each gene expression network built for each single TCGA cancer dataset, which enabled us to identify fight-club hubs also in human cancers.

In order to detect communities in our correlation networks, we scored node proximity based on the following distance metric:


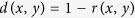


where *r(x, y*) is the Pearson correlation coefficient between the expression profiles of two linked nodes *x, y*. Thus, two nodes are close (belonging to the same community) if they are moderately to highly correlated (*d* → 0), while they are far apart (lying in different communities) if they are poorly correlated or moderately to highly anti-correlated (*d* → 2).

Accordingly, most inter-community interactions would connect to fight-club hubs or, at most, date hubs. On the contrary, most intra-community interactions would connect to party hubs. In fact, inter-community interactions occur between any two nodes that, belonging to different communities, display a negative or poorly positive correlation (*i.e.* high values of *d*). Thus, the corresponding APCC will necessarily range from negative to poorly positive values. Along the same line, since intra-community interactions occur between highly-correlated nodes (*i.e.* low values of *d*), the APCC will be necessarily confined to highly positive values.

This interesting relation observed between the hub classification and the network community structure is in perfect accord with the topological roles that date and party hubs should fulfill in the model of organized modularity proposed in ref. 6: date hubs are supposed to connect different biological processes performed by ‘player’ acting at different place and time, while party hubs are supposed to interact with their partners simultaneously.

Completely different situation concerns fight-club hubs that, having no analogue in the PPI networks, constitute a new, potentially fascinating, territory yet to be explored. Likewise date hubs, they are supposed to connect different biological processes but differently from them, fight-club hubs display an opposite transcriptional pattern with respect to their interaction partners: if they are induced their interaction partners are repressed, and viceversa. Although it is not possible to infer a causal relationship by time-independent gene expression data, this scenario is compatible with a function of negative regulation of at least a sub-set of fight-club hubs on their partners. Indeed, it is know that the human genome is pervasively transcribed^12^, yet at any given spatio/temporal state a cell generally uses only a fraction of its gene functions. Thus, suggesting a crucial role of negative regulation to save cells from activation of specific pathways and cell functions in response to specific external stimuli or physiological and/or pathological changes. As an example, microRNAs (miRNAs) are now universally recognized as key negative regulators in many intracellular processes as well as in cancer development and progression^13^,^14^,^15^,^16^,^17^.

### Identification of switch genes in human cancer gene correlation networks

The authors in refs 7 and 8 defined two parameters: the participation coefficient *P* that measures how much a node ‘communicates’ with any module (including its own) and the within-module degree *z* that measures how ‘well-connected’ a node is to other nodes in its module. In this sense, *P* represents a global measure of connectivity, whereas *z* is essentially a local measurement of connectivity. The formal definitions of *P* and *z* for a generic node *i* are the following^7^,^8^:


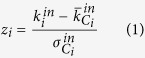



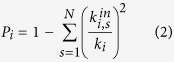


where *C*_*i*_ is the own cluster of node *i*; 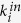 is the number of links of node *i* to other nodes in *C*_*i*_ (internal degree); 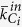 and 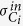 are, respectively, the average and the standard deviation of the internal degree distribution of the all nodes in *C*_*i*_; 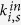 is the number of links of node *i* to other nodes in the generic cluster *s* (including its own that is *s* = *C*_*i*_ and 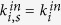); *k*_*i*_ is the total degree of node *i*. The sum extends to all *N* modules of the network.

The higher the number of clusters with which a node is connected (including its own), the greater the value of *P* (see Supplementary Fig. 2). Thus, this approach assigns a high value of the participation coefficient *P* to those nodes that have links uniformly distributed among all modules (including their own), leading to an upper limit for *P* that is susceptible to changes in the number of clusters used to partition the network:


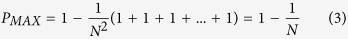


In light of this, the region R4 of the Guimerà-Amaral cartography, populated by the ‘non-hub kinless nodes’ and corresponding to high values of the participation coefficient (*P* > 0.8), results completely emptied as long as the number of clusters is small, *i.e.* for *N* < 6 (see Supplementary Fig. 3). As a consequence, structural roles appear to be assigned to nodes depending on the number of clusters used by the network partitioning instead of relying on their functional relevance in the network.

By emphasizing the difference between the intra-module and inter-module connections, we revisited in ref. 9 the node roles definition given in refs 7 and 8. Specifically, we defined in ref. 9 a new statistic, the *clusterphobic coefficient K*_*π*_, which measures the ‘fear’ of each node of being confined in a cluster, in analogy with the claustrophobic disorder:


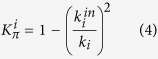


where 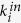 and *k*_*i*_ are the internal and the total degree of node *i*, respectively. *K*_*π*_ measures the ratio of internal to external connections and assumes its minimum values (*K*_*π*_ = 0) when a node has only links within its own module, *i.e.* it does not ‘communicates’ to other modules (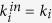). On the contrary, *K*_*π*_ approaches to its maximum (*K*_*π*_ → 1) when a node interacts mainly outside its own community (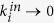).

Similarly to the participation coefficient *P*, the clusterphobic coefficient *K*_*π*_ represents a measure of global connectivity. However, our definition of *K*_*π*_ envisages an upper limit (*K*_*π*_ = 1) that is essentially topological in nature and is thus identifiable from the network rather than depending on network partitioning.

Comparing the two definitions of the participant coefficient, we obtain:


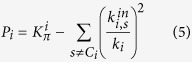


where the first term (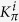) represents the contribution to *P*_*i*_ coming from the interactions of the node *i* within its own community with respect to its total interactions, while the second term (the sum) represents the contribution due to the interactions of the node *i* with all the other communities with respect to its total interactions. Thus, *P* is always lower than *K*_*π*_ and is getting closer to *K*_*π*_ the more the links of the node *i* deviate from being uniformly distributed among all modules, but rather displaying a preferential direction of interactions towards the cluster to which the node *i* belongs. Indeed, they approach the same lower bound (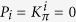) when the node *i* has only links within its own module.

We also generalized in ref. 9 the within-module degree definition of Guimerà-Amaral^7^,^8^ by defining a global within-module degree *z*_*g*_ that measures how a node is ‘well-connected’ to other nodes in its own module with respect to their global connectivity:


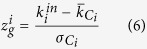


where 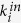 is the number of links of node *i* to nodes in its module *C*_*i*_; 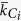 and 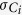 are the average and standard deviation of the total degree distribution of the nodes in the module *C*_*i*_. Differently from the clusterphobic coefficient *K*_*π*_, the global within-module degree *z*_*g*_ represents a measure of local connectivity.

The values of the two statistics, the global within-module degree and the clusterphobic coefficient, divide the plane (*K*_*π*_, *z*_*g*_) into seven regions encompassing seven possible roles. Similarly to^7^,^8^, this yields to a cartographic representation of a gene expression network where we added a further characterisation of the nodes by coloring them according to the their expression APCC, obtaining what we called *heat cartography map*^9^ (see Supplementary Fig. 4).

Of note, our categorization of date, party, and fight-club hubs, indicates that a correlation between expression APCC and structural roles can be supported (see Supplementary Fig. 5). In fact, according to their definitions, party hubs function mainly inside modules and, consistently, we found their maximum density in the regions R1 and R2 of the heat cartography that are populated by ‘peripheral nodes’ and characterized by low values of the clusterphobic coefficient. Date hubs, interacting with different modules at different time and place, reasonably reach their highest density in the region R3 that is populated by ‘non-hub connectors’ and characterized by moderate values of the clusterphobic coefficient. Finally, fight-club hubs, acting as negative regulators, mainly fall in the region R4 that is characterized by high values of the clusterphobic coefficient and by a strong inclination of nodes to ‘leave’ their own community (*i.e.* these nodes interact mostly outside their own community). We called this subset of fight-club hubs lying in the region R4 *switch genes*^9^ that are thus characterized by the following topological features:

1. To mainly interact outside their own community (high values of *K*_*π*_).
2. To be not local hubs (low values of *z*_*g*_).
3. To be mainly anti-correlated with their interaction partners (negative APCC).

Our nodes classification differs from the one proposed in refs 7 and 8 mainly with respect to the definition of the clusterphobic coefficient *vs*. the participation coefficient (see equation 5) that in turn determines which nodes populate the region R4. Importantly, from this derives the possibility or not to identify switch genes, whose main feature is that they mainly interact outside their community regardless of the number of clusters toward which these interactions are targeted. In principle, it is sufficient that a node interacts with at least one cluster in addition to its own, as long as the number of connections to this cluster are greater than the internal ones, to be classified as switch gene. In this case, the participation coefficient would be very small whereas the clusterphobic coefficient would be very high. As a consequence, following the Guimerà-Amaral approach, switch genes would have never been identified or, even worse, they would have been classified as ‘peripheral nodes’ instead of as important connectors of the gene expression network (see Supplementary Fig. 6). In particular, according to our node roles definition, switch genes appear to convey information between different function-specific modules by acting as important negative regulators of their interaction partners: if they are activated their interactors are switched off, if they are inactivated their interactors are switched on. In this sense, they can also be referred to as *bottlenecks*.

In support of this hypothesis, our recent study on grapevin^9^ found that switch genes - mainly involved in secondary metabolism (color, aroma and flavor of the fruit) - are over-expressed in mature organs and highly anti-correlated with thousands of genes that are down-regulated in mature organs and mainly involved in primary metabolisms that are related to vegetative growth (such as photosynthesis and cell proliferation). This evidence suggests that the transition to mature growth predominantly involves the suppression of vegetative pathways rather than the activation of maturation-specific pathways. This strongly supports the importance of a negative regulation both at transcriptional and post-transcriptional level.

### Topological relevance of fight-club hubs and switch genes in human cancer gene correlation networks

We systematically explored the topological relevance of fight-club hubs and switch genes in the gene expression networks built from the large panel of TCGA cancer datasets^11^. All these human cancer correlation networks show a power law behavior of the degree distribution probability function indicating that most genes interact with few partners, whereas a small but significant proportion of genes, the hubs, interact with many partners (see Supplementary Fig. 7). It’s well known that networks with a degree distribution following a power law are amazingly resistant to random node removal but are extremely sensitive to the targeted removal of hubs that causes the destruction of the network integrity, an effect known as ‘vulnerability to attack’^6^,^18^. This property offers a way to test the relevance of nodes with respect to the overall network connectivity, which can be measured by the average shortest path - where the shortest path between two nodes is the minimum number of edges connecting them and the average shortest path is the mean of the shortest paths for all possible pairs of nodes in the network.

We investigated whether fight-club, date, and party hubs have distinct topological properties by evaluating the effects produced on the human cancer correlation networks upon their deletion (see Supplementary Fig. 8). Removing date hubs led to a very rapid increase of the average shortest path presumably indicating a very rapid disintegration of the network into multiple components. On the contrary, removal of party hubs exerted much less effect on global connectivity, whereas the random removal of nodes does not effect the integrity of the network letting almost unchanged the average shortest path. Thus, the different effects on the overall topology resulting from the removal of date and party hubs appear to mirror their well-known distinct topological properties in the PPI network and supports the hypothesis of date hubs as higher-level connectors between groups. Strikingly, the removal of fight-club hubs produces a drastic increase of the average shortest path that is in most cases very similar to the effect caused by the deletion of date hubs. Evaluating the contribute of switch genes to the robustness (*i.e.* resilience to errors) of the network, we found that the drastic effect observed on the deletion of fight-club hubs, was largely due to their subset of switch genes rather than being a generic property of the entire set of fight-club hubs (see Supplementary Fig. 8).

Hence, despite not being amongst the top ranked hubs (*i.e.* about 30 hubs with degree greater than the 98^*th*^ percentile of the degree distribution), switch genes appear to be critical nodes in preserving the integrity of the human cancer co-expression networks.

### Clinical relevance of switch genes in human breast invasive carcinoma

**Figure 1:**
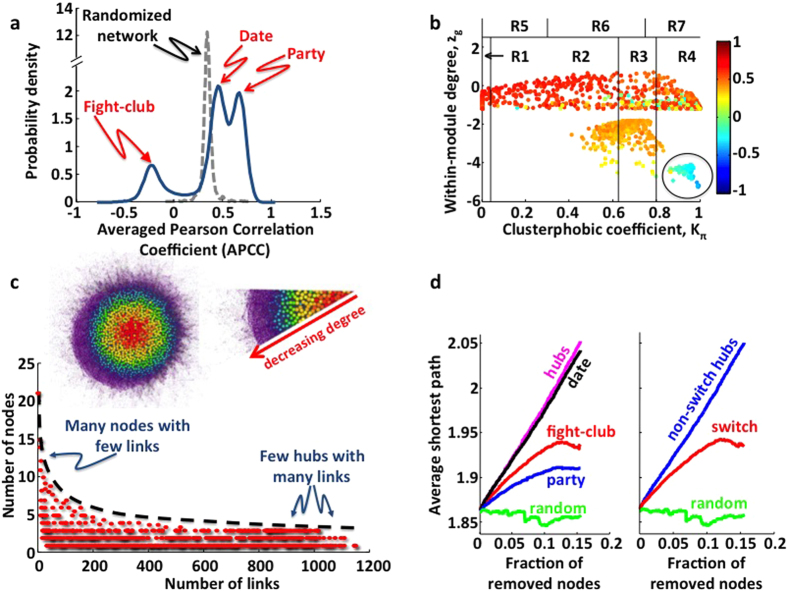
Integrated network analysis identified switch genes as putative key regulators of breast cancer transformation. (**a**) Probability distribution of APCC for hubs (*i.e.* nodes with degree greater than 5 according^6^) identified in the correlation network built from the breast cancer expression dataset (blue solid line) and in its randomized counterpart obtained by shuffling the edges but preserving the degree of each node (grey dashed line). Differently from the randomized case, the true APCC distribution shows a clear trimodal pattern where peaks correspond to previously reported hub categories (such as party and date hubs) but also to the new category of hubs, the “fight-club hubs”. (**b**) Heat cartography map of nodes in the breast cancer correlation network. Dots in the plot, that correspond to nodes in the correlation network, are distributed across seven regions (R1 to R7) according to their clusterphobic coefficient *K*_*π*_ (x-axis), that is a measure of the propensity of a node to communicate with nodes in different modules, and to their within-module degree of communication *z*_*g*_ (y-axis). Dot color renders the APCC value in a red (positive APCC) to blue (negative APCC) scale. The black circle marks switch genes. (**c**) Node degree distribution in the breast cancer correlation network. The frequency distribution plot shows a clear power law behavior with most of the nodes marked by a small number of links in the network and a minority of nodes (hubs) marked by high links number. The same pattern is mirrored by coloring nodes based to decreasing degree on a red to purple scale (inset above the frequency distribution plot). (**d**) Analysis of network robustness. Scatter plots of average shortest path (y-axis) as a function of the fraction of removed nodes (x-axis) for targeted removal of different categories of hub nodes and for random node removal (see line labels in the plots). In the left panel, hubs are stratified according to the APCC-based classification of party, date, and fight-club hubs, while in the right panel hub nodes are stratified based on their topological characteristics in “switch” and “non-switch” hubs.

Breast cancer is the second most common cancer worldwide after lung cancer, the fifth most common cause of cancer death, and the leading cause of cancer death in women. The global burden of breast cancer alone exceeds all other cancers and its incidence rates are increasing. In light of these frightening statistics and motived by the relevance of identifying new potential ‘high risk’ breast cancer susceptibility genes, we first focused on the analysis of the results obtained by applying SWIM on the TCGA dataset of breast invasive carcinoma (see Fig. 1).

Our algorithm identified 257 switch genes, out of 1710 differentially expressed genes (*i.e.* 15%), including protein coding genes as well as long non-coding RNAs and miRNAs (see Supplementary Table 1). Of note, among switch genes we found many well-known cancer-related mRNAs (such as BIRC5, AURKA, RAD51, MBL and EZH2)^19^,^20^,^21^,^22^,^23^ as well as oncogenic miRNAs (such as the miR-200 family, miR-21, miR-203, miR-210, the miR-182/183/96 cluster)^24^,^25^,^26^,^27^,^28^. This supports the reliability of the selection made by our analysis and opens new challenges in determining the role played in breast cancer by the switch genes that are not yet characterized in this context (see Supplementary Table 1).

**Figure 2:**
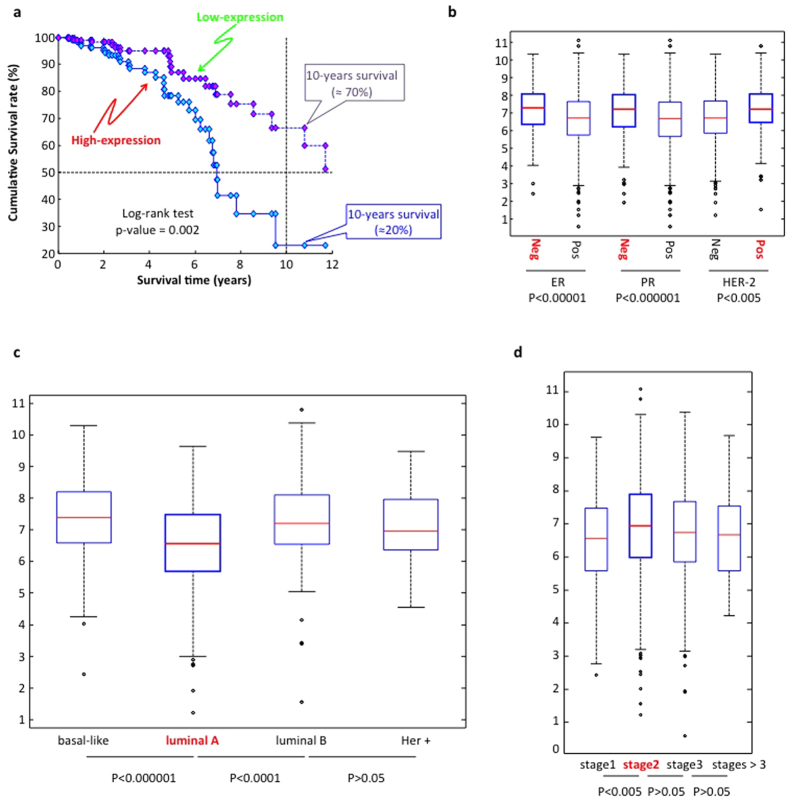
BRIP1, a breast cancer switch gene, has prognostic value for patient survival and correlates with disease aggressiveness. (**a**) Kaplan-Meier analyzes for the correlations between BRIP1 expression and 10-year survival in breast invasive carcinoma patients. Low- and high-expression groups refer to patients with expression levels lower than the 25^*th*^ and greater than the 75^*th*^ percentile, respectively. (**b–d**) Box plots illustrating comparison of BRIP1 median expression levels across breast cancer patients grouped by hormone receptor status (panel b), molecular subtypes (panel c) and tumor staging (panel d). Red labels identify significantly different comparison groups based on p-value < 0.05 (Wilcoxon sum rank test). Abbreviations: Neg: negative receptor status (*i.e.* cancer cells that do not have a protein to which the hormone will bind and do not need hormone to grow, and usually do not stop growing when treated with hormones); Pos: positive receptor status; ER: estrogen receptor; PR: progesterone receptor; HER2: human epidermal growth factor receptor 2; Her+: ER neg, PR neg and HER-2 Pos.

Seeking to probe the clinical relevance of these cancer-deregulated genes, we first investigated their prognostic value by performing a Kaplan-Meier survival analysis for each of them. Patients were stratified in two cohorts based on the given switch gene expression levels (low or high) and a log-rank test was performed to assess the p-value (where the lower the p-value, the better the separation between the two prognosis groups). Finally, the switch genes were sorted by increasing p-values in order to identify the best at separating the two prognosis groups (see Supplementary Table 2). Importantly, we found that switch genes generally have a great prognostic value in predicting the overall survival. Among the top ten, we found BRCA1-interacting protein C-terminal helicase 1 (BRIP1), whose low-expression level was associated with improved overall survival in breast invasive carcinoma patients (see Fig. 2a). In addition, comparative analysis of the median expression levels of BRIP1 between different patient subgroups identified based on hormone receptor status (*i.e.* estrogen receptor ER, progesterone receptor PR, human epidermal growth factor receptor 2 HER-2), molecular subtypes (*i.e.* basal-like, luminal A, luminal B, Her+) or histological characteristics (*i.e.* tumor stages) showed that its expression was positively correlated with an unfavorable outcome (see Fig. 2b–d). In particular, increased BRIP1 transcript levels were found in tumors with an estrogen receptor-negative, progesterone receptor-negative or HER-2-positive status, in basal-like cancers and in stage 2 tumors as compared to stage 1 carcinomas. Together these results indicate that the overexpression of BRIP1 can contribute to breast cancer malignancy.

The clinical relevance of this gene is further supported by the evidence that mutations in BRIP1 confer an increased risk of breast cancer and transcription of BRIP1 has been found to be cell growth-related and controlled by the E2F/retinoblastoma (Rb) pathway through a conserved E2F-responsive site^29^.

Interestingly, among switch genes that have a significant prognostic value in breast cancer we found not only well-characterized protein coding genes (like BRIP1) and the miR-210 whose correlation with breast cancer mortality has been previously reported^30^, but also a validated pseudogene (LOC148709 or actin gamma 1 pseudogene) not characterized yet.

### TCGA multi-cancer analysis of switch genes

The integrated analysis performed on each cancer-specific correlation network built from the large panel of TCGA cancer datasets^11^ consistently identified cancer-related switch genes that appear to be biologically relevant, as testified by statistically significant enrichment in functional annotation to cancer associated cell processes and gene regulatory networks, such as cell cycle control, development and surveillance of DNA integrity (see Supplementary Table 3). Notably, we found the well-known oncogenic long non-coding RNA PVT1^31^,^32^, the oncogenic miR-182/183/96 cluster, and well-known oncogenic mRNAs (such as BIRC5, MYBL2, FOXM1, PLK1)^33^,^34^,^35^,^36^ as the most common switch genes to the majority of tumors (see Supplementary Table 1, Supplementary Fig. 9 and Supplementary Fig. 10).

**Figure 3:**
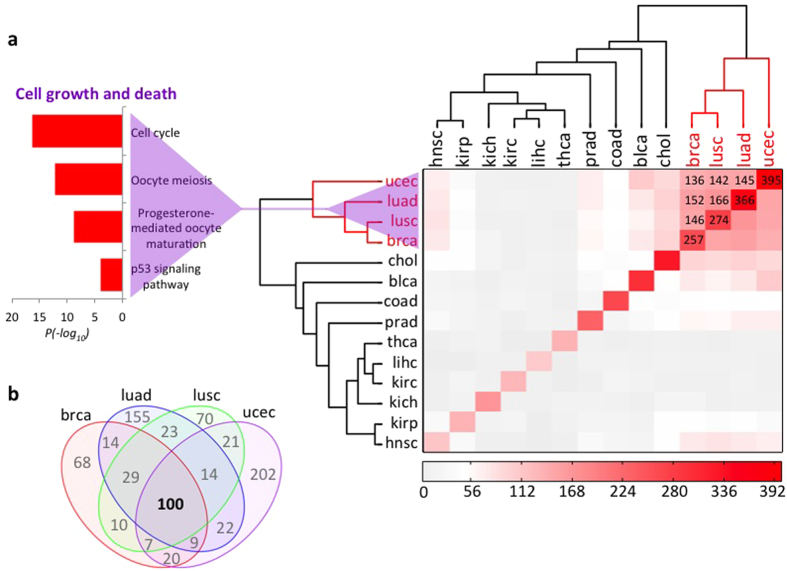
Multi-cancer identification of switch genes. (**a**) Comparative analysis of switch genes in a panel of 14 common cancer types whose expression data was available from TCGA. [Right] The distance matrix computed based on Hamming distance of binary-encoded (1 = present; 0 = absent) annotation of switch genes across cancers is rendered in a symmetrical heat map where decreasing distance is rendered in a white to red scale. Dendrograms on columns and rows of the distance matrix indicate cancer clustering based on Hamming distance. [Left] KEGG pathways (www.genome.jp/kegg/) enrichment analysis for the set of switch genes shared by the well-separated cluster of four cancers (brca, lusc, luad, ucec) highlighted in red in the dendrograms. (**b**) Venn diagram detailing the counts of cancer-specific or common switch genes among the four grouped cancers (brca, lusc, luad, ucec). Abbreviations. blad: bladder urothelial carcinoma; brca: breast invasive carcinoma; chol: cholangiocarcinoma; coad: colon adenocarcinoma; hnsc: head and neck squamous cell carcinoma; kich: kidney chromophobe; kirc: kidney renal clear cell carcinoma; kirp: kidney renal papillary cell carcinoma; lihc: liver hepatocellular carcinoma; luad: lung adenocarcinoma; lusc: lung squamous cell carcinoma; prad: prostate adenocarcinoma; thca: thyroid carcinoma; ucec: uterine corpus endometrial carcinoma.

To compare sets of switch genes between the different TCGA cancer datasets analyzed, we translated results of the multi-cancer integrated network analysis in a binary matrix by assigning 1 if the given gene (matrix row) was found as a switch for the given cancer dataset (matrix column), and 0 otherwise. We then used the Hamming distance to group cancer types based on their degree of switch genes similarity (see Fig. 3a - Right). With the result of this analysis it became possible to point out a cluster of four cancer datasets (*i.e.* breast invasive carcinoma, lung adenocarcinoma, lung squamous cell carcinoma, and uterine corpus endometrial carcinoma) sharing a substantial number of switch genes (N = 100, see Fig. 3b and Supplementary Table 4), whose biological pathways annotation enrichment analysis revealed significant association to relevant pathways in cancer, generally involved in regulation of cell growth and death (see Fig. 3a - Left). Evidence of switch genes common to different cancer types is a key finding suggesting recurrent patterns of deregulation of cancer gene regulatory networks at the expense of the same regulatory nodes. It is worth to stress that the number of the cancer-recurrent switch genes among the four grouped cancers is not affected by the choice of the correlation thresholds used to build the correlation networks as long as the integrity of the network is preserved (see Supplementary Fig. 11).

**Figure 4:**
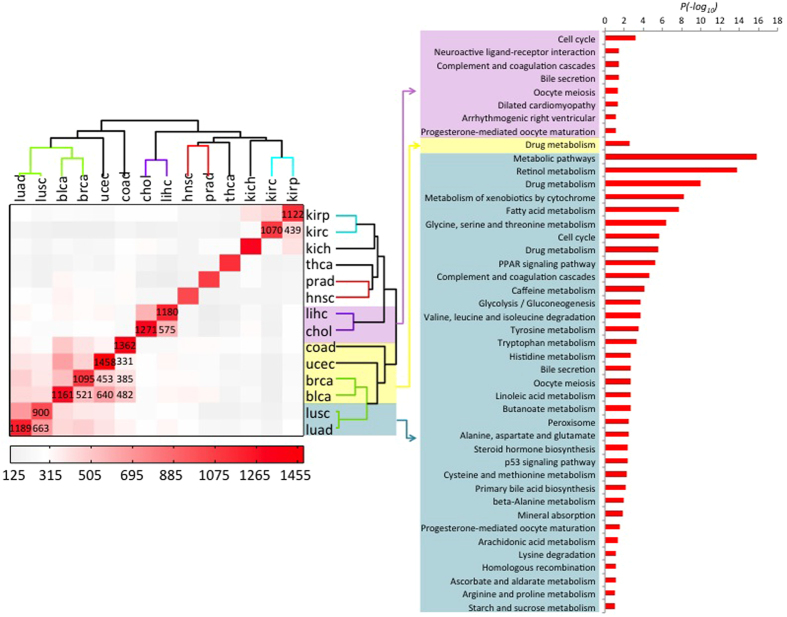
Multi-cancer identification of switch genes nearest-neighbor nodes in correlation network. Comparative analysis of switch genes nearest-neighbor nodes in a panel of 14 common cancer types whose expression data was available from TCGA. [Left] The distance matrix computed based on Hamming distance of binary-encoded (1 = present; 0 = absent) annotation of switch genes nearest-neighbor nodes across cancers is rendered in a symmetrical heat map where decreasing distance is rendered in a white to red scale. Dendrograms on columns and rows of the distance matrix indicate cancer clustering based on Hamming distance. [Right] Gene Ontology terms (www.geneontology.org) enrichment analysis for the nearest-neighbor nodes of switch genes shared by the indicated clusters of cancer types. Abbreviations. blad: bladder urothelial carcinoma; brca: breast invasive carcinoma; chol: cholangiocarcinoma; coad: colon adenocarcinoma; hnsc: head and neck squamous cell carcinoma; kich: kidney chromophobe; kirc: kidney renal clear cell carcinoma; kirp: kidney renal papillary cell carcinoma; lihc: liver hepatocellular carcinoma; luad: lung adenocarcinoma; lusc: lung squamous cell carcinoma; prad: prostate adenocarcinoma; thca: thyroid carcinoma; ucec: uterine corpus endometrial carcinoma.

As part of the analysis, the SWIM algorithm also identifies the list of nearest-neighbor genes for each switch node in the given cancer network. Similarly to the multi-cancer atlas of switch genes, we compared lists of nearest-neighbors of switch nodes across the panel of 14 cancer types obtained from TCGA by using Hamming distance to evaluate cancer similarity based on shared nearest-neighbors (Fig. 4 - Left). Interestingly, analysis of their functional annotations (Fig. 4 - Right) highlighted general association of these nearest-neighbors of switch nodes to metabolic processes. Of note, metabolic rewiring is a more and more appreciated mark of tumor transformation^37^.

### Cancer-recurrent switch genes impinge upon cell cycle and proliferation

**Figure 5:**
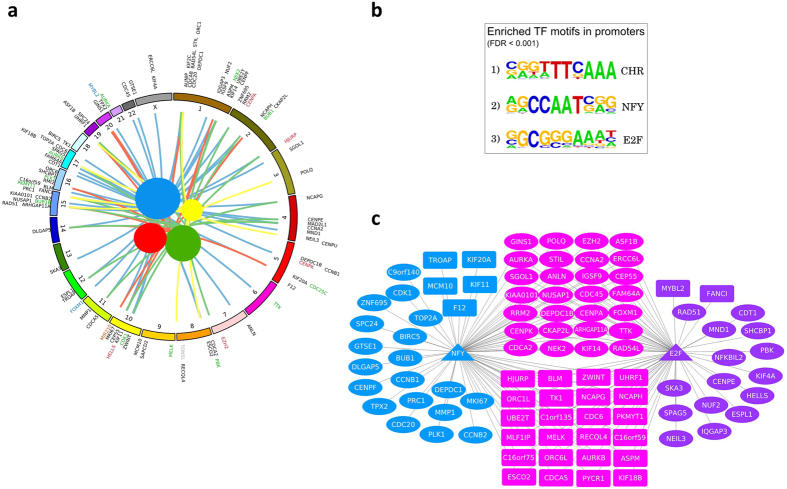
Cancer-recurrent switch genes are involved in fundamental cell regulatory pathways. (**a**) Circos (www.circos.ca) plot showing the key functional annotations, and molecular categories characterizing the subset (N = 100) of switch genes found to be recurrent across four tumors (brca, lusc, luad, ucec). Human chromosomes are arranged around the circle starting from the top at chromosome 1 (brown) and continuing clockwise. Functional annotation to significantly enriched categories are shown as colored lines emerging from differently colored spheres (blue = cell cycle; green = oocyte meiosis; red = progesterone-mediated oocyte maturation; yellow = p53 signaling pathway) to their cognate gene chromosome location. The diameter of the spheres inside the circle is proportional to their significance in the hypergeometric test used to assay enrichment. Different gene label colors indicate different molecular categories, as follows: blue = transcription factor; purple = transcription co-factor; red = chromatin remodeling factor; green = kinase/phosphatase; orange = miRNA. (**b**) Logo plots for statistically significant enriched motifs found by using the Homer software (http://homer.salk.edu/homer/) to analyze the promoter of the subset (N = 100) of cancer-recurrent switch genes. (**c**) Gene regulatory network for NFY and E2F predicted target genes among the list of cancer-recurrent switch genes. Nodes are cancer-recurrent switch genes (rectangle and oval nodes), NFY and E2F (triangle nodes). A link occurs between a given switch gene and NFY and/or E2F if the corresponding motif was found in the promoter region of the given switch gene. Node coloring legend. Blue: NFY predicted targets; violet: E2F predicted targets; magenta: NFY/E2F putatively co-regulated genes. Node shaping legend. Triangle: transcription factor; Oval: target genes with CHR motif in their promoter region; Squared: target genes without CHR motif in their promoter region.

We sought to analyze the functions of the prevalent set of 100 switch genes identified as recurrent across multiple tumors (namely, breast invasive carcinoma, lung adenocarcinoma, lung squamous cell carcinoma and uterine corpus endometrial carcinoma). To this end, we used different sources of gene annotation to map this cancer-recurrent set of switch genes to molecular function and evaluate statistically significant association to specific cellular pathways (see Fig. 5a). This analysis pinpointed several gene regulatory activities in this set such as transcription regulators/co-regulators, chromatin remodeling factors, kinases, and phosphatases. Interestingly, functional enrichment analysis allowed to relate involvement of these switch genes to core cellular pathways such as the cell cycle and the control of DNA integrity orchestrated by TP53.

Next, we investigated possible co-regulation of this set of switch genes first of all by performing a *de novo* motif enrichment analysis in their promoter regions by using Homer software^38^. The results unveiled statistically significant enrichment for three motifs, closely resembling the cell cycle gene homology (CHR) element^39^, the NF-Y binding motif CCAAT-box^40^ and the E2F binding motif^41^ (see Fig. 5b). Remarkably, the gene regulatory network that can be built based on occurrence of these three enriched regulatory motifs in promoter regions shows high coverage (*i.e.* 30% of the cancer-recurrent switch genes harbor all the three motifs in their promoter regions, 62% two of the three motifs, and 8% only one motif) and suggests extensive co-regulation (Fig. 5c). Interestingly, this high level of interconnection in the network of target genes is in line with the shared commitment of CHR, NF-Y and E2F regulatory elements in the regulation of progression through the cell cycle and its cross-talk with DNA integrity checkpoints^39^. In turn, this evidence of promoter motif enrichment reinforces the relevance of the finding that switch genes show enriched functional annotation to both these fundamental cellular pathways.

The results obtained by the *de novo* motif enrichment analysis were confirmed by Pscan, that evaluates enrichment of known binding motifs in promoter regions, employing the JASPAR 2016 motif collection^42^. Significant enrichment was found for all NF-Y associated motifs, E2F bound motifs (E2F1/E2F4/E2F6), CHR (JASPAR 2016 motif LIN56), together with other GC rich binding sites (NRF1, SP1/SP2). To confirm these results, we submitted the 100 cancer-recurrent switch genes to Cscan^43^, a tool that, starting from a large collection of ChIP-Seq experiments, performs an enrichment analysis for the transcription factors bound to the promoters of a set of input genes. We found that, among factors of the E2F family, E2F4 can be considered to be bound *in vivo* to virtually all the promoters, whereas NF-Y is bound to about 70–90% of the promoters according to the cell lines. Lower but nevertheless significant association was found also for SP1 and SP2. In other words, the regulatory associations that could be inferred from the motif analysis were largely confirmed by *in vivo* experimental data, on cancer cell lines as well.

By focusing on the GM12878, K562, and HeLaS3 cell lines (for which binding data for both E2F4 and NF-Y are available), we also found a significant enrichment for binding of FOS, CHD2 and RFX5, despite the fact that their motifs were not over-represented in the promoter analysis. Indeed, a recent work focused on regulatory modules containing NF-Y showed for all these latter factors a significant genome-wide co-association in DNA binding with NF-Y^44^. More importantly, they lack their canonical binding site when found bound to DNA in the same regions of NF-Y, where the tethering motif on DNA seems to be made of two consecutive CCAAT-box sites (bound by NF-Y) with a precise spacing. Significant genome wide co-association with NF-Y was also reported in the same study for E2F4, SP1, and SP2.

We thus investigated whether the double-CCAAT box module could be present in the promoters of the cancer-recurrent switch genes by using PscanChip^45^ on peaks for NF-YB in the K562 cell line. We indeed found that this module is contained in about half of the promoters. Moreover, a significant enrichment for E2F motifs could be found as well, situated outside the NF-Y bound regions (*i.e.* the double-CCAAT box module), suggesting no overlap, but rather a side-by-side arrangement in binding between the two transcription factors (see Supplementary Fig. 12).

## Discussion

Cancer is a leading cause of disease worldwide. In 2012 there were an estimated 14.1 million new cases of cancer in the world^46^. It means that every two seconds someone in the world is diagnosed with cancer and this number is expected to further increase in the coming decades. From a global perspective, focusing on cancer studies is therefore strongly justified for what concern both the prevention activities and the development of computational models that could support clinicians towards targeted interventions. Moreover, in cancer studies, the identification of a specific group of genes whose expression is related to a specific tumor or patient survival - that in clinical context are often referred to as clinical biomarkers - is a crucial step toward understanding the underlying molecular mechanisms and identifying novel therapeutic targets.

Taking advantage from the vast human cancer data available from TCGA^11^, we used SWIM to unveil switch genes in several human cancers (see Supplementary Table 1) as a new approach to prioritise clinically relevant genes that deserve further investigation as putative clinical biomarkers. Importantly, we found that the switch genes shared by different cancer types are all up-regulated in tumor tissues and primarily involved in the regulation of cell cycle, which is a fundamental and tightly controlled process under physiological circumstances. However, the criticality of this process becomes even more apparent from endless evidence of its alteration in pathological conditions related to uncontrolled cell growth, with tumor transformation being the most notable example. One central mechanism regulating progression through the cell cycle is phosphorylation operated by complexes of cyclin-dependent kinases (CDKs) and their corresponding cyclins. An example of this regulatory paradigm is provided by the cyclin B1 (CCNB1) and cyclin-dependent kinase 1 (CDK1) pair that controls the checkpoint between G2 phase and mitosis (M). Of note, we found both CDK1 and CCNB1 in the list of switch genes shared by different cancer types. Interestingly, the motif analysis of promoter regions conducted over this list of cancer-recurrent switch genes evidenced the presence of a neat enrichment of three DNA motifs all related to regulation of cell cycle: the CHR element, and the NF-Y and E2F binding motifs. In particular, E2F4 appeared as the best candidate of the E2F family to bind the E2F motif, since it has been found to be bound *in vivo* to virtually all the promoters.

NF-Y is known to associate with and positively regulate transcription at CCAAT motifs in the promoters of cell cycle-regulatory genes^44^,^47^. A recent bioinformatics analysis showed a remarkable abundance of CCAAT boxes in promoters regulated during the G2/M phase^48^ and a recent computational algorithm, developed to unveil transcription factors that preferentially act together, identified the NF-Y/CHR module as predictive of G2/M genes^49^. In addition, a comprehensive effort to identify genome-wide regulatory modules resulting from the co-association of NF-Y with other transcription factors has been carried out by the authors in ref. 44 through the integration of hundreds of ENCODE ChIP-Seq experiments in three different cell lines. The results of the analysis show that, among the others, E2F4 and NF-Y are constitutively expressed factors and NF-Y is often required for E2F4 efficient promoter association.

Many studies that over the last years focused on the role of NF-Y in controlling cell proliferation^47^,^50^,^51^,^52^,^53^,^54^ converge on these main results: (i) NF-Y controls the expression of several key regulators of the cell cycle; (ii) NF-Y silencing impairs G2/M progression and induces apoptosis; (iii) widespread activation of G2/M and anti-apoptotic genes requires NF-Y; (iv) NF-Y and mutant p53 physically interact up-regulating the expression of many cell cycle related genes in response to DNA damage; (v) NF-Y over-expression increases the proliferation of cancer cells harboring endogenous mutant p53. Moreover, several clinical studies have indicated that patients with up-regulated expression of NF-Y target genes have poor prognosis in multiple cancers, including breast and lung^55^,^56^,^57^.

Regarding E2F4, even though it was previously known as a repressor of cell proliferation, some studies introduced the possibility that E2F4 may function as an activator of genes implicated in positive regulation of the cell cycle, among them MYBL2^58^. In addition, E2F4 over-expression in transgenic mice leads to the development of tumors and mutated E2F4 has been reported in various human tumors, providing evidence for the oncogenic activity of E2F4^59^,^60^,^61^. Finally, CHR element is not only necessary for the repression of gene transcription in G0/G1, but also for the activation of cell cycle genes during G2 and M phases, by a direct binding with MYBL2 (B-MYB) and FOXM^39^,^62^, two pro-oncogenic genes that we have found in the list of cancer-recurrent switch genes.

**Figure 6:**
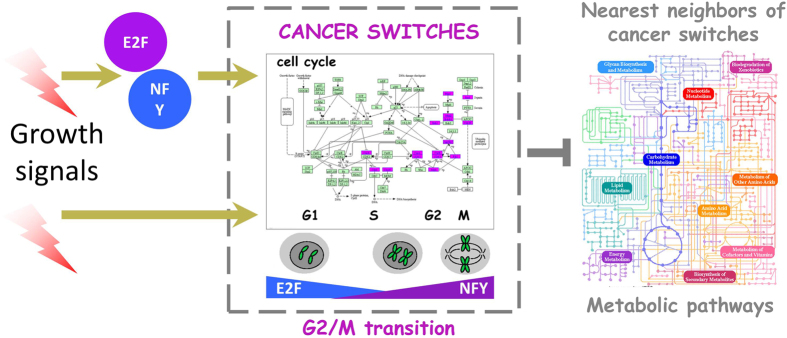
Model for regulation of cancer switch genes common to four cancer types. A set of 100 cancer-recurrent switch genes across four tumors (brca, lusc, luad, ucec) showed a marked functional annotation enrichment in cell-cycle related terms, specifically regulation of the G2-to-M transition. Promoter motif analysis suggested that two major transcription factors (namely E2F and NFY), already known to play key roles in cell cycle regulation and transformation, lead to the activation of the switch gene layer of gene regulation. Activation of switch genes in these cancer seem to predominantly repress several metabolic pathways, possibly leading to the well-known metabolic rewiring characterizing cancer cells.

Taken together these results suggest a model wherein NF-Y, in collaboration with E2F4 and/or MYBL2 and FOXM1 complexes, binds to and activates transcription of E2F/NF-Y/CHR dependent switch genes accelerating late phase of cell cycle with a consequent increase of cancer progression and rewiring of some metabolic pathways, hallmarks of the malignant transformation (Fig. 6).

In conclusion, although our study can be considered as a starting point, and further functional and clinical investigations are needed, the switch gene signatures and their nearest-neighbor genes can improve our knowledge of the cellular events that are crucial for carcinogenesis and they also reveal many potential prognostic and novel therapeutic targets that have so far not been linked to cancers. These findings put the spotlights on the importance of having a tool like SWIM that is amenable to automatically detect switch genes (*i.e.* without the intervention of a domain expert) in biologically relevant scenarios, including but not limited to human cancer. SWIM could allow clinicians and molecular biologists to avoid a waste of time and money focusing their efforts only on a small pool of genes.

## Methods

### SWIM software

The integrated network analysis developed to identify switch genes in TCGA human cancer dataset was implemented by the software SWIM, a user-friendly, wizard-like, Graphical User Interphase (GUI) developed in MATLAB^®^. A fully comprehensive description of the operating principles of SWIM is provided in Supplementary File 1.

### Tumor expression data

Collections of tumor expression data from high-throughput RNA- and miRNA-sequencing were downloaded from the TCGA data portal on 6 December 2014. High-throughput RNA-sequencing data correspond to level 3 data (*i.e.* normalized expression data) from RNASeq Version 2 created by using MapSplice to do the alignment and RSEM to perform the quantification and normalization. MiRNA-sequencing data correspond to level 3 data (*i.e.* normalized expression data) created by using RPKM procedure to perform the normalization. Additional details on the downloaded samples such as dataset release and platform type are provided in Supplementary Table 5. Only cancer datasets including at least 7 patients with tumor and matched-normal samples (*i.e.* the matched-normal tissue is defined as the tissue that is adjacent to the tumor and taken from the same patient) for both RNA- and miRNA-sequencing experiments were retained for subsequent analysis. This data selection left a total of 2108 samples relative to 14 tumor types (namely, brca, kirc, thca, prad, lihc, hnsc, lusc, kich, kirp, blca, luad, chol, ucec and coad) and accounting for 540 unique cancer patients. Clinical metadata for the same patients were also retrieved through the TCGA.

### Data annotation and functional enrichment analysis

Gene features included in the RNA-sequencing cancer datasets were annotated to molecular biotype (*e.g.* protein coding, non-coding RNA) by using the human gene annotation available from Ensembl (release GRCh38). Moreover, genes were mapped to selected gene categories (*e.g.* transcription factor, chromatin remodeling factor), cellular pathways and regulatory interactions (such as transcription factor targeting predictions) by using several gene annotation sources^63^,^64^,^65^. Fisher’s exact test has been used to analyze associations between selected gene subsets (*e.g.* switch, non-switch) and functional annotation terms such as gene ontology (GO) terms^63^ and KEGG pathways^64^, or between selected gene subsets and the occurrence of specific regulatory motifs, such as transcription factor binding sites, in promoter regions. Motif enrichment analysis in promoter regions was performed by using the Homer software^38^. Promoter regions used in this analysis were identified as genomic regions spanning intervals from −500 to +50 nucleotides with respect to transcription start sites. A p-value 0.05, after adjustment for multiple testing performed with the Benjamini-Hochberg method, was set as the significance threshold to identify enriched functional annotations and regulatory motifs among selected gene lists. In order to confirm the results obtained with Homer software we used the Pscan tool^42^ to analyze the promoter regions (from −450 to +50 with respect to the transcription starting site) of the RefSeq transcripts associated with the cancer-recurrent switch genes.

Moreover, we performed an analysis on the same promoter set of the cancer-recurrent switch genes with the Cscan tool^43^. Starting from a large collection of ChIP-Seq experiments for transcription factors (including the whole ENCODE dataset), Cscan determines for each input promoter which ChIP-Seq peaks can be found associated with it. Then, it computes for each ChIP-Seq experiment considered the significance of its enrichment in the input promoter set, according to how many promoters of the set contain a peak for the experiment, with respect to the fraction of annotated genes in the genome with a peak for the experiment in the promoter.

In order to investigate whether double-CCAAT box module could be present on the promoter set of the cancer-recurrent switch genes, we performed a sequence analysis of the ChIP-Seq peak regions for both NF-Y (peaks of the ChIP-Seq for NF-YB) and E2F4 that could be found in the promoter regions in the K562 cell line using PscanChIP^45^. PscanChIP is a motif analysis tool that, starting from a collection of genomic regions derived from a ChIP-Seq experiment, scans them using motif descriptors like JASPAR or TRANSFAC position-specific frequency matrices, or descriptors uploaded by users, and it evaluates both motif enrichment and positional bias within the regions according to different measures and criteria. PscanChIP can successfully identify not only the actual binding sites for the TF investigated by a ChIP-Seq experiment but also secondary motifs corresponding to other TFs that tend to bind the same regions, and, if present, precise positional correlations among their respective sites^45^.

### Kaplan-Meier

To analyze the prognostic value of each switch gene in the TCGA dataset of breast invasive carcinoma, the patient samples are split into two groups (called low-expression and high-expression group) according to the upper and lower quartile expressions of the proposed biomarker switch gene. In particular, low- and high-expression groups refer to patients with expression levels of the given switch gene lower than the 25^*th*^ and greater than the 75^*th*^ percentile, respectively. For each patient cohort, the cumulative survival rates are computed for each switch gene according to the Kaplan-Meier method^66^ on the clinical metadata provided by TCGA. A log-rank test was performed to assess the p-value and to signify that the lower the p-value, the better the separation between the two prognosis groups. Finally, the switch genes are sorted by increasing p-values in order to identify the best at separating the two prognosis groups (see Supplementary Table 2).

## Additional Information

**How to cite this article**: Paci, P. *et al*. SWIM: a computational tool to unveiling crucial nodes in complex biological networks. *Sci. Rep.* **7**, 44797; doi: 10.1038/srep44797 (2017).

**Publisher's note:** Springer Nature remains neutral with regard to jurisdictional claims in published maps and institutional affiliations.

## Supplementary Material

Supplementary Files
