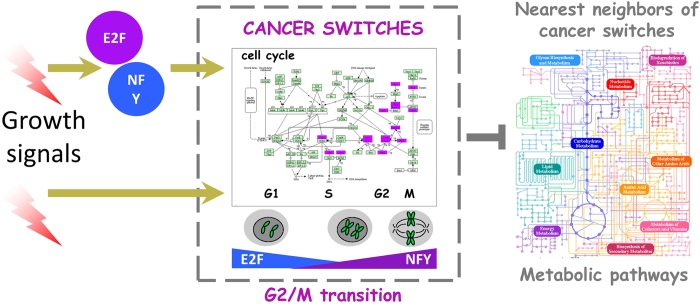# Erratum: SWIM: a computational tool to unveiling crucial nodes in complex biological networks

**DOI:** 10.1038/srep46843

**Published:** 2017-06-16

**Authors:** Paola Paci, Teresa Colombo, Giulia Fiscon, Aymone Gurtner, Giulio Pavesi, Lorenzo Farina

Scientific Reports 7: Article number: 44797; 10.1038/srep44797 published online: 03 20 2017; updated: 06 16 2017.

In the HTML version of this Article, Figure 6 was incorrect. The correct Figure 6 appears below as Figure 1.

This error has been corrected in the HTML version of the Article; the PDF version was correct at the time of publication.

## Figures and Tables

**Figure 1:**