# Supplementary material for: SWIM: a computational tool to unveiling crucial nodes in complex biological networks
Source: Sci Rep. 2017 Mar 20;7:44797. doi: 10.1038/srep44797 (PMC5357943; doi:10.1038/srep44797)
Supplement: Supplementary Files [file srep44797-s1.pdf]

SWIM: a computational tool to unveiling crucial nodes in complex biological networks

Paola Paci[1,2,*], Teresa Colombo[1], Giulia Fiscon[1], Aymone Gurtner[3], Giulio Pavesi[4], and Lorenzo Farina[5]

[1]Institute for Systems Analysis and Computer Science “Antonio Ruberti”, National Research Council, Rome, Italy
[2]SysBio Centre for Systems Biology, 00185 Rome, Italy
[3]Department of Research, Advanced Diagnostics, and Technological Innovation, Translational Research Area, Regina Elena National Cancer Institute, Rome, Italy
[4]Department of Biosciences, University of Milan, Italy
[5]Department of Computer, Control and Management Engineering, “Sapienza” University, Rome, Italy
[*]paola.paci@iasi.cnr.it

ABSTRACT

SWItchMiner (SWIM) is a wizard-like software implementation of a procedure, previously described, able to extract information contained in complex networks. Specifically, SWIM allows unearthing the existence of a new class of hubs, called “fight-club hubs”, characterized by a marked negative correlation with their first nearest neighbors. Among them, a special subset of genes, called “switch genes”, appears to be characterized by an unusual pattern of intra- and inter-module connections that confers them a crucial topological role, interestingly mirrored by the evidence of their clinic-biological relevance.
Here, we applied SWIM to a large panel of cancer datasets from The Cancer Genome Atlas, in order to highlight switch genes that could be critically associated with the drastic changes in the physiological state of cells or tissues induced by the cancer development. We discovered that switch genes are found in all cancers we studied and they encompass protein coding genes and non-coding RNAs, recovering many known key cancer players but also many new potential biomarkers not yet characterized in cancer context. Furthermore, SWIM is amenable to detect switch genes in different organisms and cell conditions, with the potential to uncover important players in biologically relevant scenarios, including but not limited to human cancer.

Supplementary Material

This document is composed of the Supplementary File 1 (User Guide) and the Supplementary File 2 - (Supplementary Figures) cited in the main text.

Supplementary File 1 - User Guide

In the following, the reader will find a detailed user guide that shows how SWIM works and how to use it.

User Guide

SWIM

SWItchMiner

How does it work and how to use it

User Guide 2016

About SWIM

Copyright © 2016 Paola Paci

SWIM is free software: you can redistribute it and/or modify it under the terms of the GNU General Public License as published by the Free Software Foundation, either version 3 of the License, or (at your option) any later version.

SWIM is distributed in the hope that it will be useful, but WITHOUT ANY WARRANTY; without even the implied warranty of MERCHANTABILITY or FITNESS FOR A PARTICULAR PURPOSE. See the GNU General Public License for more details.

You should have received a copy of the GNU General Public License along with SWIM. If not, see <http://www.gnu.org/licenses/>.

About this guide

Copyright © 2016 Paola Paci, Giulia Fiscon

This document is covered by the GNU General Public License as published by the Free Software Foundation, either version 3 of the License, or (at your option) any later version.

You should have received a copy of the GNU General Public License along with SWIM. If not, see <http://www.gnu.org/licenses/>.

Table of contents

Getting Started 4

Software requirements 4

Setting up 4

Folders architecture 4

SWIM **Basics** 6

Algorithm steps 6

Input files 9

Output files 10

Usage Example 12

Aim 12

Dataset 12

Getting started 12

Folders architecture 12

Input files 13

Running SWIM 14

Output files 30

References 36

Contacts 36

How to cite SWIM 36

Glossary 37

Getting Started

SWItchMiner (`SWIM`) is a software for the identification of a small pool of genes, called *switch genes*, which are likely to be critically associated with drastic changes in many biological settings. This procedure was set in [Palumbo et al., 2014], where switch genes were found to be master regulators of the previously reported transcriptome remodeling that marks the developmental shift from immature to mature growth in grapevine [Fasoli et al., 2012]. In the present manuscript, switch genes have been investigated in different human cancer types and the results strongly support the hypothesis of their key role in cancer development.

Software requirements

`SWIM` has been developed in MATLAB®[1] (version R2013a including the Bioinformatics and Statistics Toolboxes) and tested on the following operative systems:

- OSX 10.9.5
- GNU/Linux Ubuntu 14.04
- Windows 10 Pro

Setting up

- Install MATLAB® and the Bioinformatics and Statistics Toolboxes
- Download and unzip the compressed file *SWIM*.zip that is available as supplementary material of the present manuscript. This will create a folder named *SWIM* in the current directory.

Folders architecture

The *SWIM* folder contains the following files and directories:

- run.m: it is the main script
- the *script* folder: it stores all MATLAB® scripts called by run.m
- the *project* folder: it stores all required data to perform the trial run explained in the Usage Example section.

The files and folders architecture of `SWIM`[2] reflects its settings that are based on the concept of projects and datasets. Here, a project represents a thematic container that can include one or more datasets all related to the same theme. Hence, the *project* folder will contain as many folders as the number of the different projects the user may wish to add, each freely named by the user. A folder named *dataset* (mandatory folder name) must be included in each folder representing a specific project added by the user. The *dataset* folder will contain as many folders as the number of the different datasets the user may wish to analyze for a given project, each freely named by the user. Two folders named *list* and *matrix* (mandatory folder names) must be included in each folder representing a specific dataset that will be analyzed by the user for a given project (see Figure UG1).

The exemplar project (named *sample_project*) included in the *project* folder regards the analysis of high-throughput sequencing expression data for breast invasive carcinoma (brca) downloaded from The Cancer Genome Atlas (TCGA) [Weinstein et al., 2013]. This example allows the user to get the hang of the parent/child directories structure of `SWIM` that must be reproduced for any further project the user may wish to add. In particular, *sample_project* contains (see Figure UG1):

---

[1] MATLAB is a registered trademark of The MathWorks, Inc (http://www.mathworks.com/products/MATLAB/)
[2] Note that the italic font *SWIM* indicates the directory, while `SWIM` indicates the software

- the *dataset* folder (mandatory folder name): it holds a folder freely named *brca*. If the exemplar project had included more than one dataset, these would have been placed in this directory as separated folders with customized names.
    - the *brca* folder (customized folder name): it represents the exemplar dataset and holds the data required to perform the trial run stored in the following folders:
        - ✓ the *matrix* folder (mandatory folder name): it stores the input data matrix related to the given dataset. Note that `SWIM` requires two conditions (A and B) between which searching for switch genes, and the data matrix must include data for both conditions.
        - ✓ the *list* folder (mandatory folder name): it stores the lists of the samples concerning the condition A and B in two separated files.

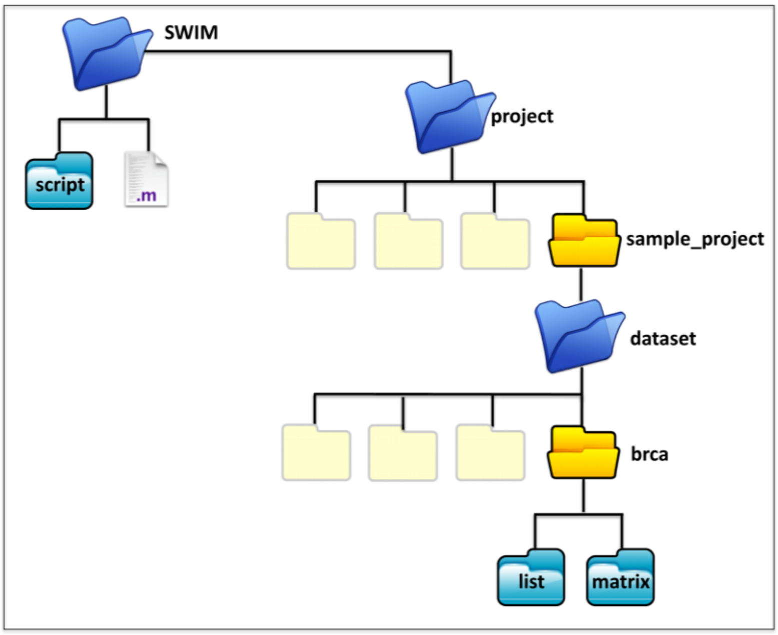

Figure UG1. `SWIM` folders architecture. The structure of the parent/child directories of `SWIM` is illustrated in the *sample_project* folder. This structure must be mirrored in any folder (pale yellow folders with freely customized names) the user may wish to add within the main *project* folder. In particular, *sample_project* contains a folder named *dataset* (mandatory folder name) with a folder freely named *brca* that represents the exemplar dataset. It holds two folders with mandatory names, *list* and *matrix*, including all data required to perform the trial run (see the Usage Example section). If the exemplar project had included more than one dataset, these would have been placed at the same level of *brca* as separated folders with customized names (pale yellow folders) and would have included the two folders *list* and *matrix*.

SWIM Basics

Algorithm steps

SWIM provides a user-friendly, wizard-like, Graphical User Interphase (GUI). The wizard presents a sequence of dialog boxes that lead the user through a series of well-defined steps depicted in Figure UG2.

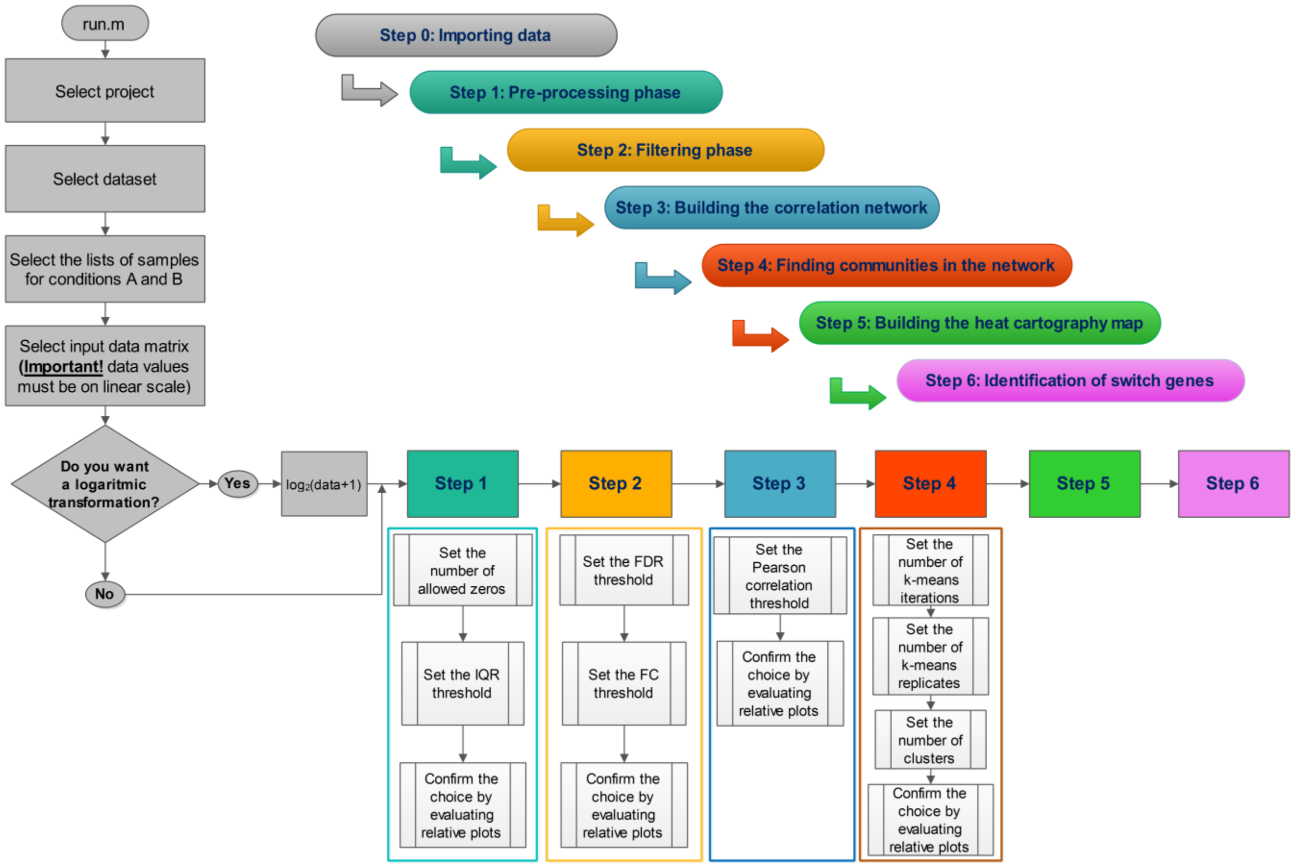

Figure UG2. Flowchart of SWIM. The figure depicts the steps performed by SWIM and detailed in the text. Steps 1-4 require the selection of specific thresholds as outlined in the corresponding boxes. Note that SWIM expects input data on linear scale. The choice of not applying the logarithm transformation is not meant to allow the user to upload already log-transformed data. Rather, this is an option regarding special cases in which the user wishes to perform the entire analysis on the linear scale. Legend: FC= fold-change; FDR=False Discovery Rate; IQR=Inter Quartile Range.

Step 0: Importing data

This step requires the selection of the input files in order to start the data analysis (see Input files section).

Step 1: Pre-processing phase

Denoting by S the total number of samples (S = samples in the condition A + samples in the condition B), this step requires the selection of two specific thresholds for removing genes whose expression across the S samples is mostly zero or change very little. The first threshold regards the maximum number of samples out of S allowed to be equal to zero. The second threshold concerns the minimum variation - measured by the Inter Quartile Range (IQR) percentile - allowed for each gene across the S samples.

Step 2: Filtering phase

This step requires the selection of two specific thresholds for removing genes whose expression between the two given conditions (A and B) does not change enough or does it without statistical significance. Considering the logarithm of the ratio between the average expression of samples in condition A and the average expression of samples in condition B (log fold-change), the first threshold allows to remove the genes falling behind, in absolute value, a fixed cutoff on the log fold-change. The second threshold concerns the smallest probability (p-value) for which the data allow to reject the null hypothesis (i.e., the means of the two distributions – normal and cancer - are identical) of the Student's t-test. Actually, since this statistical test will be repeated multiple times (as many as the genes under testing), the obtained p-values must be adjusted. To correct multiple tests, `SWIM` makes use of False Discovery Rate (FDR) method [Benjamini and Hochberg, 1995] and thus the threshold refers to the FDR values. At end of this phase, the differentially expressed genes between conditions A and B have been identified.

Step 3: Building the correlation network

This step requires the selection of a threshold for building the correlation network where two nodes are connected if the absolute value of the Pearson correlation between their expression profiles exceeds a given cutoff. This threshold should reflect a right balance between the number of edges and the number of connected components of the network: the number of edges should be as small as possible in order to have a manageable network (pointing towards a higher threshold) and the number of connected components should be as small as possible in order to preserve the integrity of the network (pointing towards a smaller threshold).

Step 4: Finding communities in the network

To find communities in the network, `SWIM` makes use of the k-means algorithm [Hartigan et al.,1973], a method of cluster decomposition whose aim is to partition n objects (i.e., the nodes of the co-expression network) into N clusters. The goal of the clustering is expressed by an objective function that depends on the proximities of the nodes to the cluster centroids.

As objective function, which measures the quality of a clustering, `SWIM` uses the Sum of the Squared Error (SSE), defined as follows:

$$SSE = \sum_{i=1}^{N} \sum_{x \in C_i} dist(c_i, x)^2 \quad (1)$$

where N is the number of the clusters, C_i is the ith cluster, x is a node in the ith cluster, c_i is the centroid of the ith cluster. The centroid c_i is given by:

$$c_i = \frac{1}{m_i} \sum_{x \in C_i} x_i \quad (2)$$

where m_i is the number of nodes in the ith cluster. There are as many centroids as the number of the clusters.

As measurement of the proximities of two nodes, `SWIM` makes use of the following metrics:

$$dist(x, y) = 1 - \rho(x, y) \quad (3)$$

where ρ is the Pearson correlation coefficient between the expression profiles of the nodes x and y. Thus, two nodes are close in the network ($dist = 0$) if they are highly correlated ($\rho = +1$) on the contrary they are far apart in the network ($dist = 2$) if they are highly anti-correlated ($\rho = -1$). The choice of this metrics is intended to emphasize the differences between correlated and anti-correlated genes. Taking advantage of this metric, `SWIM` builds the distances matrix whose generic element is given by the equation (3).

The k-means algorithm, despite being the most widely used clustering algorithm, has some intrinsic limitations. Firstly, the number of clusters must be set in advance; secondly, it guarantees convergence only to a local minimum of SSE; thirdly, the initial position of the centroids is randomly chosen causing a dependence of the partitioning on initialization. Overcoming these problems is well behind the scope of this user guide. However, some reasonable assumptions can be done and are described in the following.

There is no strict method to determine the "correct" number of clusters. Among others, `SWIM` uses an approach - named "Scree plot" - that evaluates the behavior of the SSE function to vary the number of clusters. Then, the position of an elbow in the scree plot - i.e., where the "cliff" reaches a bottom plateau - determines an appropriate number of clusters.

Since finding the global optimum of SSE is theoretically NP-hard [Meila, 2006], it is commonly assumed that is sufficient to carry out a number of random initializations followed by a selection of the best separated solution, measured by the lowest SSE [Lisboa et al., 2013]. Moreover, the partition with the lowest SSE is commonly assumed to be reproducible under repeated initializations [Lisboa et al., 2013].

Thus, for a given number of clusters, `SWIM` allows repeating the clustering many times (replicates), each with a new set of initial cluster centroid positions. For each replicate, the k-means algorithm performs iterative partitioning (iterations) until the minimum of the SSE function is reached. Then, the cluster configuration with the lowest SSE values among all replicates will be chosen, for that number of clusters.

Step 5: Building the heat cartography map

Once the modular structure of the complex network has been found, roles have to be assigned to each node. This is done by dividing the plan according to two parameters, the *clusterphobic coefficient* K_π and the *global within-module degree* z_g. The clusterphobic coefficient K_π measures the "fear" of being confined in a cluster, in analogy with the claustrophobic disorder. A high value of K_π denotes nodes having much more external than internal links. The global within-module degree z_g measures how "well connected" each node is to other nodes in its own community. In the following, the formal definitions of these parameters:

$$z_g{}^i = \frac{k_i^{in} - \bar{k}_{C_i}}{\sigma_{C_i}} \tag{4}$$

$$K_\pi = 1 - \left(\frac{k_i^{in}}{k_i}\right)^2 \tag{5}$$

where k_i^{in} is the number of links of node i to nodes in its module C_i, k_i is the total degree (i.e., number of links emanating from a node) of node i, $\bar{k}_{C_i}$ and σ_{C_i} are the average and standard deviation of the total degree distribution of the nodes in the module C_i.

This definition of z_g quantifies how much a node is a hub (i.e., degree exceeding 5 [Han et al., 2004]) in its community and thus represents a measure of local connectivity. On the contrary, the parameter K_π evaluating the ratio of internal to external connections of a node represents a measure of global connectivity. Note that $K_\pi = 0$ when a node has only links within its module, i.e., it does not communicates with the other modules

($k_i^{in} = k_i$). On the contrary, K_π is close to 1 when the majority of its links are external to its own module. The values of these two parameters define, in the plan identified by K_π and z_g, a cartography made up by seven regions corresponding to seven different roles of nodes in the network [Guimera and Amaral, 2005]:

1. *non local hub* for $z_g < 2.5$

$K_\pi = 0$	Ultra-peripheral nodes (role R1)
$K_\pi \leq 0.625$	Peripheral nodes (role R2)
$0.62 < K_\pi \leq 0.8$	Non-hub connectors (role R3)
$K_\pi > 0.8$	Non-hub kinless nodes (role R4)

2. *local hub* for $z_g \geq 2.5$

$K_\pi = 0.3$	Provincial hubs (role R5)
$K_\pi \leq 0.75$	Connector hubs (role R6)
$K_\pi > 0.75$	Kinless hubs (role R7)

Then, `SWIM` colors nodes in the cartography according to the Average Pearson Correlation Coefficient (APCC) between the expression profiles of each node and its nearest neighbors [Han et al., 2004]. We defined this representation of the network as "heat cartography map".

By computing the APCC of expression over all interaction partners of each hub in protein-protein interaction (PPI) networks in yeast, the authors in [Han et al., 2004] concluded that hubs fall into two distinct categories: date hubs that display low co-expression with their partners (low APCC) and party hubs that have high co-expression (high APCC). In most gene expression networks we studied, the distribution of APCCs appears to be trimodal (see Supplementary Fig. 1 of the present manuscript) where, similarly to PPI networks, two peaks represent low (date hubs) and high (party hubs) positive APCC values, but with the addition of a new, third peak which is characteristic of gene expression networks and represents negative APCC values. We called nodes populating this peak "fight-club hubs".

Step 6: Identification of switch genes

Looking at the heat cartography map, we called "switch genes" the subset of the fight-club hubs that mainly interact outside their community (role R4). In particular, they satisfy the following topological and expression features:

1. being not an hub in their own cluster (z_g<2.5)
2. having many links outside their own cluster ($K_\pi > 0.8$)
3. having a negative average weight of their incident links (APCC < 0)

At the end of step 6, swim gives the opportunity to perform further analyses regarding the evaluation of network robustness, which is the resilience to errors, by studying the effect on the network connectivity of removing nodes by decreasing degree. In particular, `SWIM` evaluates the effect on the average shortest path (the shortest path between two nodes is the minimum number of edges connecting them and the average shortest path of a network is the average of the shortest paths for all possible pairs of network nodes) of removing randomly chosen nodes, switch genes, fight-club hubs, date and party hubs. Since scale-free networks have few hubs and many non-hub nodes, they are amazingly resistant to a random removal of nodes, while the removal of hubs causes an effect known as "vulnerability to attack" to allude to the fact that the integrity of the network is destroyed.

Input files

`SWIM` requires as input the following files:

- data matrix: a tab-delimited file where rows are the variables and the columns are the observables. The matrix must include all data related to the two conditions (A and B) under testing as well as row

headers (variable names) in the first column and column headers (observable names) in the first row. Values in the matrix are expected to be on linear scale.

- list of names of the observables concerning condition A: a single-column file, without header, listing all samples related to condition A (one sample name per row). Note that each sample name must **exactly** match one column header in the data matrix.
- list of names of the observables concerning condition B: a single-column file, without header, listing all samples related to condition B (one sample name per row). Note that each sample name must **exactly** match one column header in the data matrix.

Examples of the data matrix and the list files are provided in the *matrix* and the *list* folders included in the *sample_project*, respectively and a detailed description is provided in the Usage Example section.

Output files

SWIM provides as output the following files and directories (see Figure UG3):

- the directory *all-switch* created in the folder of each project added by the user in the *project* folder. It includes as many text files as the number of the analyzed datasets (each called with the name of the reference dataset), with the list of switch genes for each dataset. Essentially, this directory contains a summary of all identified switch genes for all studied datasets (see Figure UG4, panel a).
- two directories (named *filtering* and *switch*) and a MATLAB® binary file (named *parameters.mat*) created in each folder (concerning a specific dataset) included in the *dataset* folder of each project added by the user:

  1. *filtering*: it includes all report files about the statistics analysis of that dataset
  2. *switch*: it includes all files about the identified switch genes of that dataset
  3. *paramters.mat*: it is a MATLAB® binary file that summarizes all the thresholds and parameters set during the run of that dataset

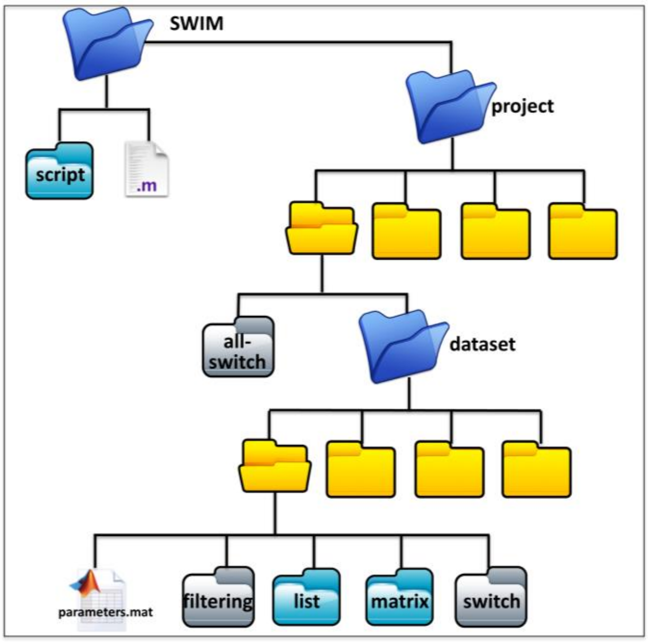

Figure UG3. Architecture of the output files and directories created by SWIM. In this figure the hierarchical structure of the directories (*all-switch, filtering,* and *switch*) obtained by running SWIM is shown. Grey folders correspond to the directories created by SWIM; the blue folders *dataset, list,* and *matrix* correspond to directories with mandatory names which must be created by the user before starting the run; yellow folders correspond to directories created and freely named by the user before starting the run. The file *parameters.mat* is created by SWIM at the end of the run.

The *filtering* folder contains three folders (see Figure UG4, panel b):

1. *figure*: it includes all the figures (in PNG and FIG format) about the statistical analyses
2. *matFile*: it includes all the MATLAB® binary files about the statistical analyses
3. *txtFile*: it includes all the text files about the statistical analyses

Similarly, the *switch* folder contains three folders (see Figure UG4, panel c):

1. *figure*: it includes all the figures (in PNG and FIG format) about the network analysis
2. *matFile*: it includes all the MATLAB® binary files about the network analysis
3. *txtFile*: it includes all the text files about the network analysis

A detailed description of the output files obtained by running `SWIM` is provided at the end of the Usage Example section.

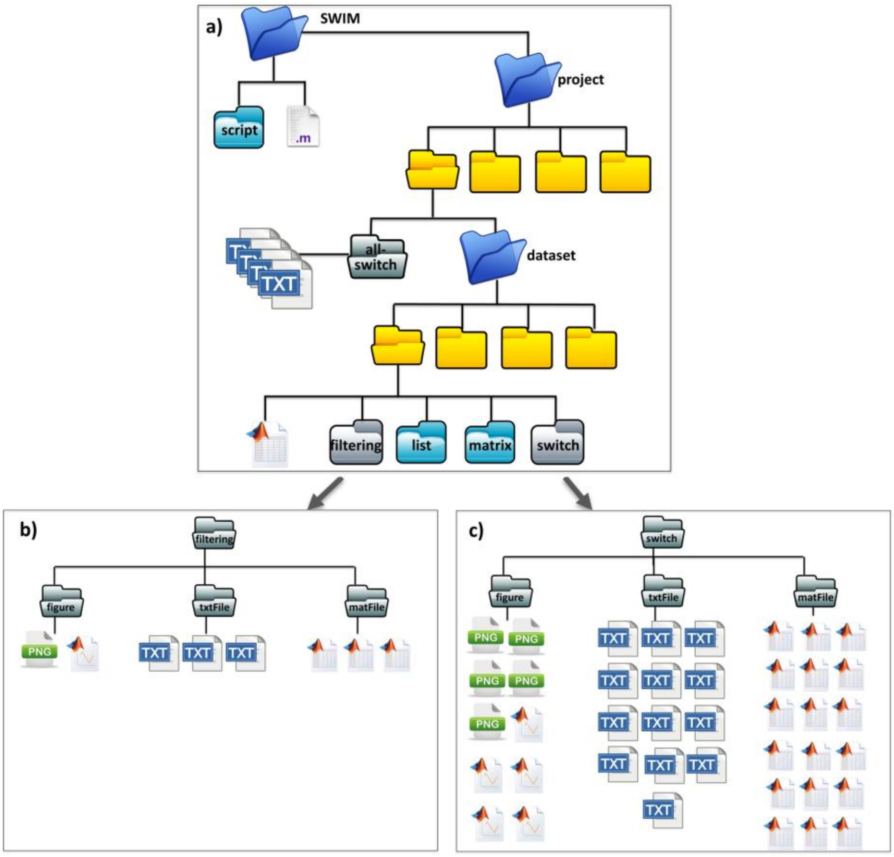

Figure UG4. Contents of the output files and directories created by `SWIM`. Panel a) illustrates the content of the *all-switch* folder - created by `SWIM` inside each project added by the user in the *project* folder. In this panel, four datasets (yellow folders) are sketched as an example, and thus four text files (one for each analyzed dataset) are created by `SWIM` within the *all-switch* folder. Panels b) and c) illustrate the structure and content, respectively, of the *filtering* and *switch* folders - created by `SWIM` inside each specific dataset of the *dataset* folder of the related project. The exact number of files (PNG, MAT, FIG, TXT) contained in the *filtering* and *switch* folders is depicted.

Usage Example

Aim

By running this tutorial, the user will learn how to use `SWIM` to identify switch genes capturing the difference between the two conditions: A) tumor sample and B) normal sample. In this tutorial, the miRNA-sequencing data are available from TCGA and thus microRNAs (miRNAs) are included in the list of eligible candidates for being switch genes.

Dataset

Data of miRNA- and RNA-sequencing samples of brca, downloaded from TCGA in December 2014, include:

- 1182 RNA-sequencing samples (of which, 1069 are tumor and 113 normal samples) relative to1062 unique patients
- 1212 miRNA-sequencing samples (of which, 1108 are tumor and 104 normal samples) relative to 1084 unique patients

Out of the whole set of patients, 103 have samples of cancer and matched normal tissues for both the RNA-sequencing (concerning protein-coding and non-coding RNAs abundance) and miRNA-sequencing.

Getting started

- Launch MATLAB® and set the *SWIM* folder as the current MATLAB® folder listed in the Address field, whose content is shown in the Current Folder window
- Type run at the prompt in the Command Window

Folders architecture

The parent/child directories structure of `SWIM` for the *sample_project* is depicted in Figure UG5.

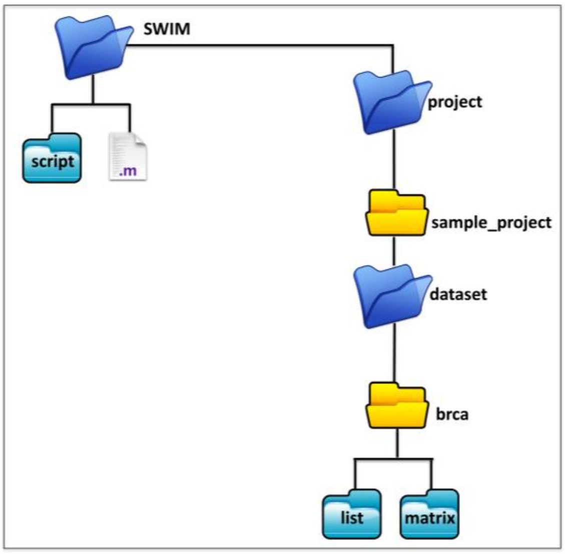

Figure UG5. Architecture of input directories for the tutorial. In this figure the initial structure of the `SWIM` folders for the example project, related to RNA-sequencing and miRNA-sequencing data obtained by TCGA for brca, is shown.

Input files

For running `SWIM`, the present tutorial require as input the following files:

- *matrix_RNASeq.txt*: a text file providing protein-coding and non-coding RNAs abundance (RNA-sequencing data matrix). The rows are the RNAs and columns are samples. The file includes row headers (TCGA gene identifiers)[3] and column headers (TCGA barcodes).
- *matrix_miRNASeq.txt*: a text file providing miRNAs abundance (miRNA-sequencing data matrix). The rows are miRNAs and columns are samples. The file includes row headers (TCGA miRNA identifiers)[4] and column headers (TCGA barcodes).
- *list_RNASeq_Tumor.txt*: a text file that lists the barcodes concerning the condition A (tumor). Each barcode exactly matches one column header of the *matrix_RNASeq.txt* file.
- *list_RNASeq_Normal.txt*: a text file that lists the barcodes concerning the condition B (matched normal). Each barcode exactly matches one column header of the *matrix_RNASeq.txt* file.
- *list_miRNASeq_Tumor.txt*: a text that lists the barcodes concerning the condition A (tumor). Each barcode exactly matches one column header of the *matrix_miRNASeq.txt* file.
- *list_miRNASeq_Normal.txt*: a text file that lists the barcodes concerning the condition B (matched normal). Each barcode exactly matches one column header of the *matrix_miRNASeq.txt* file.

Note that, in this example, the analysis is restricted to all common patients between tumor and matched normal tissues of both RNA-sequencing and miRNA-sequencing data matrices.

[3] These are composite gene identifiers provided by TCGA and have the format: <HGNC Gene Symbol | Entrez Gene Id>. The HGNC and Entrez Gene identifiers relate to the HUGO gene nomenclature committee (http://www.genenames.org/) and the NCBI Gene (http://www.ncbi.nlm.nih.gov/gene) databases, respectively.
[4] Strictly speaking, these TCGA identifiers refer to the miRNA precursors and follow the nomenclature provided by miRBase (http://www.mirbase.org/).

Running SWIM

Step 0: Importing data

A pop-up window will prompt the user to select the project folder. In this tutorial, this is the *sample_project* folder.

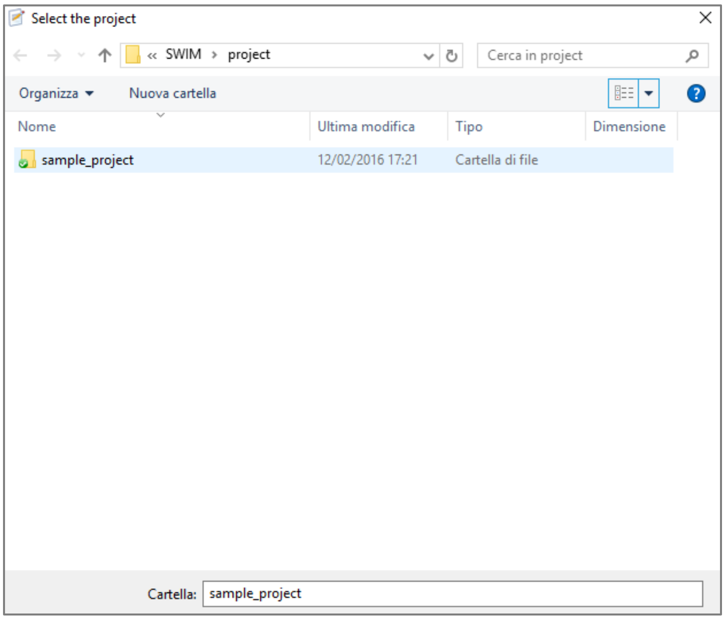

A pop-up window will prompt the user to select the dataset folder. In this tutorial, this is the *brca* folder.

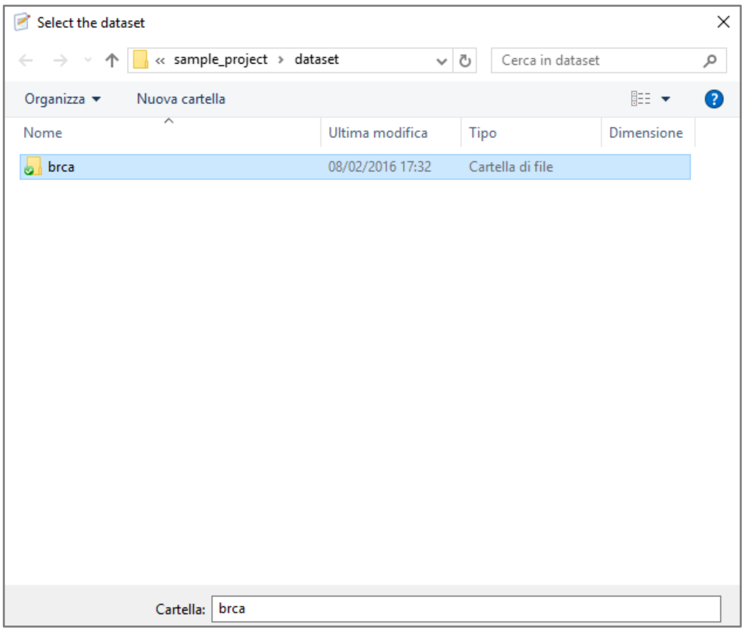

A pop-up window will ask the user to choose if miRNA data is also available.

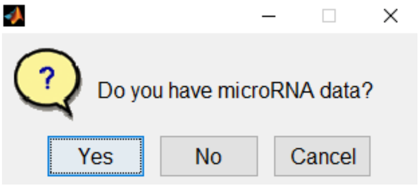

In this tutorial, choose YES[5].

A pop-up window will prompt the user to select the file listing samples of condition A for RNA-sequencing data. In this tutorial, this is the *list_RNASeq_Tumor.txt* file.

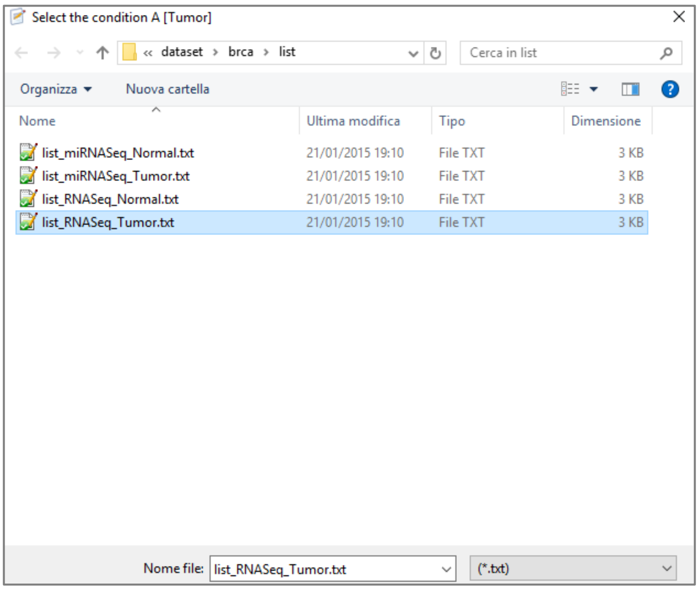

A pop-up window will prompt the user to select the file listing samples of condition B for RNA-sequencing data. In this tutorial, this is the *list_RNASeq_Normal.txt* file.

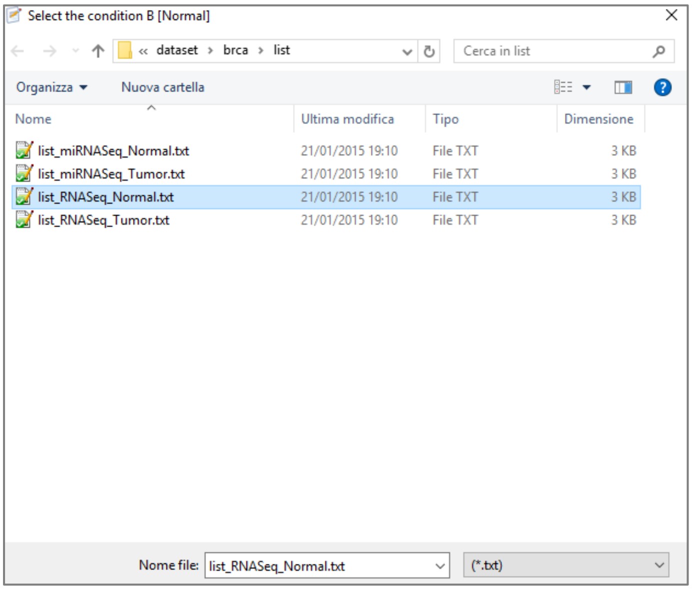

[5] In all pop-up windows of `SWIM` the Cancel choice terminates the run.

A pop-up window will prompt the user to select the data matrix (RNA-sequencing data matrix). In this tutorial, this is *matrix_RNASeq.txt*.

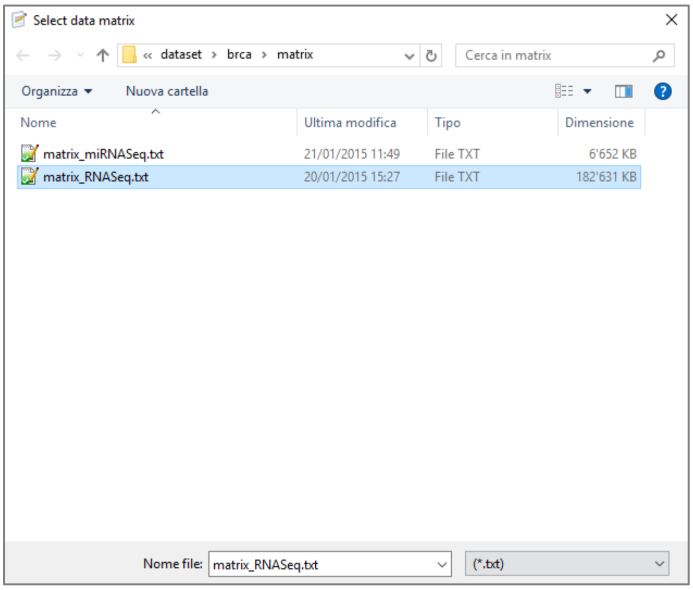

A pop-up window will prompt the user to select the file listing samples of condition A for miRNA-sequencing data. In this tutorial, this is the *list_miRNASeq_Tumor.txt* file.

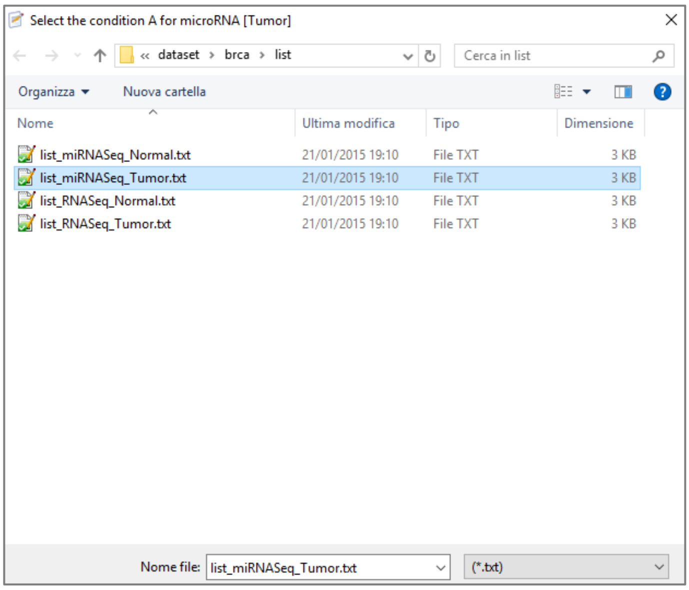

A pop-up window will prompt the user to select the file listing samples of condition B for miRNA-sequencing data. In this tutorial, this is the *list_miRNASeq_Normal.txt* file.

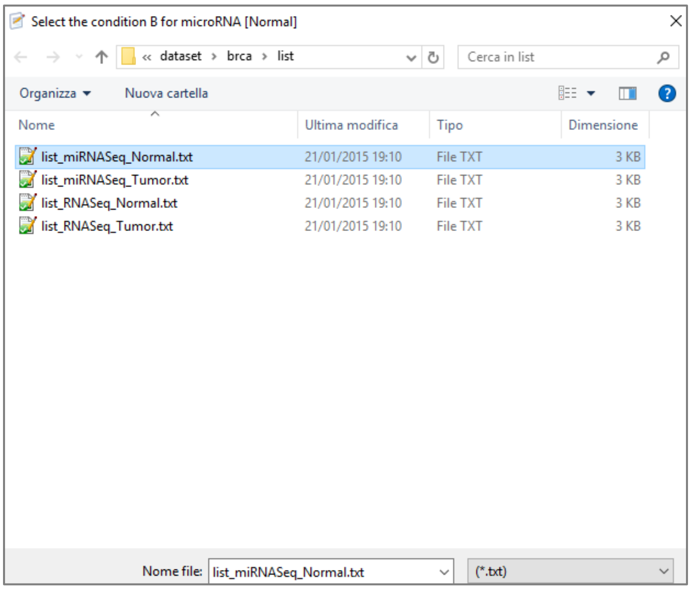

A pop-up window will prompt the user to select the miRNA data matrix (miRNA-sequencing data matrix). In this tutorial, this is *matrix_miRNASeq.txt*.

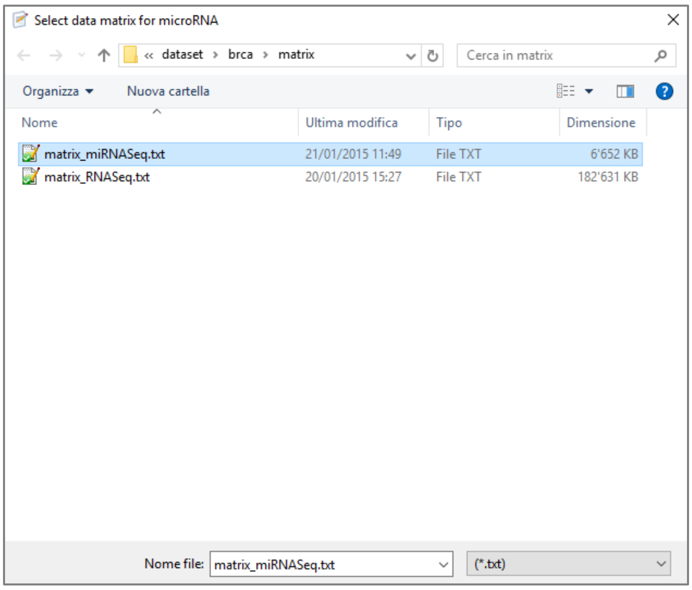

A pop-up window will ask the user to choose if perform or not the logarithmic transformation of the data. Note that `SWIM` expects data on linear scale.

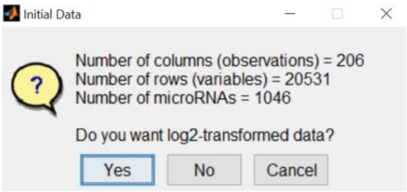

In this tutorial, choose YES. Note that the choice of not applying the logarithm transformation is not meant to allow the user to upload already log-transformed data. Rather, this is an option regarding special cases in which the user wishes to perform the entire analysis on the linear scale.

Step 1: Pre-processing phase

A pop-up window will ask the user to set the thresholds for the maximum percentage of allowed zeros and the minimum percentile for the IQR. Default values are 75 % and 11th percentile, respectively. In this tutorial, both thresholds should be set to the default values.

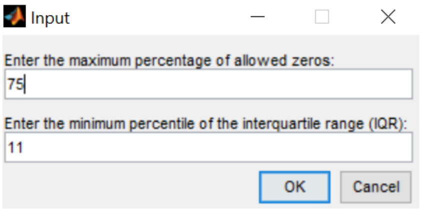

The IQR frequency distribution (Figure UG6) and the scatter plot of the non-zeros values as function of the IQR (Figure UG7) will be produced to visualize the effects of the chosen thresholds.

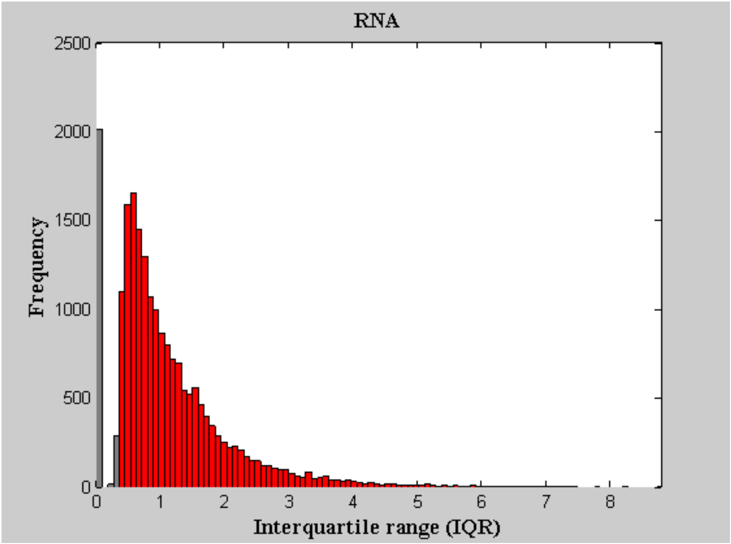

Figure UG6. IQR frequency distribution for brca RNA-sequencing data. The x-axis represents the IQR, while the y-axis represents the frequency of the protein-coding and non-coding RNAs with that IQR range. The grey bars represent the RNAs discarded by applying the selected threshold on IQR.

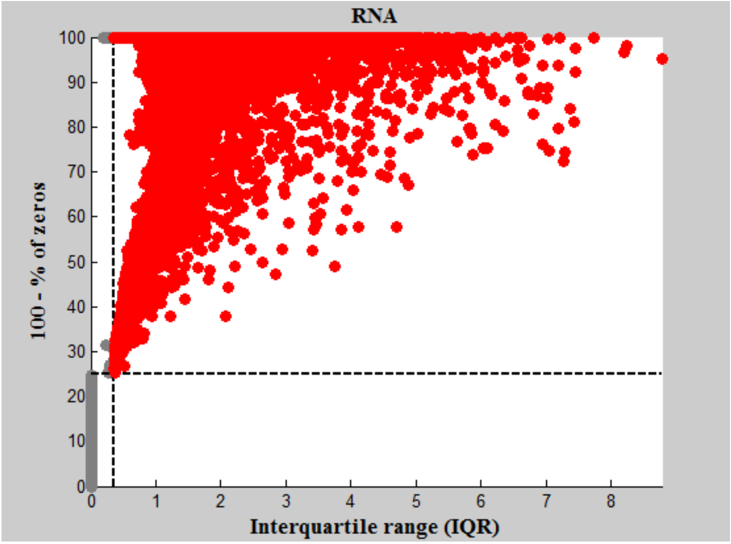

Figure UG7. Scatter plot of the non-zeros values as function of the IQR for brca RNA-sequencing data. The x-axis represents IQR, while the y-axis represents the percentage of non-zero values. The vertical and horizontal dashed lines mark the chosen thresholds. The grey circles represent the RNAs discarded by applying the selected thresholds.

A pop-up window will ask the user to set the thresholds for the maximum percentage of allowed zeros and the minimum percentile for the IQR, for miRNAs. Default values are 75 % and 53th percentile, respectively. In this tutorial, both values should be set to the default values.

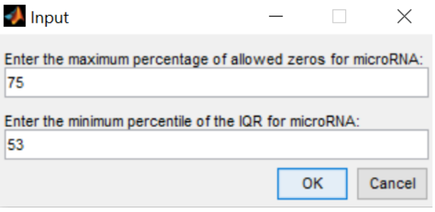

The IQR frequency distribution (Figure UG8) and the scatter plot of the non-zeros values as function of the IQR (Figure UG9) will be produced to visualize the effects of the chosen thresholds for miRNAs.

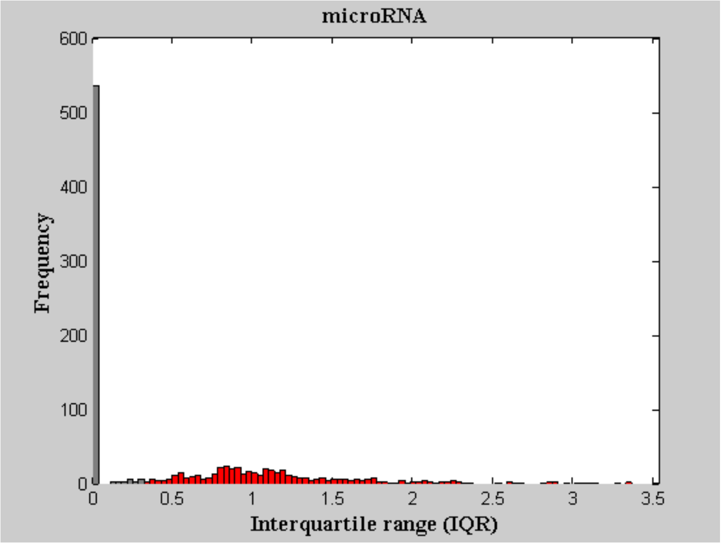

Figure UG8. IQR frequency distribution for brca miRNA-sequencing data. The x-axis represents the IQR, while the y-axis represents the frequency of the miRNAs with that IQR range. The grey bars represent the miRNAs discarded by applying the selected threshold on IQR.

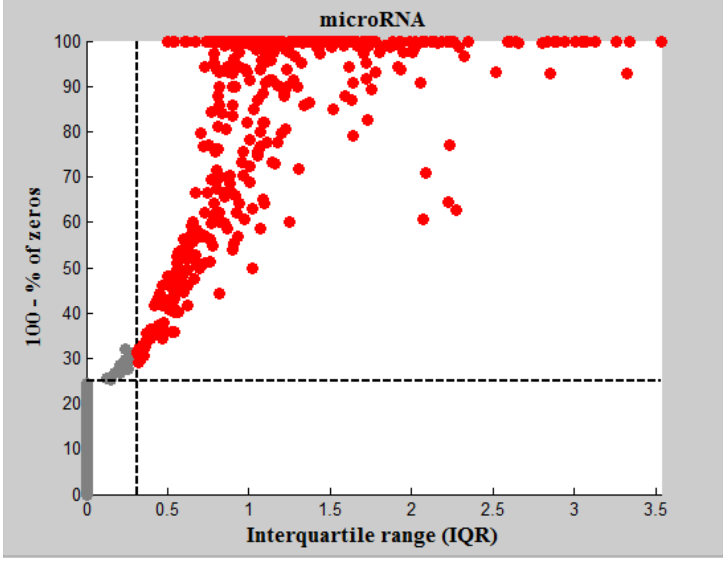

Figure UG9. Scatter plot of the non-zeros values as function of the IQR for brca miRNA-sequencing data. The x-axis represents IQR, while the y-axis represents the percentage of non-zero values. The vertical and horizontal dashed lines mark the chosen thresholds. The grey circles represent the miRNAs discarded by applying the selected thresholds.

A pop-up window will ask the user to confirm or change the currently selected thresholds. In case of acceptance, `SWIM` will proceed to the next steps. In case of rejection, `SWIM` will re-enter the pre-processing phase and the user will have the option to change the threshold values and re-evaluate the related plots until satisfied. In this tutorial choose YES.

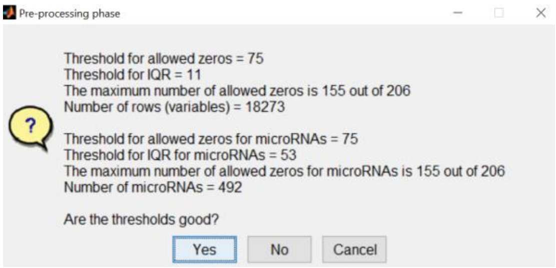

Step 2: Filtering phase

A pop-up window will prompt the user to set the thresholds, for both protein-coding and non-coding RNAs and miRNAs, for the fold-change (linear scale) and for the FDR. Default values are 2 (linear scale) and 0.05, respectively. In this tutorial, fold-change should be set to 3.4 and the FDR should be set to the default value.

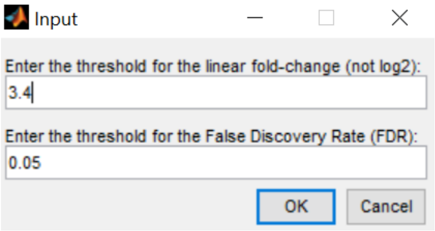

The Volcano plot and the histogram of the fold-change distribution (logarithmic values) for protein-coding and non-coding RNAs (Figure UG10-UG11) and for miRNAs (Figure UG12-13) will be produced to visualize the effects of the chosen thresholds.

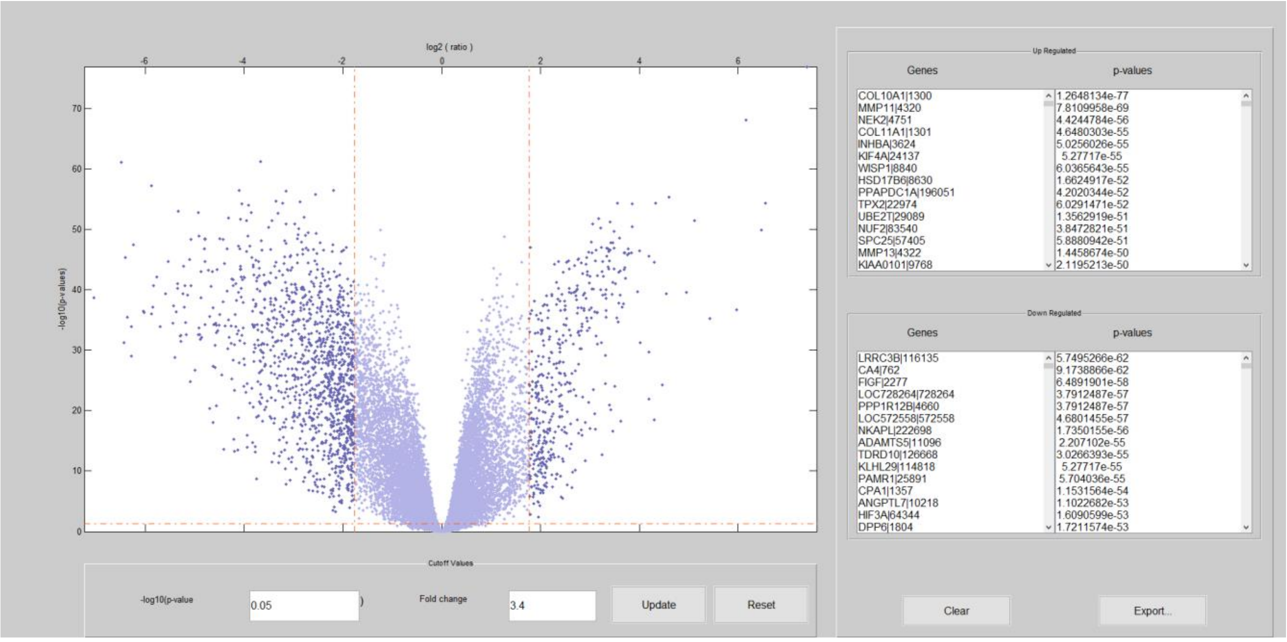

Figure UG10. Volcano plot for brca RNA-sequencing data. The x-axis represents the fold-change ratio (log_2 of the fold-change) that is the ratio between the average tumor expression value and the average normal expression value. The y-axis represents the FDR corrected p-values ($-log_{10}$ of the p-values) of the Student's t-test. The vertical dashed red lines and the horizontal ones correspond to the selected thresholds for the fold-change and the p-values, respectively. The light blue points represent the RNAs that will be discarded according to the selected threshold.

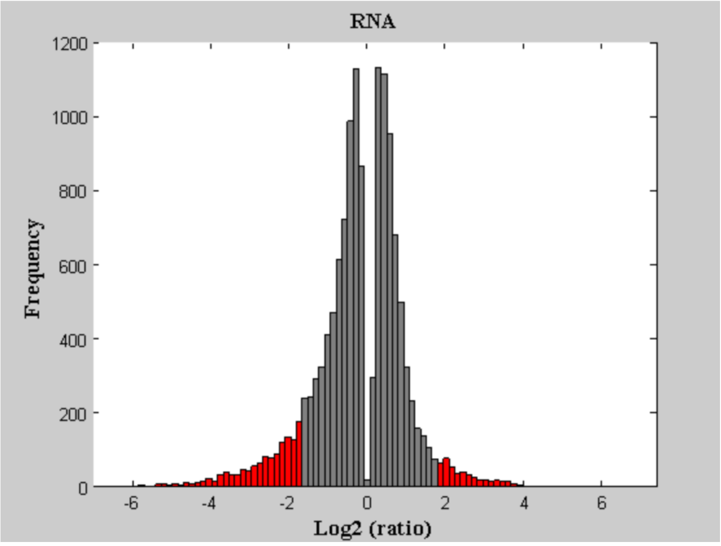

Figure UG11. Distribution of the fold-change values for brca RNA-sequencing data. The x-axis represents the fold-change value (log_2 of the fold-change) that is the ratio of the average tumor expression data to the average normal expression data computed for protein-coding and non-coding RNAs. The y-axis represents the frequency of the obtained fold-change values. The grey bars represent the fold-change values associated with protein-coding and non-coding RNAs that will be discarded according to the selected threshold.

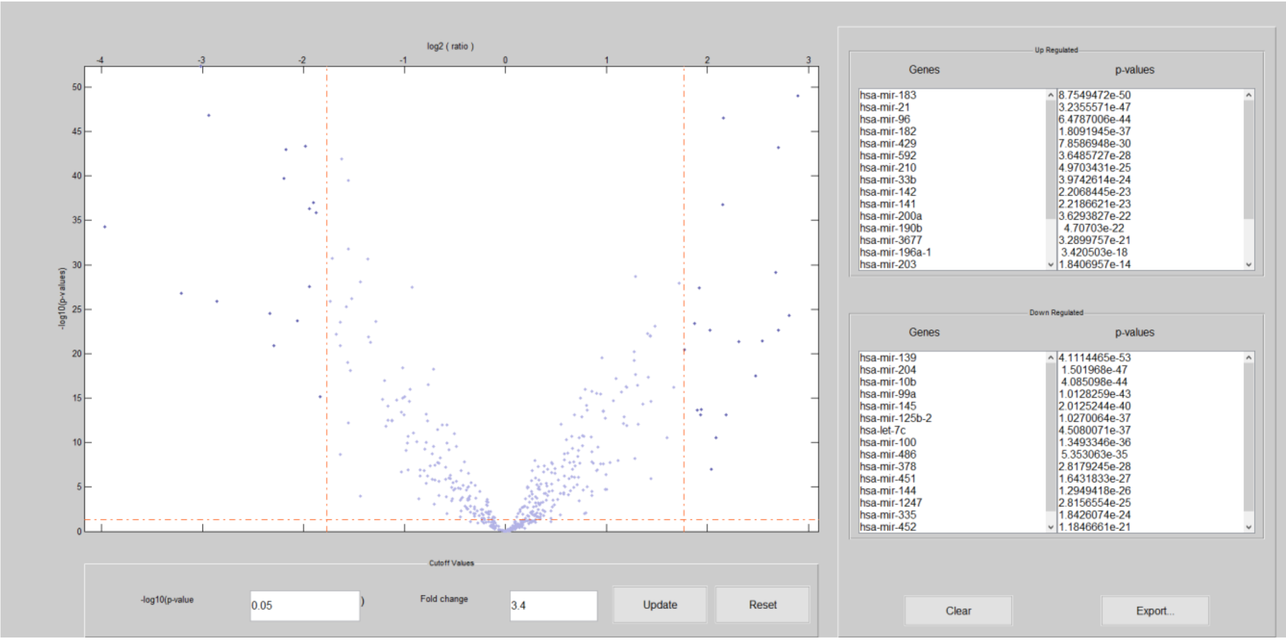

Figure UG12. Volcano plot for brca miRNA-sequencing data. The x-axis represents the fold-change ratio (log_2 of the fold-change) that is the ratio between the average tumor expression value and the average normal expression value. The y-axis represents the FDR corrected p-values ($-log_{10}$ of the p-values) of the Student's t-test. The vertical dashed red lines and the horizontal ones correspond to the selected thresholds for the fold-change and the p-values, respectively. The light blue points represent the miRNAs that will be discarded according to the selected threshold.

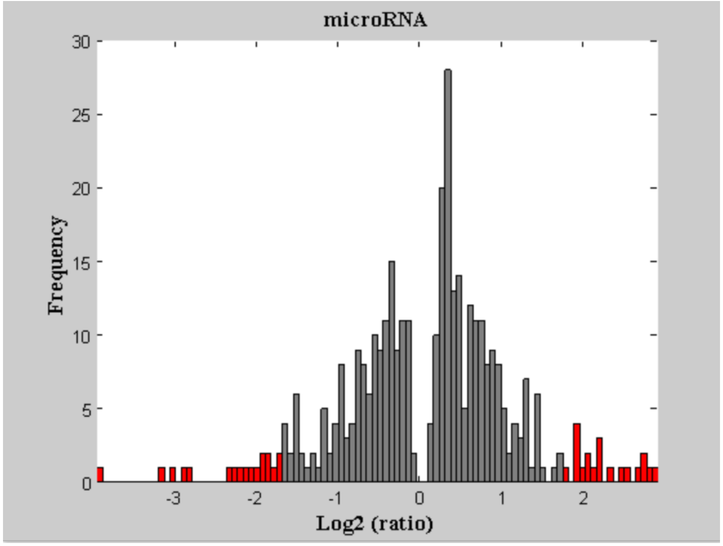

Figure UG13. Distribution of the fold-change values for brca miRNA-sequencing data. The x-axis represents the fold-change value (log_2 of the fold-change) that is the ratio of the average tumor expression data to the average normal expression data computed for miRNAs. The y-axis represents the frequency of the obtained fold-change values. The grey bars represent the fold-change values associated with miRNAs that will be discarded according to the selected threshold.

A pop-up window will ask the user to confirm or change the currently selected thresholds. In case of acceptance, `SWIM` will proceed to the next steps. In case of rejection, `SWIM` will re-enter the filtering processing phase and the user will have the option to change the threshold values and re-evaluate the related plots until satisfied. In this tutorial choose YES.

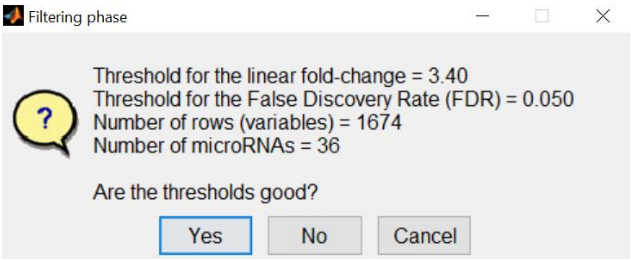

Then, `SWIM` computes hierarchical clustering for rows and columns for both RNA-sequencing and miRNA-sequencing data matrices by using the Pearson correlation as distance metric and the complete-linkage as clustering method. Then, the dendrogram and the heat map will be displayed for both protein-coding and non-coding RNAs (Figure UG14) and miRNAs (Figure UG15).

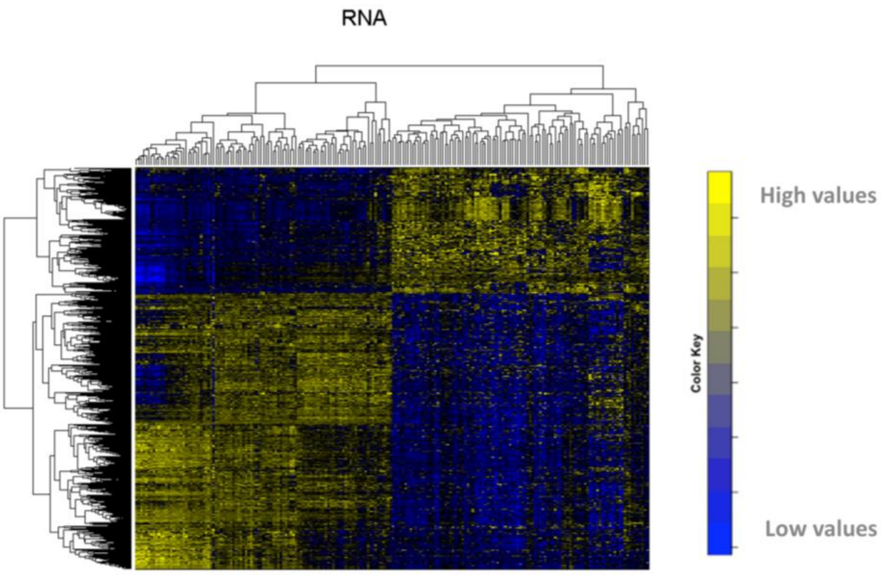

Figure UG14. Dendrogram and heat map for brca RNA-sequencing data. The differential expressed protein-coding and non-coding RNAs are clustered according to rows (genes) and columns (samples) of the RNA-sequencing data matrix (biclustering). The colors represent different expression levels that increase from blue to yellow.

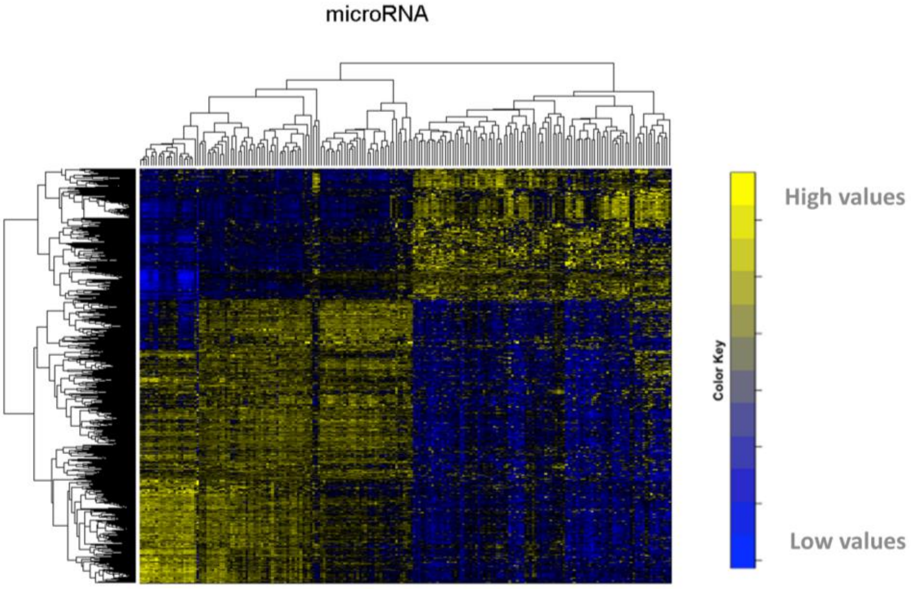

Figure UG15. Dendrogram and heat map for brca miRNA-sequencing data. The differential expressed miRNAs are clustered according to rows (miRNAs) and columns (samples) of the miRNA-sequencing data matrix (biclustering). The colors represent different expression levels that increase from blue to yellow.

Step 3: Building the correlation network

A pop-up window will ask the user to set the threshold for the Pearson correlation coefficient in order to build the correlation network where nodes are genes (including protein-coding and non-coding RNAs, and miRNAs) and a link occurs between two nodes if the absolute value of the Pearson correlation between their expression profiles is greater than the selected threshold. The default value is equal to the 85th percentile of the correlation values distribution. In this tutorial, the threshold should be set to the 80th percentile.

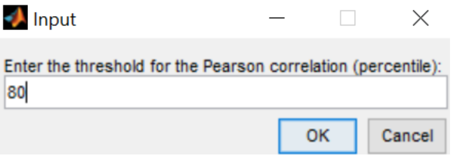

The histogram of Pearson correlation coefficient distribution (Figure UG16) will be produced to visualize the effects of the chosen threshold.

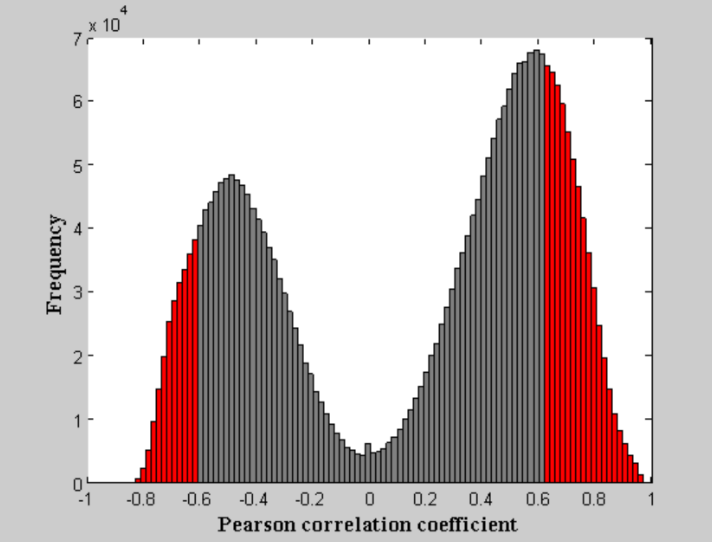

Figure UG16. Distribution of Pearson correlation coefficients for brca RNA-sequencing and miRNA-sequencing data. The x-axis represents the Person correlation coefficient between the expression profiles of all pairs of genes (including protein-coding and non-coding RNAs and miRNAs), while the y-axis represents the frequency. Red (grey) regions correspond to the selected highly (poorly) correlated pairs. Only highly correlated pairs (red regions) will be used to build the correlation network.

A pop-up window will ask the user to confirm or change the choice. In case of rejection, `SWIM` will re-enter in the step 3 and the user will have the option to change the threshold values and re-evaluate the related plots until satisfied. In this tutorial choose YES.

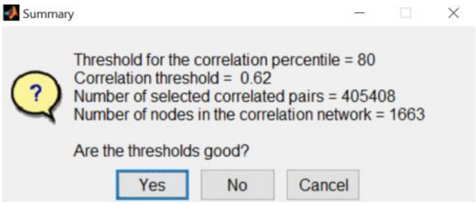

Step 4: Finding communities in the network

A pop-up window will ask the user to set the maximum number of iterations[6] allowed for each replicate of k-means, the number of replicates for a given number of clusters, and the maximum number of clusters for the scree plot. Default values are 100, 5, and 10, respectively. In this tutorial, both numbers should be set to the default values.

---

[6] The maximum number of iterations should be large enough to guarantee convergence. The default value 100 is thus a reasonable choice. If the k-means algorithm fails to converge during a replicate, an orange warning message appears in the Command Window. If many warning messages appear, we strongly suggest restarting `SWIM` increasing the maximum number of iterations.

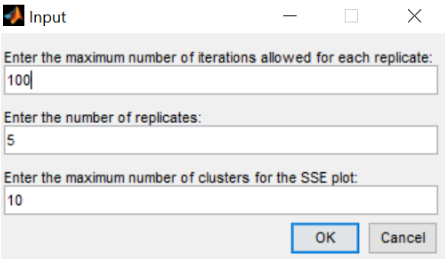

The outputs of the k-means are displayed in the Command Window of MATLAB®. In particular, for each number of clusters and for each replicate, the number of iterations needed to reach the convergence is shown together with the corresponding minimum SSE (i.e., the total sum of distances). Then, the value of the SSE corresponding to the best clusters configuration among all replicates for that number of clusters is displayed (i.e., the best total sum of distances).

```
Number of clusters = 1
Replicate 1, 2 iterations, total sum of distances = 498400.
Replicate 2, 2 iterations, total sum of distances = 498400.
Replicate 3, 2 iterations, total sum of distances = 498400.
Replicate 4, 2 iterations, total sum of distances = 498400.
Replicate 5, 2 iterations, total sum of distances = 498400.
Best total sum of distances = 498400
Number of clusters = 2
Replicate 1, 5 iterations, total sum of distances = 365190.
Replicate 2, 5 iterations, total sum of distances = 328433.
Replicate 3, 3 iterations, total sum of distances = 365190.
Replicate 4, 8 iterations, total sum of distances = 368428.
Replicate 5, 3 iterations, total sum of distances = 365190.
Best total sum of distances = 328433
Number of clusters = 3
Replicate 1, 6 iterations, total sum of distances = 201027.
Replicate 2, 5 iterations, total sum of distances = 201027.
Replicate 3, 20 iterations, total sum of distances = 201027.
Replicate 4, 7 iterations, total sum of distances = 201027.
Replicate 5, 6 iterations, total sum of distances = 201027.
Best total sum of distances = 201027
                         […]
Number of clusters = 10
Replicate 1, 28 iterations, total sum of distances = 153183.
Replicate 2, 25 iterations, total sum of distances = 153757.
Replicate 3, 28 iterations, total sum of distances = 153840.
Replicate 4, 22 iterations, total sum of distances = 154802.
Replicate 5, 32 iterations, total sum of distances = 154064.
Best total sum of distances = 153183
```

The scree plot (Figure UG17) will be produced to guide in the choice for the optimal number of clusters.

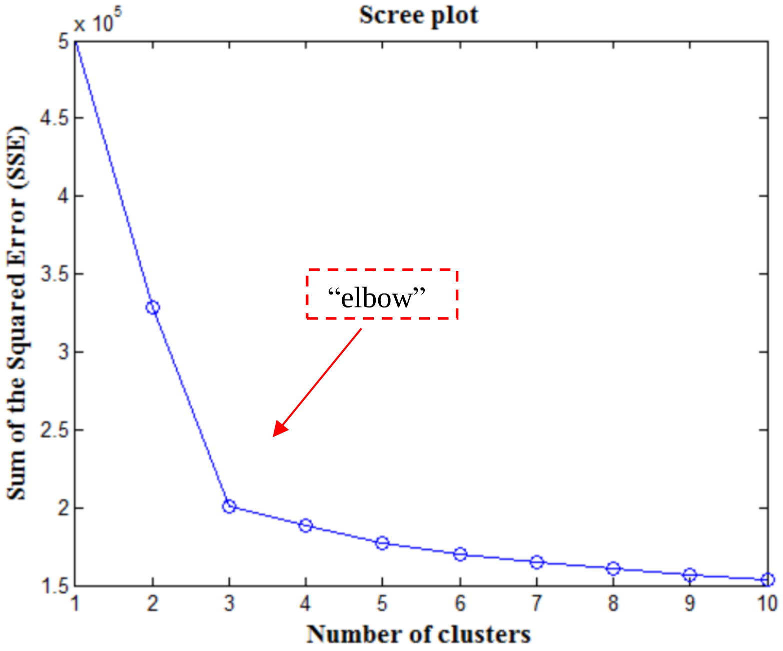

Figure UG17. Scree plot. The x-axis represents the number of clusters, while the y-axis represents the sum of the squared error.

A pop-up window will ask the user to set the maximum number of iterations allowed for each replicate, the number of replicates for the chosen number of clusters, and the chosen number of clusters. In this tutorial, the number of clusters should be set to 3, while the other numbers should be set to the default values.

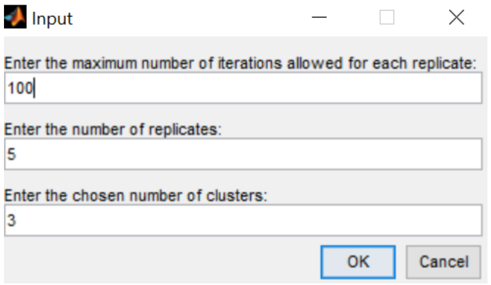


```
Number of clusters = 3
Replicate 1, 6 iterations, total sum of distances = 201027.
Replicate 2, 4 iterations, total sum of distances = 201027.
Replicate 3, 25 iterations, total sum of distances = 352792.
Replicate 4, 9 iterations, total sum of distances = 201027.
Replicate 5, 6 iterations, total sum of distances = 201027.
Best total sum of distances = 201027
```

Step 5: Building the heat cartography map

The heat cartography map is computed (Figure UG18) by coloring each node of the correlation network according to its APCC values.

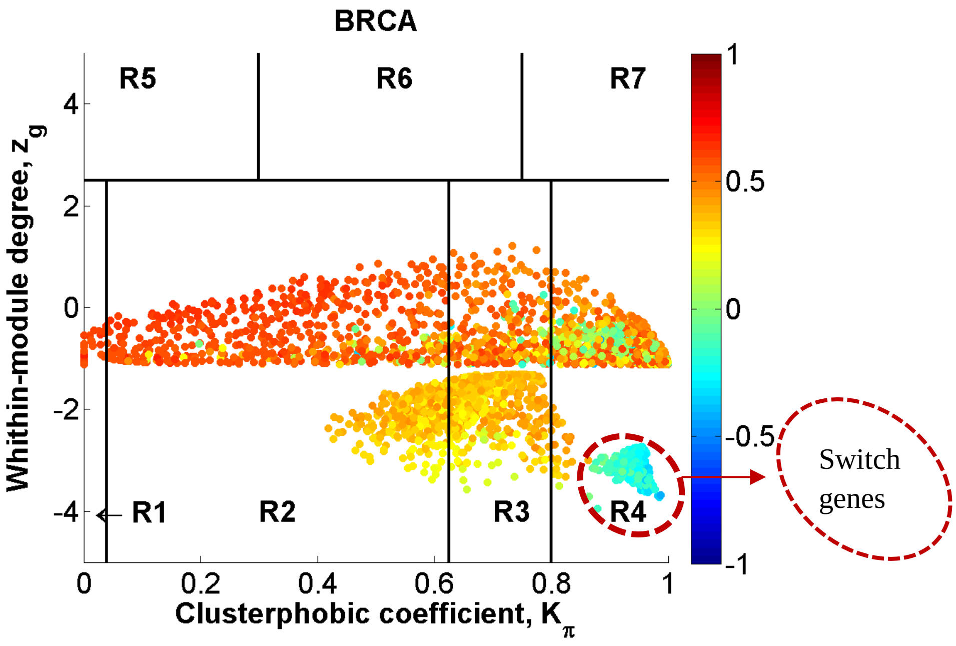

Figure UG18. Heat cartography map for brca correlation network. The plane is identified by two parameters: z_g (within-module degree) and K_π (clusterphobic coefficient) and it is divided into seven regions each defining a specific node role (R1-R7). High z_g values correspond to nodes that are hubs within their module (local hubs), whereas low z_g values correspond to nodes with few connections within their module (non-hubs within their communities, but they could be hubs in the network). Each node is colored according to its APCC value. Specifically, blue nodes are the fight-club hubs (i.e., showing an average negative correlation in expression with their interaction partners), and among them the ones falling in the region R4 are the switch genes. They include miRNAs as well as protein-coding and non-coding RNAs. Being characterized by low z_g and by high K_π values, switch genes are connected mainly outside their module.

Step 6: Identification of switch genes

A pop-up window will ask the user to confirm or change the selection about the optimal number of clusters desired to partition the network. In case of acceptance, `SWIM` will proceed to the next steps. In case of rejection, `SWIM` will ask again the number of clusters and re-enter in the step 5. The user will have the option to change the number of clusters until satisfied. In this tutorial choose YES.

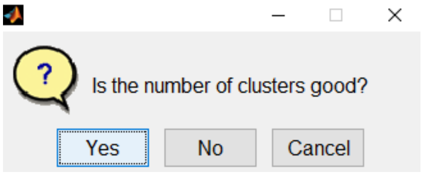

In this tutorial, choose YES and switch genes are finally identified. They are highlighted with the red dashed circle in Figure UG18.

Then, the distribution of the APCC (Figure UG19) is computed. It appears to be trimodal and the three peaks correspond to three different kind of hubs: 1) party hubs which are highly correlated in expression with their interaction partners; 2) date hubs which show moderate correlation in expression with their interaction partners; 3) fight-club hubs which show an average negative correlation in expression with their interaction partners. In order to optimally separate date and party hubs, a date/party threshold is arbitrarily set to 0.5 for the APCC [Han et al., 2004].

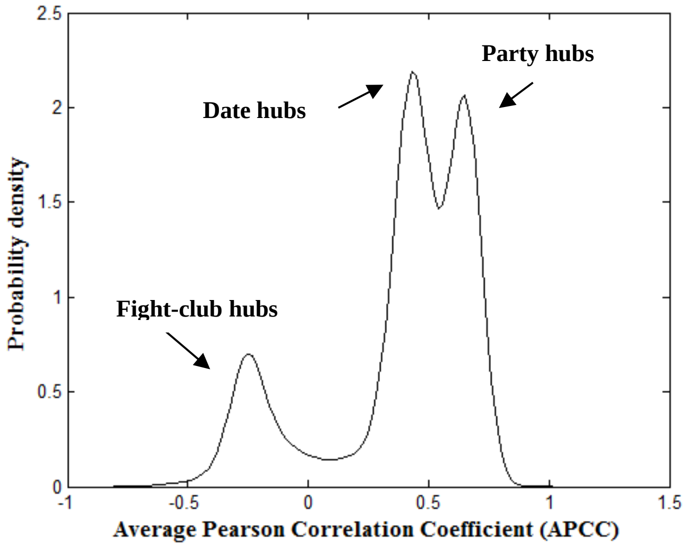

Figure UG19. APCC distribution for brca correlation network. The curve represents the estimated probability density using a smoothing algorithm with a Gaussian kernel of the APCC for each hub of the brca correlation network. The APCC distribution appears to be trimodal and the three peaks correspond to (from right to left): i. party hubs, which are highly correlated with the expression of their interaction partners; ii. date hubs which show moderate co-expression with their interaction partners; iii. fight-club hubs which show average negative correlation with their interaction partners. The x-axis represents the APCC and the y-axis represents the probability density.

Then, `SWIM` computes hierarchical clustering for differentially expressed switch genes by using the Pearson correlation as distance metric and the complete-linkage as clustering method. Then, the dendrogram and the heat map will be displayed (Figure UG20).

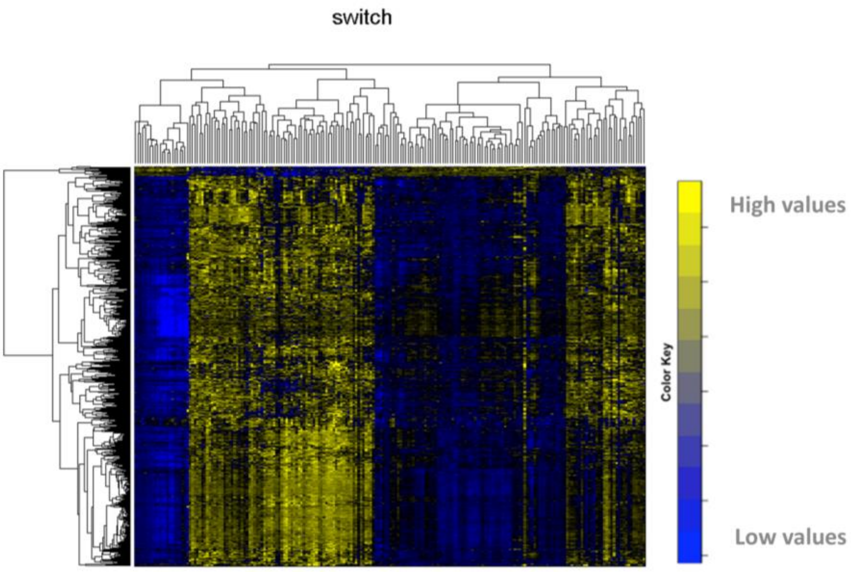

Figure UG20. Dendrogram and heat map for switch genes in brca. The expression profiles of switch genes (including miRNAs, protein-coding and non-coding RNAs) are clustered according to rows (switch genes) and columns (samples) of the switch genes expression data (biclustering). The colors represent different expression levels that increase from blue to yellow.

In the Command Window, a summary of all the chosen thresholds and the number of the identified switch genes will be reported.

```
------------------------------------------------------
                    Summary
------------------------------------------------------
Threshold for allowed zeros = 75
Threshold for IQR = 11
Threshold for allowed zeros for microRNAs = 75
Threshold for IQR for microRNAs = 53
Condition A = list_RNASeq_Tumor
Condition B = list_RNASeq_Normal
Fold-change is list_RNASeq_Tumor/list_RNASeq_Normal
Threshold for the linear fold-change = 3.40
Threshold for the False Discovery Rate (FDR) = 0.050
Threshold for the correlation percentile = 80
Correlation threshold =  0.62

There are 257 switch genes out of 1650 nodes
------------------------------------------------------
```

At this point, the user can choose either to terminate the run or to proceed with further analyses regarding the evaluation of connectivity and robustness of the correlation network.

A pop-up window will ask the user if he/she wishes to evaluate the effect on the network connectivity of varying the Pearson correlation threshold.

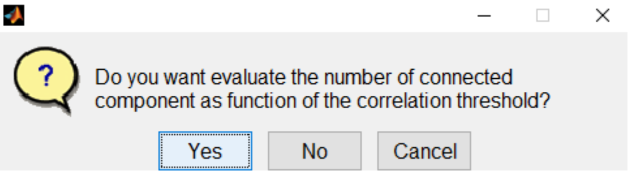

Choosing NO will terminate the run.

Choosing YES, a pop-up window will ask the user to choose the variation range of the Pearson correlation threshold. The default values: minimum=0.1, maximum=0.9, step=0.05. In this tutorial, the variation range should be set to the default values.

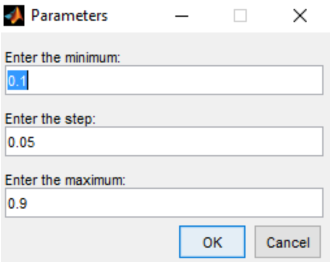

The plot of fraction of nodes populating the largest connected component of the network varying the correlation thresholds will be produced (Figure UG21). Here, the selected correlation threshold is highlighted in red (dashed line). This allows the user to evaluate the effect of his/her choice on the network connectivity. The value of threshold should be the largest one (thus to minimize the number of edges) that maintains the network integrity.

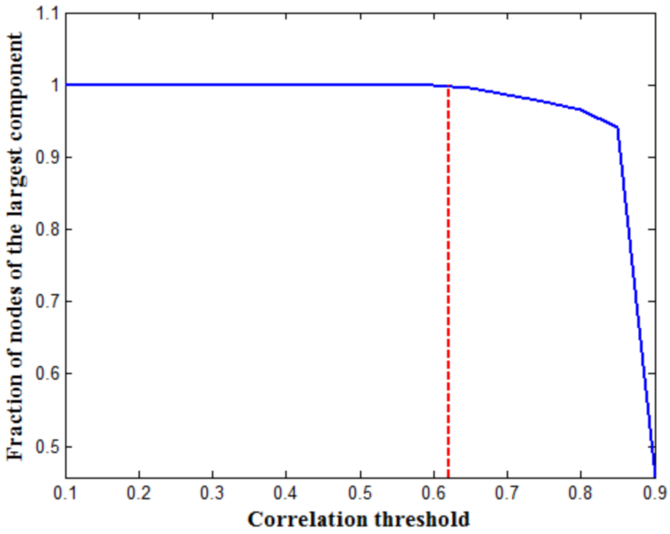

Figure UG21. Connectivity of the brca correlation network. The x-axis represents the Pearson correlation threshold varying in the chosen range, while the y-axis represents the fraction of nodes populating the largest component. The dashed red lines correspond to the selected threshold. Note that y=1 means that all nodes fall in the largest component and thus the network is fully connected; otherwise more components exist. A reasonable choice for the correlation threshold should be the largest one for which the fraction of nodes of the largest connected component is equal to 1.

A pop-up window will ask the user to choose if he/she wants to evaluate the network robustness (i.e., the resilience to errors) with a warning message alerting the user that this step could take a lot of computer time. This is performed by studying the effect on the network connectivity of removing different types of nodes by decreasing degree. The total number of nodes to be removed must be equal to the total number *T* of switch genes and the cumulative node deletion is carried out by type (i.e., total hubs, party hubs, date hubs, fight-club hubs, switch genes, and randomly chosen nodes).

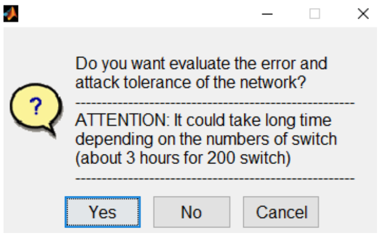

Choosing NO, will exit the run. In this tutorial choose YES.

The plot of the average shortest path as function of the fraction of removed nodes (Figure UG22) will be produced.

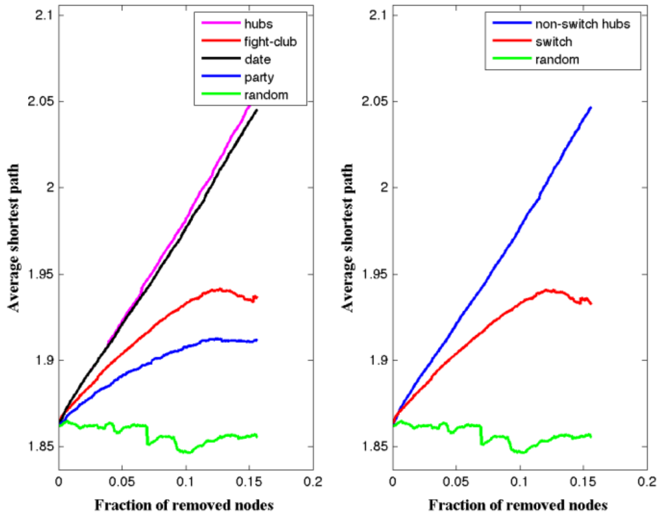

Figure UG22. Robustness for the brca correlation network. The x-axis represents the cumulative fraction of removed nodes, while the y-axis represents the average shortest path. The shortest path between two nodes is the minimum number of consecutive edges connecting them. Each curve corresponds to the variation of the average shortest path of the brca correlation network as function of the removal of nodes specified by the colors of each curve.

Output files

Here, a detailed description of the output files obtained by running `SWIM` on the *sample_project* is provided.

Architecture of the output files

The folders called *all-switch*, *filtering*, and *switch* are created by `SWIM`. The hierarchical structure of the output files and directories is drawn in Figure UG23.

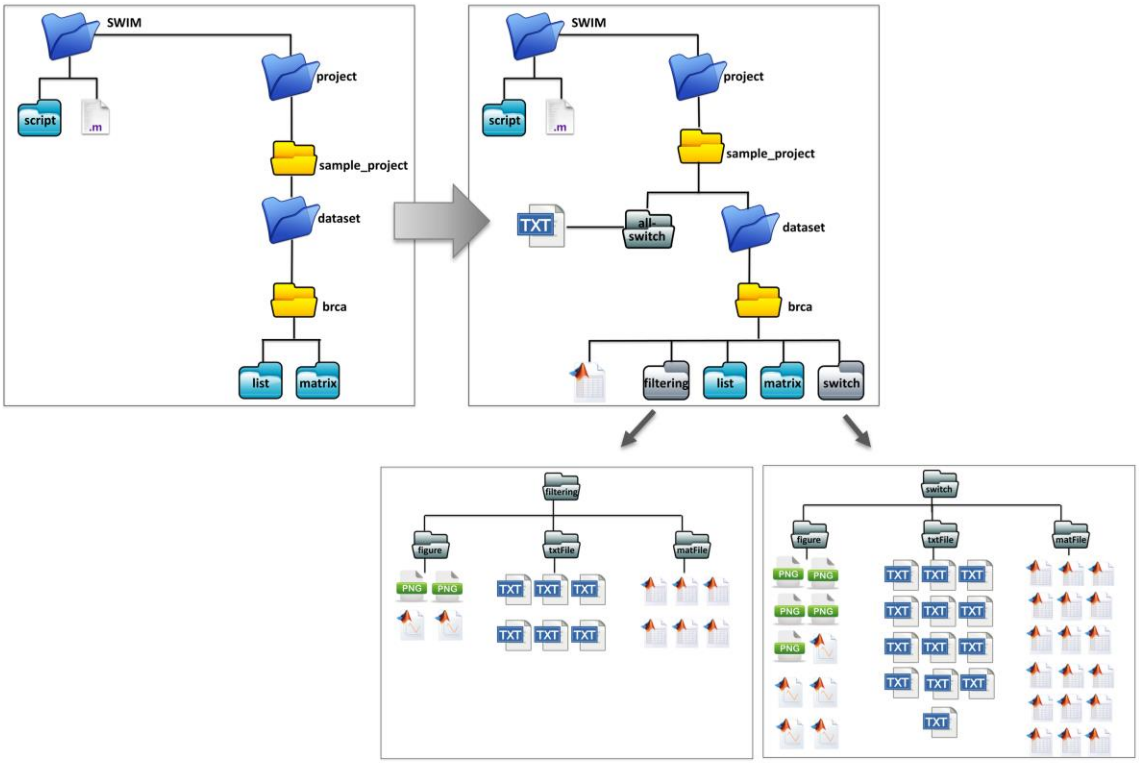

Figure UG23. Architecture and contents of the output files and directories created by `SWIM`. The directories (grey folders) created by `SWIM` for brca dataset are shown together with their contents. The exact number of files (PNG, MAT, FIG, TXT) of each created directory is shown.

all-switch folder

It contains the *brca.txt* file which holds the list of the switch genes identified by `SWIM` in the analysis of the brca dataset. Switch genes are reported with their official gene symbols for protein-coding and non-coding RNAs and their official identifiers for miRNAs.

filtering folder

1. *Figure* folder contains:
   - *DE.png* and *DE.fig*: they are different figure formats of the dendrogram and the heat map depicted in Figure UG14
   - *DE-microRNA.png* and *DE-microRNA.fig*: they are different figure formats of the dendrogram and the heat map depicted in Figure UG15
2. *txtFile* folder contains:
   - *log-dataFiltered.txt*: it includes the preprocessed and the filtered data from the RNA-sequencing data matrix with the logarithmic transformation
   - *stat-log-dataFiltered.txt*: it includes the statistics computed on the RNA-sequencing data with logarithmic transformation
   - *stat-dataORIG.txt*: it includes the statistics computed on the original RNA-sequencing data without logarithmic transformation

   The columns in the above files contains:

   1) *gene*: the names of the genes (TCGA gene identifiers)
   2) *pvalues*: p-values of the statistical hypothesis Student's t-test
   3) *fdr*: FDR correction of the p-values
   4) *mean cancer:* mean of the gene expression values over the cancer tissues
   5) *mean normal:* mean of the gene expression values over the normal tissues

   - *log-dataFiltered-microRNA.txt*: it includes the pre-processed and filtered data from the miRNA-sequencing data matrix with the logarithmic transformation
   - *stat-log-dataFiltered-microRNA.txt*: it includes the statistics computed on the miRNA-sequencing data with logarithmic transformation
   - *stat-dataORIG-microRNA.txt*: it includes the statistics computed on the original miRNA-sequencing data without logarithmic transformation

   The columns of the above files contains:

   1) *gene*: the names of the miRNAs (TCGA miRNA identifiers)
   2) *pvalues:* p-values of the statistical hypothesis Student's t-test
   3) *fdr*: FDR correction of the p-values
   4) *mean cancer:* mean of the gene expression values over the cancer tissues
   5) *mean normal:* mean of the gene expression values over the normal tissues

3. *matFile* folder contains the same file of *txtFile* folder in MATLAB® binary format

***switch* folder**

1. *figure* folder contains:

   - *GA.png* and *GA.fig*: they are different figure formats of the heat cartography map depicted in Figure UG18
   - *APCC-distribution.png* and *APCC-distribution.fig*: they are different figure formats of the APCC distribution depicted in Figure UG19
   - *heatmap_switch.png* and *heatmap_switch.fig*: they are different figure formats of the dendrogram and the heat map depicted in Figure UG20
   - *connectedComponent.png* and *connectedComponent.fig*: they are different figure formats of the plot depicted in Figure UG21
   - *removalNodes.png* and *removalNodes.fig*: they are different figure formats of the plot depicted in Figure UG22

2. *txtFile* folder contains:

   - *CorrelationNetwork.txt*: it contains the correlation network with the following columns:
     1) *Source*: source node of the correlation network (TCGA gene or miRNA identifiers)
     2) *Target*: target node of the correlation network (TCGA gene or miRNA identifiers)
     3) *Correlation:* Pearson correlation coefficient between source and target nodes
   - *idx.txt*: it contains for each node of the correlation network the name and its belonging cluster
   - *CartographyNetwork.txt*: it contains the nodes appearing in the heat cartography map, with the following columns:
     1) *Source*: source node of the heat cartography map (TCGA gene or miRNA identifiers)
     2) *Target*: target node of the heat cartography map (TCGA gene or miRNA identifiers)
     3) *Correlation:* Pearson correlation coefficient between source and target nodes

   Note that in general not all nodes of the correlation network appear in the heat cartography.

   - *attribute.txt*: it reports the features of all nodes in the heat cartography map. The columns of the file are the following ones:
     1) *nodeName*: the names of the nodes in the heat cartography map (TCGA gene or miRNA identifiers)
     2) *Hub*: it specifies if the nodes is (or is not) a local hub within their community
     3) *Region*: it specifies the region of the heat cartography to which the node belongs and corresponds to its universal role in the network
     4) *Type*: it specifies for each node its universal role
     5) *Degree*: it specifies the node degree (i.e., the number of incoming and outgoing edges of each node)
     6) *APCC*: it specifies the average of the Pearson correlation coefficients between the expression profiles of a node and those of its interaction partners
     7) *Date-Party*: it specifies if the node is either a date or party or fight-club hub or if it is not a local hub in its community
     8) K_π: it specifies the value of the *clusterphobic coefficient* parameter
     9) z_g: it specifies the value of the *within-module degree* parameter

- *attribute-switch.txt*: it reports only the attributes of the switch genes as discussed above (*attribute.txt*)
- *switch.txt*: it contains the names of the switch genes (TCGA gene or miRNA identifiers)
- *fc_switch.txt*: it contains the log-ratio value for each switch genes. The columns are the following ones:
  1) *switch:* the names of the switch genes (TCGA gene or miRNA identifiers)
  2) *log-ratio:* it represents the logarithm of the ratio of the average of cancer expression values to the average of the normal expression values
- *nn_name_neg_switch.txt*: it is a matrix where the first column contains the names of the switch genes and each row contains the names of the nodes that are connected and negatively correlated in expression with the corresponding switch gene
- *nn_name_pos_switch.txt*: it is a matrix where the first column contains the names of the switch genes and each row contains the names of the nodes that are connected and positively correlated in expression with the corresponding switch gene
- *corr_nn_pos_switch.txt*: it contains the expression Pearson correlation coefficients between switch genes (reported in the first column) and their positive correlated linked nodes. The second column of this file corresponds to the average of each row.
- *corr_nn_neg_switch.txt*: it contains the expression Pearson correlation coefficients between switch genes (reported in the first column) and their negative correlated linked nodes. The second column of this file corresponds to the average of each row.
- *cluster_nn_pos_switch.txt*: it contains the belonging clusters of the switch genes (reported in the first column of this file) and their positive correlated linked nodes (rows)
- *cluster_nn_neg_switch.txt*: it contains the belonging clusters of the switch genes (reported in the first column of this file) and their negative correlated linked nodes (rows)

3. *matFile* folder contains:

- *Rho.mat*: it includes two square and symmetric matrices:
  1) *pval*: the p-values of the Pearson correlation
  2) *rho*: the Pearson correlation matrix
- *CorrelationNetwork.mat*: it contains:
  1) *diag_Nr*: the row indexes of the Pearson correlation matrix of the source nodes
  2) *diag_Nc*: the column indexes of the Pearson correlation matrix index of the target nodes
  3) *nodeName*: the names of all the nodes in the correlation network
  4) *net*: the correlation network
- *adjMatrix.mat*: it contains the adjacency matrix of the correlation network, a square and symmetric matrix, whose size is equal to the number of network nodes, and whose elements a_{ij} are equal to 1 if there exists an edge between the i and j node, equal to zero otherwise
- *SSE.mat*: it includes the SSE values as function of the number of clusters corresponding to the y-axis of Figure UG17
- *idx.mat*: it contains the belonging cluster of each node in the correlation network. It corresponds to the second *idx.txt*.

- *connectedComponent.mat*: it includes *thr* and *ratio* vectors corresponding to the x-axis and y-axis of Figure UG21, respectively

- *CartographyNetwork.mat*: it contains:

  1) *nodeName*: the names of the nodes of the heat cartography map
  2) *net*: the correlation network in the cartographic representation (heat cartography map)

  Note that not all nodes of the correlation network appear in the heat cartography.

- *attribute.mat*: it contains twelve cell arrays:

  1) *nodeName*: the names of the nodes of the correlation network (TCGA gene or miRNA identifiers)
  2) *nodeNameG*: the names of the nodes of the heat cartography map. It corresponds to the *nodeName* column of the *attribute.txt* file.
  3) *attribute1*: it specifies if the nodes of the heat cartography map are (or are not) local hubs within their community. It corresponds to the *Hub* column of the *attribute.txt* file.
  4) *attribute2*: it specifies the region of the heat cartography map to which each node belongs. It corresponds to the *Region* column of the *attribute.txt* file.
  5) *attribute3*: it specifies for each node of the heat cartography map its universal role. It corresponds to the *Type* column of the *attribute.txt* file.
  6) *P*: values of the clusterphobic coefficient parameter. It corresponds to the K_π column of the *attribute.txt* file
  7) *z*: values of the within-module degree parameter. It corresponds to the z_g column of the *attribute.txt* file
  8) *APCC*: it includes features of all the nodes in the heat cartography map:

     i. the average of the Pearson correlation coefficients between the expression profiles of a node and those of its interaction partners in the heat cartography map (*APCC* column in the *attribute.txt* file)
     ii. the degree for each node in the heat cartography map (*Degree* column in the *attribute.txt* file)
     iii. if the node of the heat cartography map is either a date or party or fight-club hub or if it is not a local hub in its community (*Date-Party* column in the *attribute.txt* file)

  9) *APCC_all*: the same of *APCC* but for all nodes in the correlation network
  10) *meanAPCC*: first column of the cell array *APCC*
  11) *degG*: total degree of the nodes in the heat cartography map
  12) *deg*: total degree of the nodes in the correlation network

- *attribute-switch.mat*: it is the same as *attribute.mat* but for switch genes. It corresponds to *attribute-switch.txt.*

- *NearestNeigborn.mat*: it includes all the information about clusters and neighbors of each switch gene:

  1) *unique_list_cluster_switch*: unique list of the belonging clusters of the switch genes
  2) *unique_list_cluster_nn_neg_switch*: unique list of the belonging clusters of the nodes that are connected and negatively correlated in expression with the switch genes
  3) *unique_list_cluster_nn_pos_switch*: unique list of the belonging clusters of the nodes that are connected and positively correlated in expression with the switch genes
  4) *unique_list_nn_neg_switch*: unique list of the node names that are connected and negatively correlated in expression with the switch genes

5) *unique_list_nn_pos_switch*: unique list of the node names that are connected and positively correlated in expression with the switch genes
6) *nn_name_neg_switch*: a matrix where the first column contains the names of the switch genes and each row contains the names of the nodes that are connected and negatively correlated in expression with the corresponding switch gene. It corresponds to *nn_name_neg_switch.txt*.
7) *nn_name_pos_switch*: a matrix where the first column contains the names of the switch genes and each row contains the names of the nodes that are connected and positively correlated in expression with the corresponding switch gene. It corresponds to *nn_name_pos_switch.txt*.
8) *sum_nn_neg_switch*: total number of nodes that are connected and negatively correlated in expression with each switch gene. The list is ordered as the first column of matrix *nn_name_neg_switch*.
9) *sum_nn_pos_switch*: total number of nodes that are connected and positively correlated in expression with each switch gene. The list is ordered as the first column of matrix *nn_name_pos_switch*.
10) *switc*: the names of all the switch genes (TCGA gene or miRNA identifiers). It corresponds to *switch.txt*.

- *Switc.mat*: it includes for the switch genes the following summarizing variables:

1) *CpzmRNA*: cancer tissue labels
2) *NpzmRNA*: normal tissue labels
3) *data_switch*: the expression data matrix of the switch genes (rows) in cancer and matched normal tissues (columns)
4) *switc*: the names of all the switch genes (TCGA gene or miRNA identifiers). It corresponds to *switch.txt.*
5) *tissue*: the cancer and matched normal tissues

If the robustness of the network has been evaluated, the following additional files are provided:

- *removalDateHubs.mat*
- *removalFightClub.mat*
- *removalHubs.mat*
- *removalNonSwitch.mat*
- *removalPartyHubs.mat*
- *removalSwitch.mat*
- *removalRandomNodes.mat*

each one including the following three cell arrays:

1) *lambda*: the averaged shortest paths (y-axes of Figure UG22)
2) *f*: the fraction of the removed nodes (x-axes of Figure UG22)
3) *size*: the size of the largest connected component of the network

References

Benjamini Y and Hochberg Y (1995). Controlling the false discovery rate: a practical and powerful approach to multiple testing. Journal of the Royal Statistical Society. Series B (Methodological), 289-300.

Cancer Genome Atlas Research Network, Weinstein JN, Collisson EA, Mills GB, Shaw KR, Ozenberger BA, Ellrott K, Shmulevich I, Sander C, Stuart JM (2013). The Cancer Genome Atlas Pan-Cancer analysis project. Nat Genet. 2013 Oct;45(10):1113-20.

Fasoli M, Dal Santo S, Zenoni S, Tornielli GB, Farina L, et al. (2012) The grapevine expression atlas reveals a deep transcriptome shift driving the entire plant into a maturation program. Plant Cell 24: 3489-505.

Guimera R, Amaral LAN (2005). Cartography of complex networks: modules and universal roles. J Stat Mech. P02001, 1–13.

Guimera R, Amaral LAN (2005). Functional cartography of complex metabolic networks. Nature 433: 895.

Han JD, Bertin N, Hao T, Goldberg DS, Berriz GF, Zhang LV, Dupuy D, Walhout AJ, Cusick ME, Roth FP, Vidal M (2004). Evidence for dynamically organized modularity in the yeast protein-protein interaction network. Nature 430: 88-93.

Hartigan JA (1973). Clustering. Annu Rev Biophys Bioeng 2: 81-101.

Lisboa, PJ, Etchells TA, Jarman IH and Chambers SJ (2013). Finding reproducible cluster partitions for the k-means algorithm. BMC bioinformatics,14(1), 1.

Meila M (2006). The uniqueness of a good optimum for k-means. In Proceedings of the 23rd international conference on Machine learning (pp. 625-632). ACM.

Palumbo, MC, Zenoni S, Fasoli M, Massonnet M, Farina L, Castiglione F, Pezzotti M, and Paci P (2014). Integrated network analysis identifies fight-club nodes as a class of hubs encompassing key putative switch genes that induce major transcriptome reprogramming during grapevine development. The Plant Cell, 26(12), 4617-4635.

Contacts

Paola Paci - paola.paci@iasi.cnr.it
Giulia Fiscon - giulia.fiscon@iasi.cnr.it

How to cite `SWIM`

If you use `SWIM`, please cite the present manuscript.

Glossary

A

APCC - the Average Pearson Correlation Coefficient computed between the expression profile of a node and each of its nearest neighbors [Han et al., 2004]

adjacency matrix - square matrix, whose size is equal to the number of network nodes and whose elements a_{ij} are equal to 1 if there exists an edge between the *i* and *j* node, equal to zero otherwise. In case of undirected networks, the adjacency matrix is symmetric.

B

biclustering - simultaneous clustering of the rows and columns of a matrix

C

centroid (of a cluster) - the average of all the points in a cluster (i.e., the middle point of a cluster)

clustering - grouping a set of *n* objects in *N* groups (clusters) where objects in the same cluster are more similar to each other than to those in other clusters. It aims to find a structure in a collection of unlabelled data.

connected component - a set of nodes in a network that are linked to each other by paths

D

date hub - hub showing moderate correlation with the expression of its interaction partners

degree (of a node) - number of incoming and outgoing edges of a node

dendrogram - tree-structured diagram used to visualize the result of a hierarchical clustering

F

FDR - the False Discovery Rate, which is a procedure to correct significance for multiple testing [Benjamini and Hochberg, 1995]

fight-club hub - hub showing an average negative correlation with the expression of its interaction partners

fold-change - given two conditions A and B, it is the ratio of the average expression of samples in condition A to the average expression of samples in condition B

H

heat map - graphical representation of a data matrix whose values are represented by different degrees of colours intensity according to a defined colours scheme

hierarchical clustering - set of nested clusters organized as a hierarchical tree. Each node (cluster) in the tree is the union of his children (except for the leaves), while the root is the cluster containing all the objects.

hub - node of the network whose degree exceeds the threshold of 5, according to [Han et al., 2004]

I

IQR - the Inter Quartile Range, which is a measure of statistical dispersion, equal to the difference between the third and the first quartiles, corresponding to the 75th and the 25th percentile, respectively. It is the range of the middle 50% of the data.

K

k-means clustering - a clustering algorithm whose aim is to partition a set of *n* objects in *N* groups (clusters) so that each object belongs to the cluster with the nearest centroid. The number *N* of clusters must be chosen before applying the algorithm.

L

linkage - method to determine the distance between two clusters

linkage-complete - the distance between two clusters is determined as the maximum distance between any two nodes (one in each cluster).

log fold-change - the logarithm of the ratio calculated between the average expression of samples in a condition A and the average expression of samples in a condition B

N

network - set of elements (nodes) joined by a set of links (edges)

network connectivity - property of a network measuring how well part of the network connect to one another

P

p-value - the smallest probability for which data allow to reject the null hypothesis of a statistical hypothesis test

party hub - hub showing high correlation with the expression of its interaction partners

path - number of consecutive edges connecting two nodes (all the edges and nodes must be different)

Pearson correlation coefficient - a measure of the linear correlation between two variables, that is the covariance of the two variables divided by the product of their standard deviations.

percentiles - values that divide a rank-ordered set of elements into 100 equal parts

power law distribution (of node degree in a network) - the probability $P(k)$ of nodes in the network having k links to other nodes goes for large values of k as $P(k) \sim k^{-\gamma}$. There are few nodes with high degree (hubs) and many nodes with low degree.

Q

quartiles - values that divide a rank-ordered set of elements into four equal parts

R

robustness - the resilience to errors. In the context of a network, the robustness measures the change in the average shortest path after removal of specific nodes (e.g., randomly selected nodes, nodes sorted by degree).

S

scale-free network - network whose degree distribution follows a power law distribution with parameter $\gamma \in (2,3)$

shortest path - the minimum path connecting two nodes

SSE - the Sum of the Squared Error. In the context of measuring the quality of a clustering, this indicates the total sum over all clusters of the squared distances of each node of a given cluster from its corresponding centroid.

Student's t-test - statistical test evaluating the null hypothesis that the data in vectors *x* and *y* comes from independent random samples from normal distributions with equal means and equal but unknown variances. The alternative hypothesis is that the data in x and y comes from populations with unequal means.

T

tree - data structure in which any two nodes are connected by exactly one path

TCGA - The Cancer Genome Atlas (cancergenome.nih.gov) by the National Cancer Institute and the National Human Genome Research Institute. It is a project providing a comprehensive repository of large-scale genomics data, by collecting high-quality human cancer samples as well as their matching normal tissues. TCGA aims to create a detailed multi-dimensional catalog, or "atlas," of the key genomic changes associated with specific types of tumors to improve the prevention, diagnosis and treatment of cancer.

TCGA barcode - a multi-field, dash-separated, alphanumeric identifier associated with every TCGA bio-specimen data. In particular, the first three fields in the barcode identifies the patient, and the fourth one the type of sample (normal or cancer). Further details on the barcode can be found here: https://wiki.nci.nih.gov/display/TCGA/TCGA+barcode.

SWIM: a computational tool to unveiling crucial nodes in complex biological networks

Paola Paci[1,2,*], Teresa Colombo[1], Giulia Fiscon[1], Aymone Gurtner[3], Giulio Pavesi[4], and Lorenzo Farina[5]

[1]Institute for Systems Analysis and Computer Science "Antonio Ruberti", National Research Council, Rome, Italy
[2]SysBio Centre for Systems Biology, 00185 Rome, Italy
[3]Department of Research, Advanced Diagnostics, and Technological Innovation, Translational Research Area, Regina Elena National Cancer Institute, Rome, Italy
[4]Department of Biosciences, University of Milan, Italy
[5]Department of Computer, Control and Management Engineering, "Sapienza" University, Rome, Italy
[*]paola.paci@iasi.cnr.it

ABSTRACT

SWItchMiner (SWIM) is a wizard-like software implementation of a procedure, previously described, able to extract information contained in complex networks. Specifically, SWIM allows unearthing the existence of a new class of hubs, called "fight-club hubs", characterized by a marked negative correlation with their first nearest neighbors. Among them, a special subset of genes, called "switch genes", appears to be characterized by an unusual pattern of intra- and inter-module connections that confers them a crucial topological role, interestingly mirrored by the evidence of their clinic-biological relevance.
Here, we applied SWIM to a large panel of cancer datasets from The Cancer Genome Atlas, in order to highlight switch genes that could be critically associated with the drastic changes in the physiological state of cells or tissues induced by the cancer development. We discovered that switch genes are found in all cancers we studied and they encompass protein coding genes and non-coding RNAs, recovering many known key cancer players but also many new potential biomarkers not yet characterized in cancer context. Furthermore, SWIM is amenable to detect switch genes in different organisms and cell conditions, with the potential to uncover important players in biologically relevant scenarios, including but not limited to human cancer.

Supplementary File 2 - Supplementary Figures

In the following, the reader will find all the supplementary figures cited in the text and their legends.

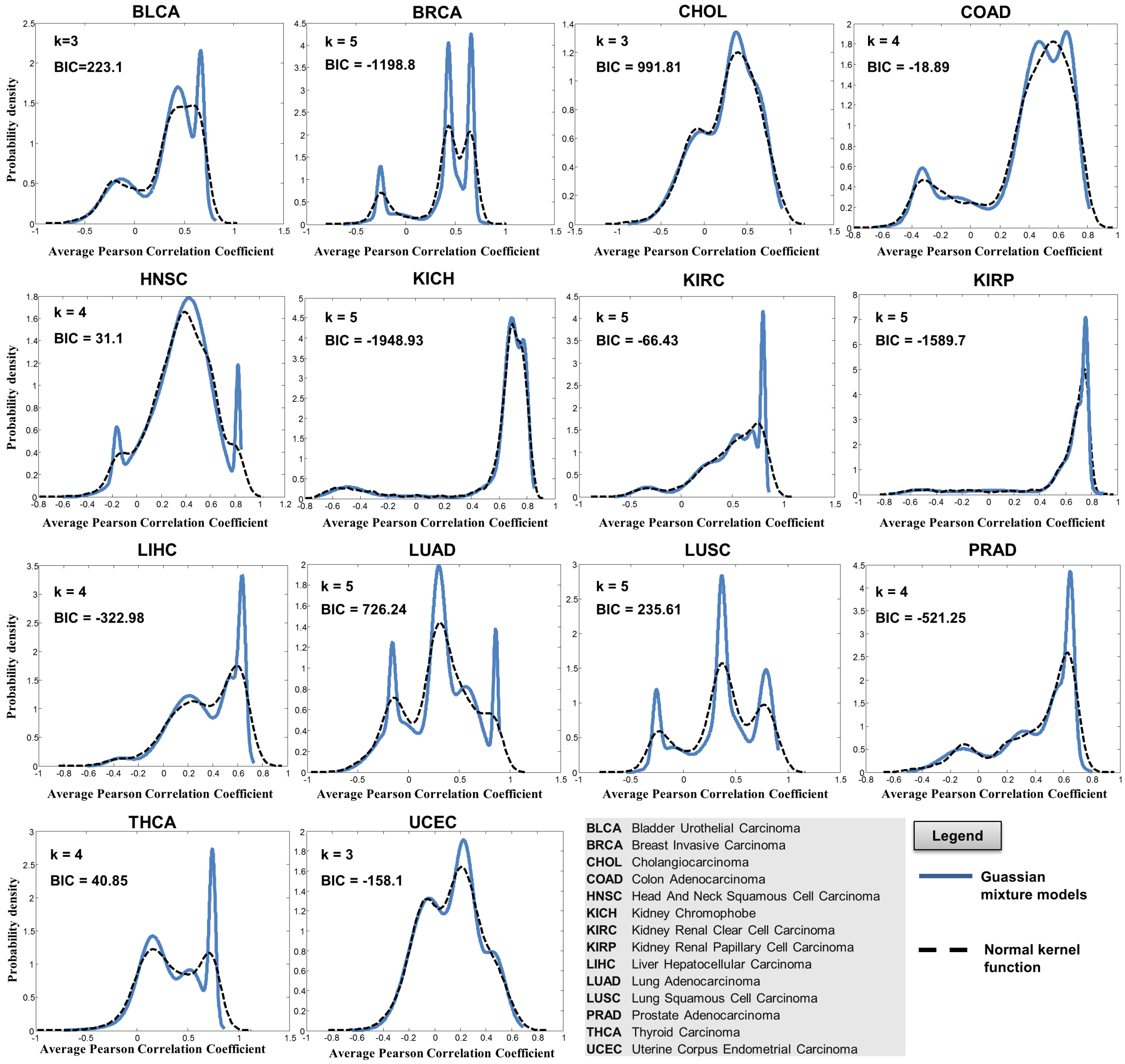

Figure 1. Averaged Pearson Correlation Coefficient in 14 human cancers of TCGA. In each panel the dashed curve is the estimated probability density using a smoothing algorithm with a Gaussian kernel of the APCC for each hub (*i.e.* node with degree greater than 5) of the correlation network obtained using the RNA-sequencing data of expression available on TCGA data portal for the 14 tumor we studied (see legend); while the solid blue line is the corresponding best fitting obtained by using a Gaussian mixture models (GMM) composed of k multivariate normal density components, where k is a positive integer ranging from one to five. The best fit is obtained by using Bayesian information criteria (BIC). In many cases, the distribution of APCCs is trimodal, with two peaks (not always apparent) representing low and high positive APCC values (mirroring the date and party hub distributions in PPI networks) and a third peak (always existing) representing negative APCC values (fight-club hubs). Following[1], an arbitrary date/party threshold value of APCC equal to 0.5 was defined in order to optimally separate the two types of hubs.

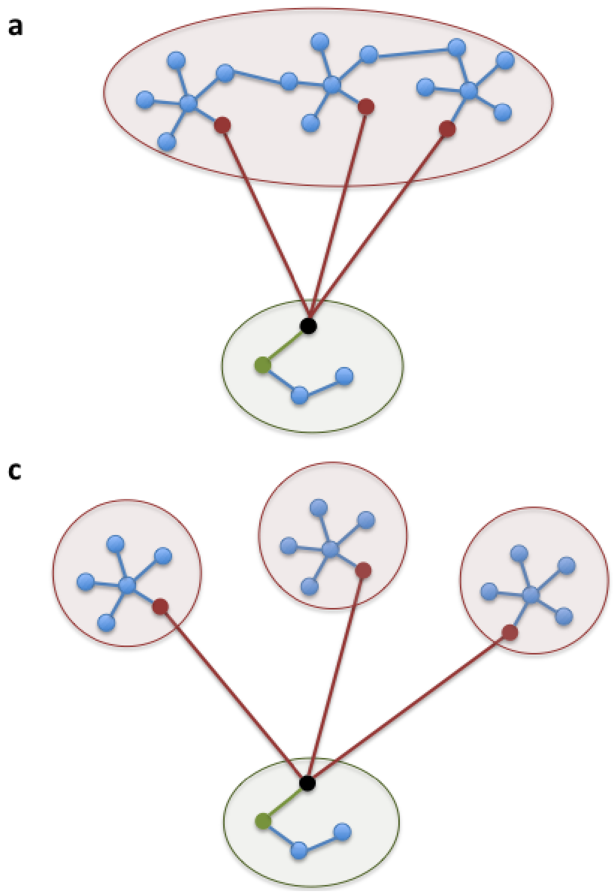

Figure 2. Different definitions of the inter and intramodule connections of a node. Following[2,3], the participation coefficient P given by equation 2 in the main text increases from panel **a** to panel **c** where it approaches to its highest values since it is connected to all communities partitioning the network (including its own) to the same extent: $P_i = 1 - \frac{1}{16} - \frac{9}{16} = \frac{6}{16} = 0.375$ (panel **a**); $P_i = 1 - \frac{1}{16} - \frac{1}{16} - \frac{4}{16} = \frac{10}{16} = 0.625$ (panel **b**); $P_i = 1 - \frac{1}{16} - \frac{1}{16} - \frac{1}{16} = \frac{12}{16} = 0.75$ (panel **c**). On the contrary, P assumes the same values in panels **a** and **d** even if they correspond to completely different neighborhood topologies: $P_i = 1 - \frac{1}{16} - \frac{9}{16} = \frac{6}{16} = 0.375$ (panel **d**). Moreover, in panels **a**, **b**, **c** the black node has 25% of links within its module (1 out of 4) and thus - according to[2,3] - it should fall in R4 region where $P > 0.8$ but P is always less then 0.8 in the situation depicted here.
In our formulation we obtain the same values of the clusterphobic coefficient K_π, defined by equation 4 in the main text, for panels **a**, **b** and **c**, since the ratio of internal to external links doesn't change and reflects the 'fear' of being confined in a cluster. Consistently, the clusterphobic coefficient is higher in panel **a** than panel **d** capturing the substantial difference in the topological role of the black node in these two situations: in panel **a** it acts as a non-hub connector (here 'hub' is used in relation to within-community hubs), while in panel **d** it functions as local hub. $K_\pi^i = 1 - \frac{1}{16} = \frac{15}{16} = 0.94$ (panel **a**); $K_\pi^i = 1 - \frac{9}{16} = \frac{7}{16} = 0.44$ (panel **d**).

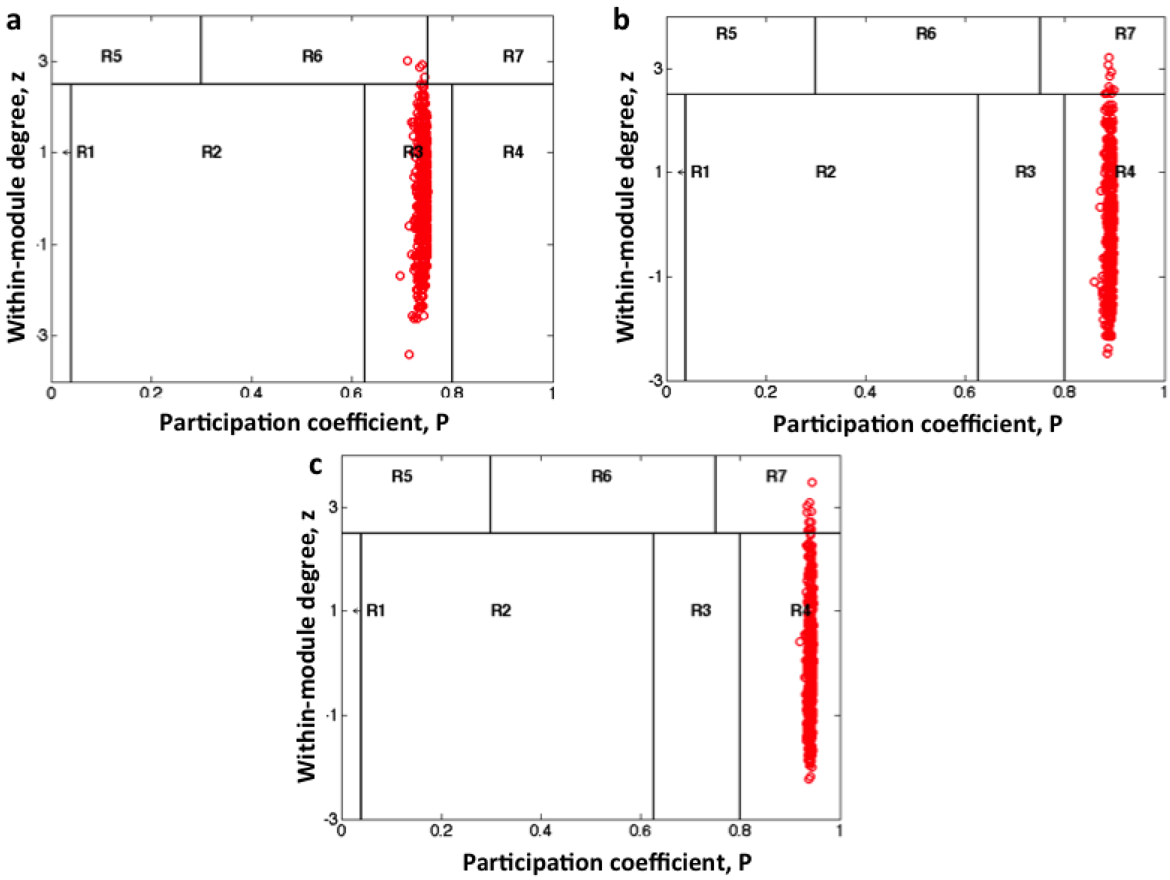

Figure 3. Random graphs in Guimerà-Amaral cartography. In the plots the cartography described in[2,3] for a random graph generated by the Erdös-Rènyi model[4] constituted by 1000 nodes and 50.000 edges are shown. The partitioning of the network changes from panels **a** to **c**: 4, 10, 20 clusters, respectively. Each plot shows the achievement of the maximum values of the participation coefficient P given by equation 3 in the main text that relies on the network partitioning: $P_{MAX} = 1 - \frac{1}{4} = 0.75$ (panel **a**); $P_{MAX} = 1 - \frac{1}{10} = 0.9$ (panel **b**); $P_{MAX} = 1 - \frac{1}{20} = 0.95$ (panel **c**).

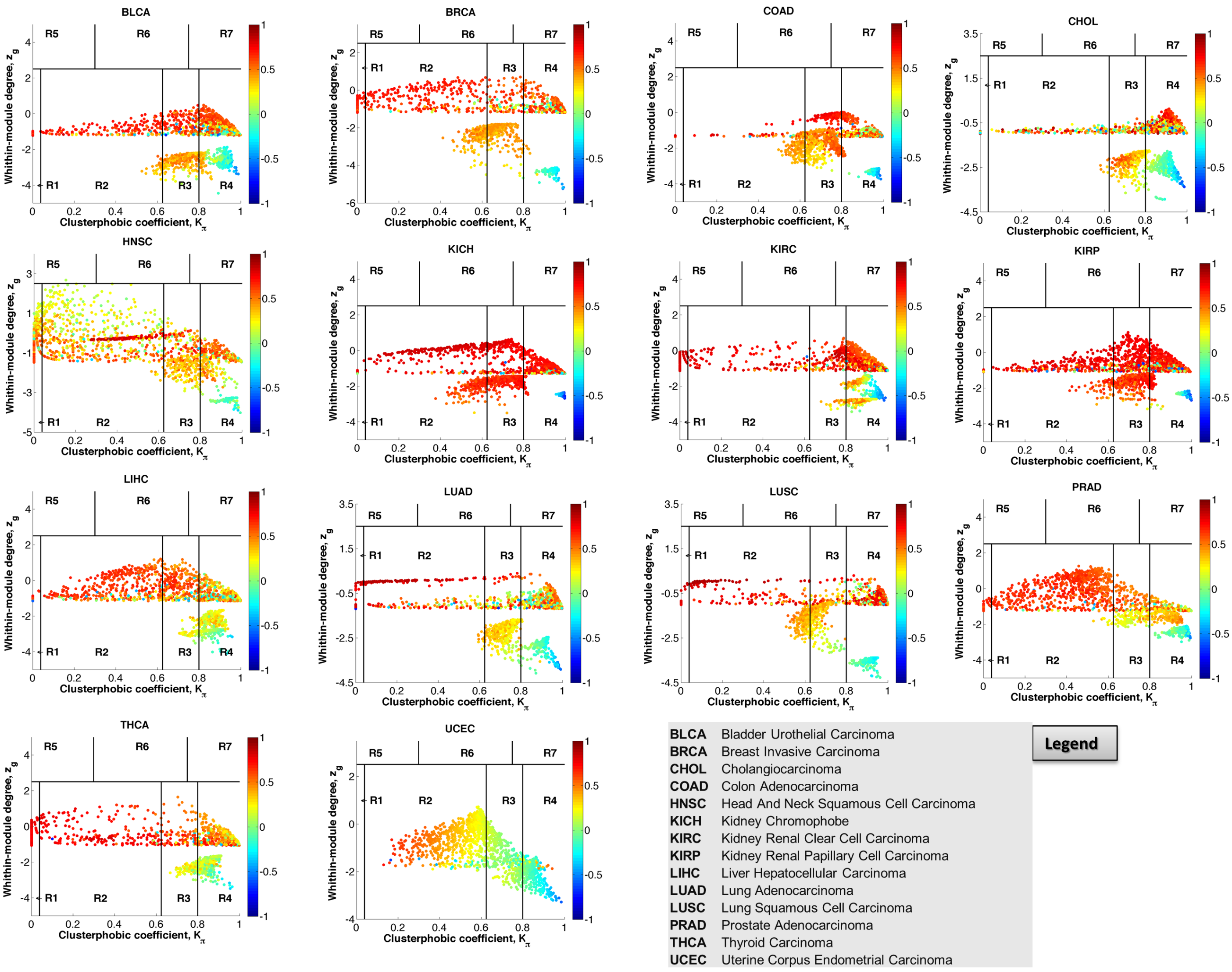

Figure 4. Heat cartography map for 14 human cancers of TCGA. In each panel the cartographic representation of the gene expression network - obtained using the RNA-sequencing data of expression available on TCGA data portal - is represented for each tumor we studied (see legend). The x and y axes correspond to the within-module degree z_g and the clusterphobic coefficient K_π, respectively. z_g is a normalised measure of intramodule communications of each node, while K_π represents the mode of communication between nodes in different modules. Each point represents a node in the network coloured according to its APCC value. The plane identified by z_g, K_π is divided into seven regions each defining a specific node role. Switch genes correspond to nodes coloured in blue and falling in R4 region.

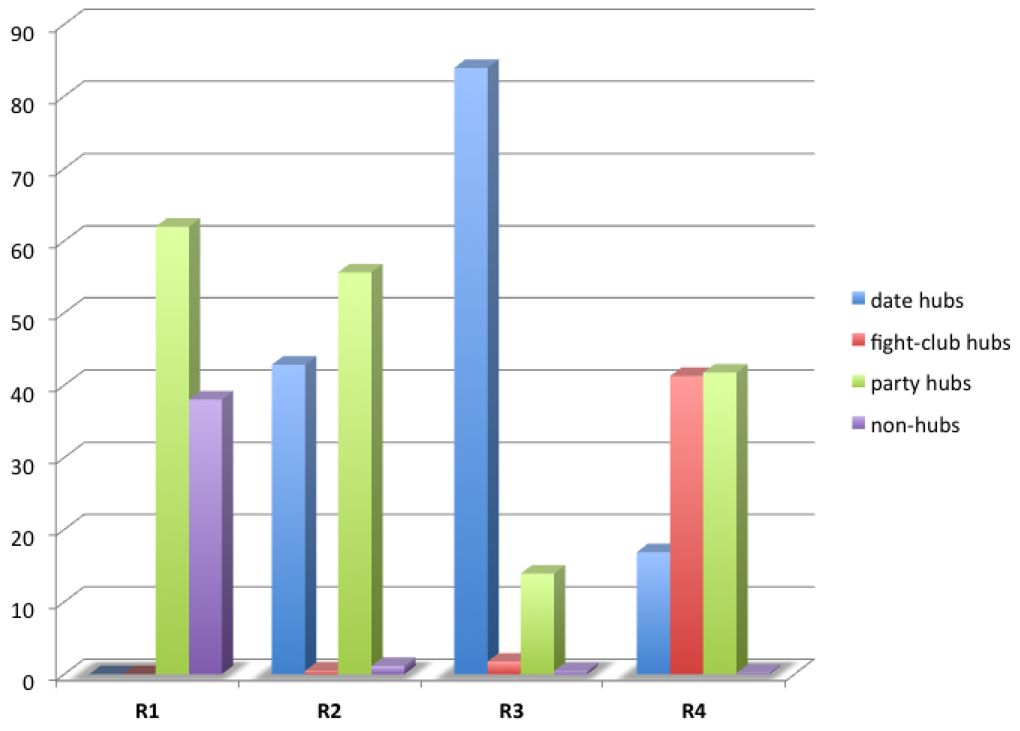

Figure 5. Correlation between structural node roles and gene expression in breast invasive carcinoma. Plot shows the correlation between hub types and node roles in the correlation network of breast invasive carcinoma downloadable from TCGA. Hubs are classified in party, date and fight-club according to the average expression correlation with their partners. The rectangles represent the percentage of each hub type over all nodes in a given role. Note that 'hub' as used in the hub type names refers only to within-community hubs, but 'non-hub' nodes could be also hubs in the global network if they have high degree (> 5).

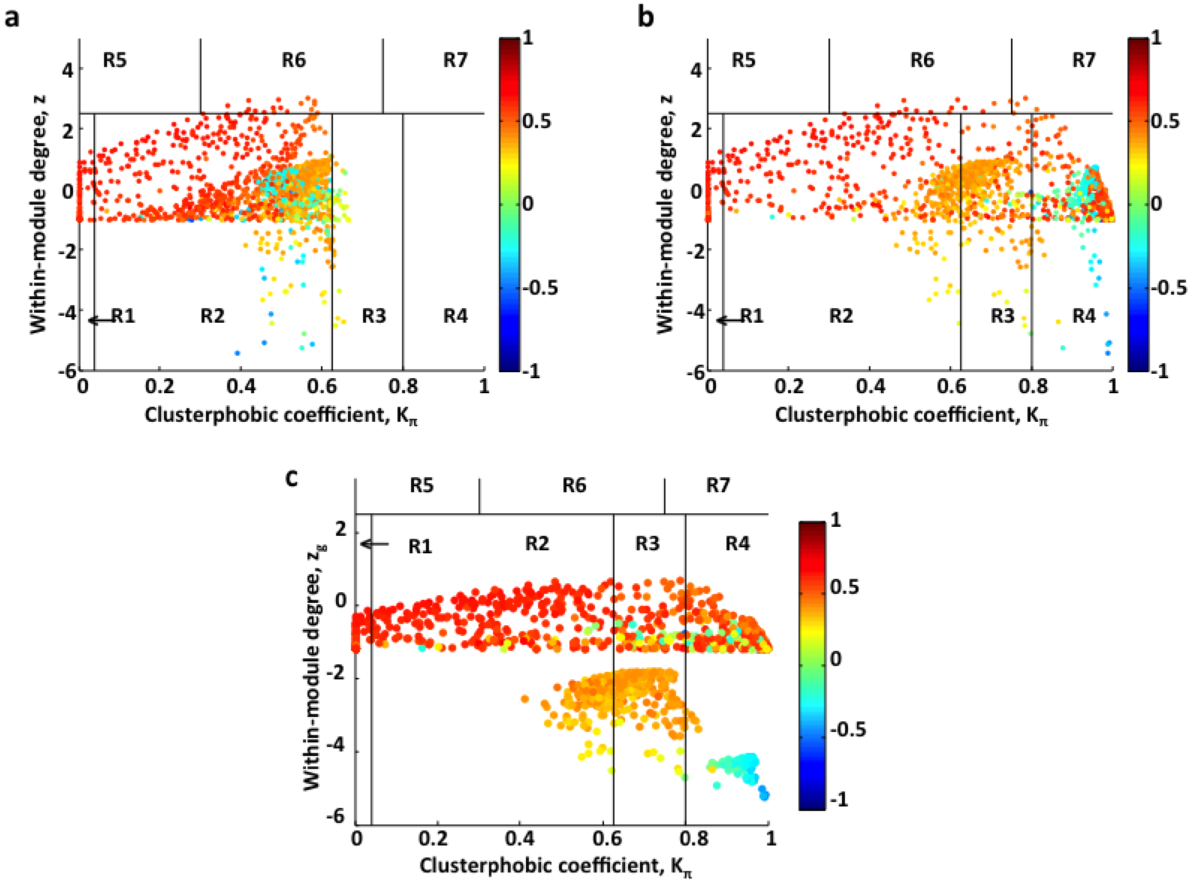

Figure 6. Heat cartography map for breast invasive carcinoma. The figure shows the heat cartographies of the breast invasive carcinoma from TCGA calculate by using: Guimerà-Amaral approach (panel **a**); a mixed approach where the y-axis represents the within-module degree z defined in[2,3] and the x-axis corresponds to our definition of the clusterphobic coefficient K_π (panel **b**); our approach (panel **c**). Switch genes correspond to nodes coloured in blue and falling in R4 region. The plots show that both the new statistics are need for their blatant identification.

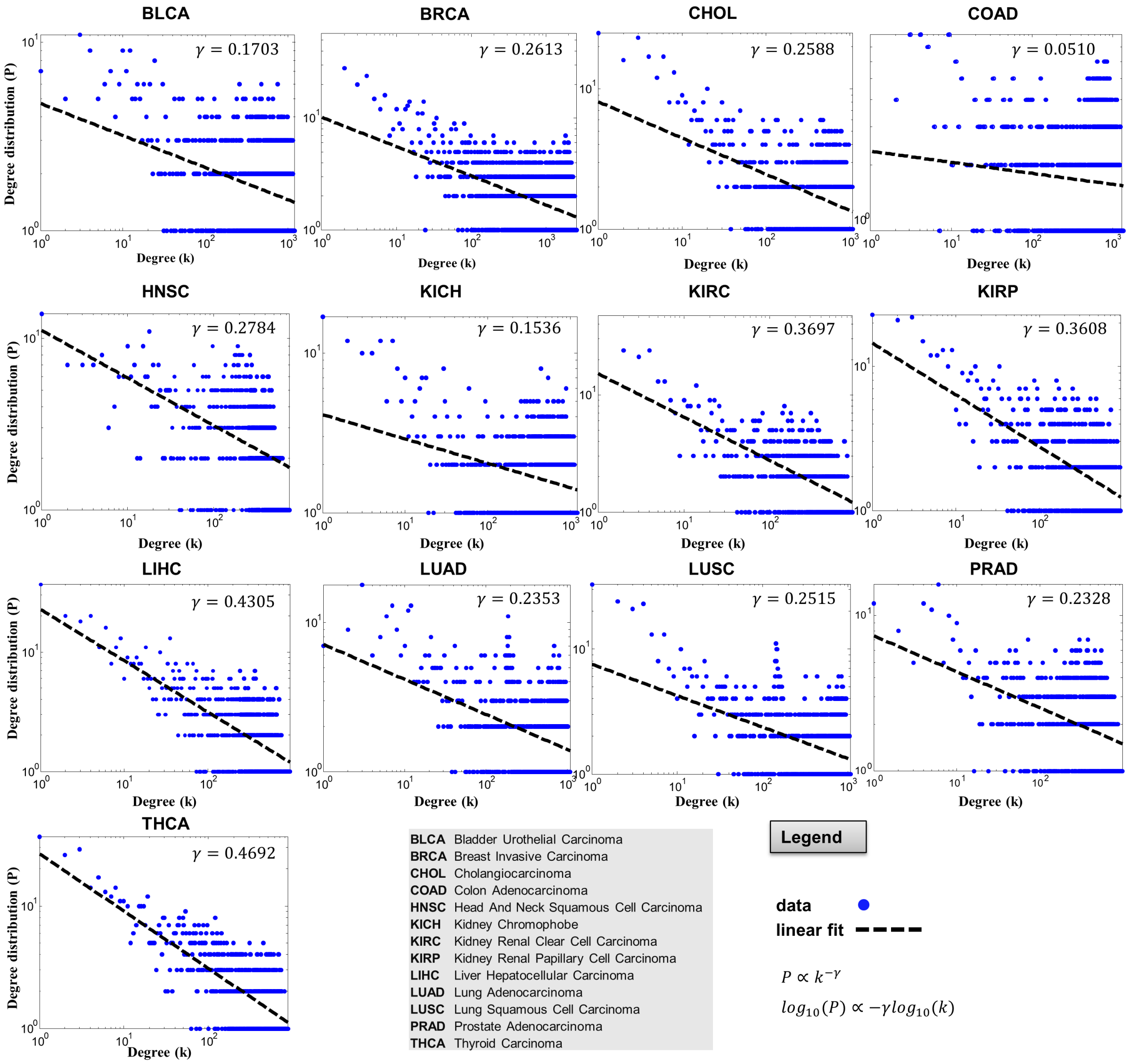

Figure 7. Degree distribution of the gene expression networks for 14 human cancers of TCGA. The figure shows the degree distribution for each gene expression network we studied. In all cases the degree distribution follows a power law behavior (*i.e.* $P(k) \propto k^{-\gamma}$, where k is the degree and γ is the power law exponent that is $0 < \gamma < 1$). In all panels, blue dots represent the logarithm of the degree (x-axis) plotted against the logarithm of the number of nodes with such degree (y-axis); the black dashed line is a linear fit of such data (*i.e.* $\log_{10} P \propto -\gamma \log_{10} k$) based on the least squares method.

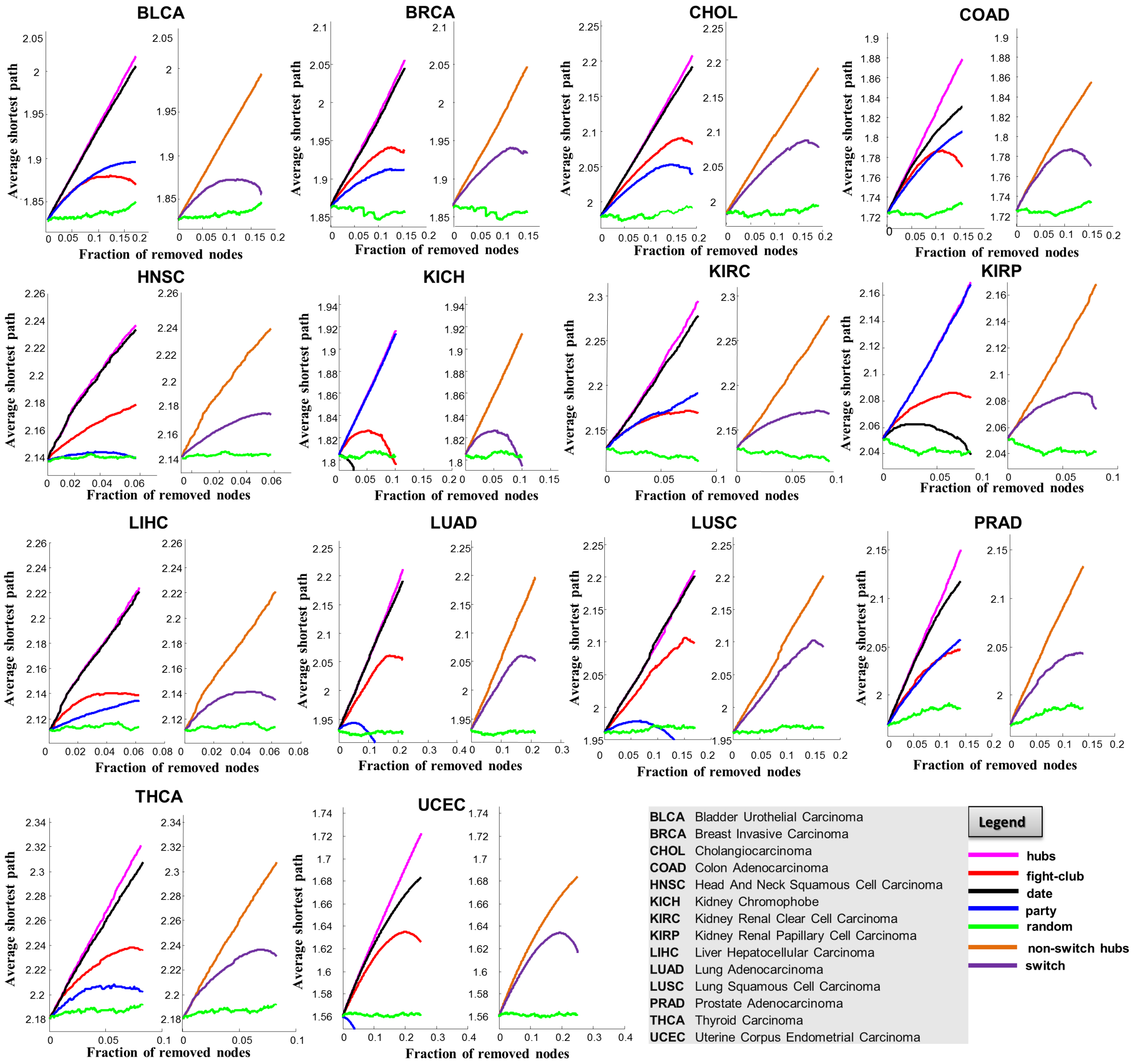

Figure 8. Effects of hub deletion on network connectivity for 14 human cancers of TCGA. In each panel the average shortest path as function of the removal of selected nodes in the gene expression network - obtained using the RNA-sequencing data of expression available on TCGA data portal - is represented for each tumor we studied (see legend). In order to evaluate the effects of switch genes deletion on the network connectivity we compare their removal with the one of the other hubs and set the number of nodes to be removed equal to the total number T of switch genes. In the left panel of each tumor, the robustness of the network is evaluated by removing in order of decreasing degree: i. the first T hubs (named terrorist attack, pink curve); ii. randomly chosen T nodes (named failure, green curve); iii. the first T party (blue curve), T date (black curve), and T fight-club hubs (red curve). In the right panel of each tumor, the robustness of the network is evaluated by removing in order of decreasing degree: i. the T switch genes sorted by decreasing degree (violet curve); ii. randomly chosen T nodes (named failure, green curve); iii. the first T hubs (not including switch genes) sorted by decreasing degree (yellow curve).

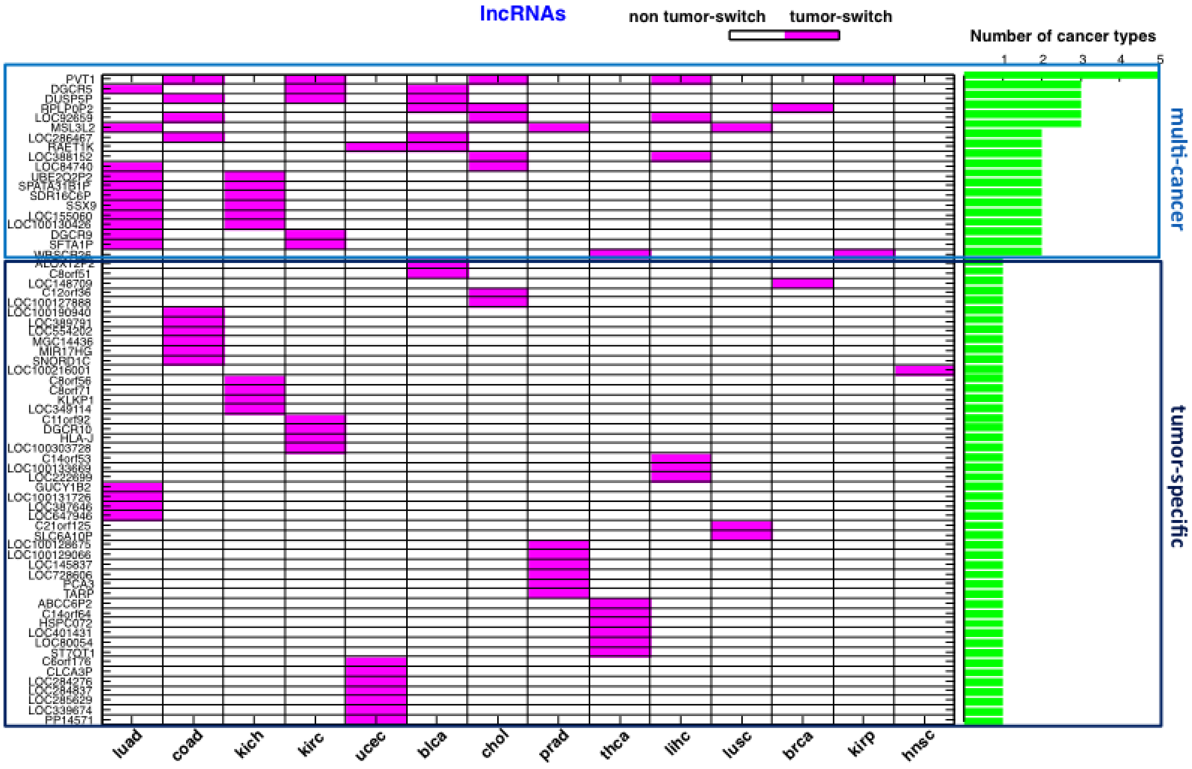

Figure 9. Long non-coding RNAs acting as switch genes across 14 human cancers of TCGA. [Left] The distribution of long non-coding RNA (lncRNAs) acting as switch genes across 14 human cancers of TCGA is rendered in a heat map where violet (white) color indicates whether a given lncRNA listed in the row is (is not) a switch gene for the tumors listed in the columns. Columns are sorted (from left to right) in decreasing order according to the number of lncRNAs identified as switch genes. [Right] In the histogram each green bar represents the number of cancer types where a given lncRNA listed in the corresponding row has been identified as switch gene. The cyan box refers to switch lncRNAs that are shared by more than one cancer type (pan-cancer switch lncRNAs), while dark blue box refers to lncRNAs that has been found in only one tumor (tumor-specific switch lncRNAs). Abbreviations. blad: bladder urothelial carcinoma; brca: breast invasive carcinoma; chol: cholangiocarcinoma; coad: colon adenocarcinoma; hnsc: head and neck squamous cell carcinoma; kich: kidney chromophobe; kirc: kidney renal clear cell carcinoma; kirp: kidney renal papillary cell carcinoma; lihc: liver hepatocellular carcinoma; luad: lung adenocarcinoma; lusc: lung squamous cell carcinoma; prad: prostate adenocarcinoma; thca: thyroid carcinoma; ucec: uterine corpus endometrial carcinoma.

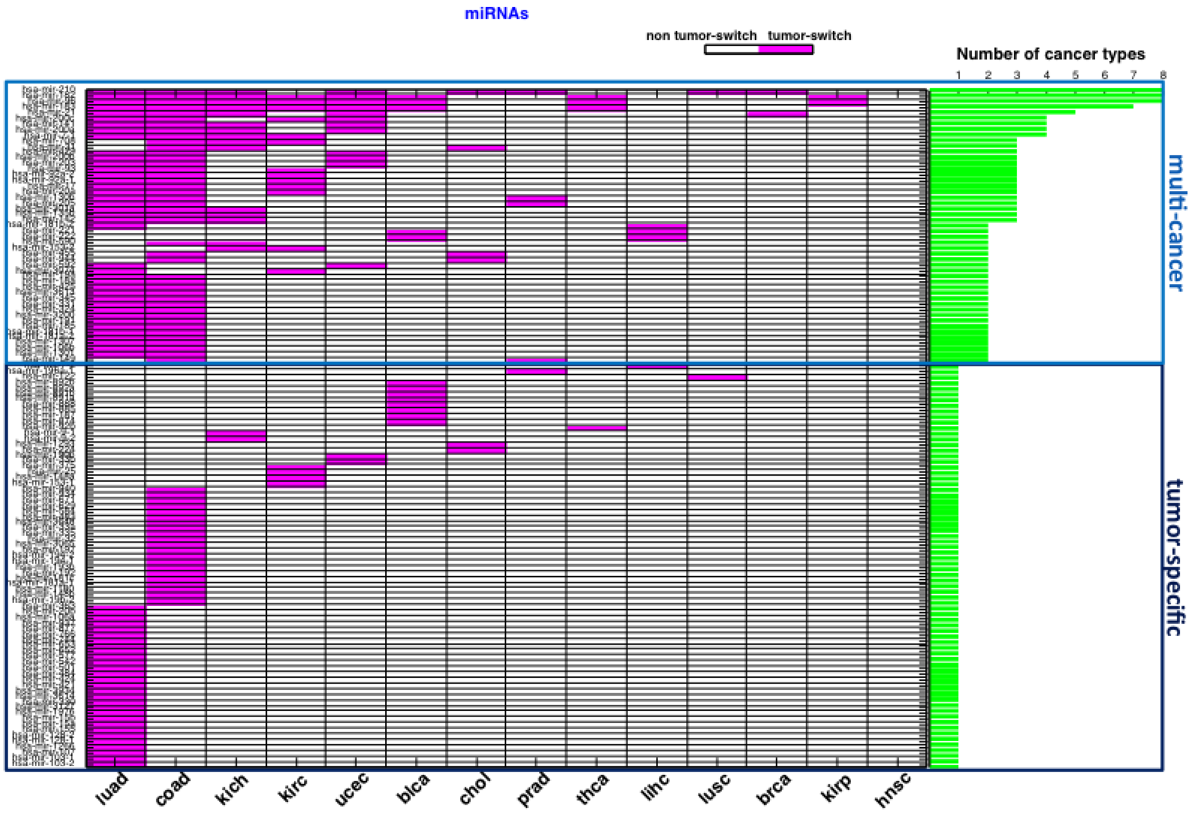

Figure 10. miRNAs acting as switch genes across 14 human cancers of TCGA. [Left] The distribution of miRNAs acting as switch genes across 14 human cancers of TCGA is rendered in a heat map where violet (white) color indicates whether a given miRNA listed in the row is (is not) a switch gene for the tumors listed in the columns. Columns are sorted (from left to right) in decreasing order according to the number of miRNAs identified as switch genes. [Right] In the histogram each green bar represents the number of cancer types where a given miRNA listed in the corresponding row has been identified as switch gene. The cyan box refers to switch miRNAs that are shared by more than one cancer type (pan-cancer switch miRNAs), while dark blue box refers to miRNAs that has been found in only one tumor (tumor-specific switch miRNAs). Abbreviations. blad: bladder urothelial carcinoma; brca: breast invasive carcinoma; chol: cholangiocarcinoma; coad: colon adenocarcinoma; hnsc: head and neck squamous cell carcinoma; kich: kidney chromophobe; kirc: kidney renal clear cell carcinoma; kirp: kidney renal papillary cell carcinoma; lihc: liver hepatocellular carcinoma; luad: lung adenocarcinoma; lusc: lung squamous cell carcinoma; prad: prostate adenocarcinoma; thca: thyroid carcinoma; ucec: uterine corpus endometrial carcinoma.

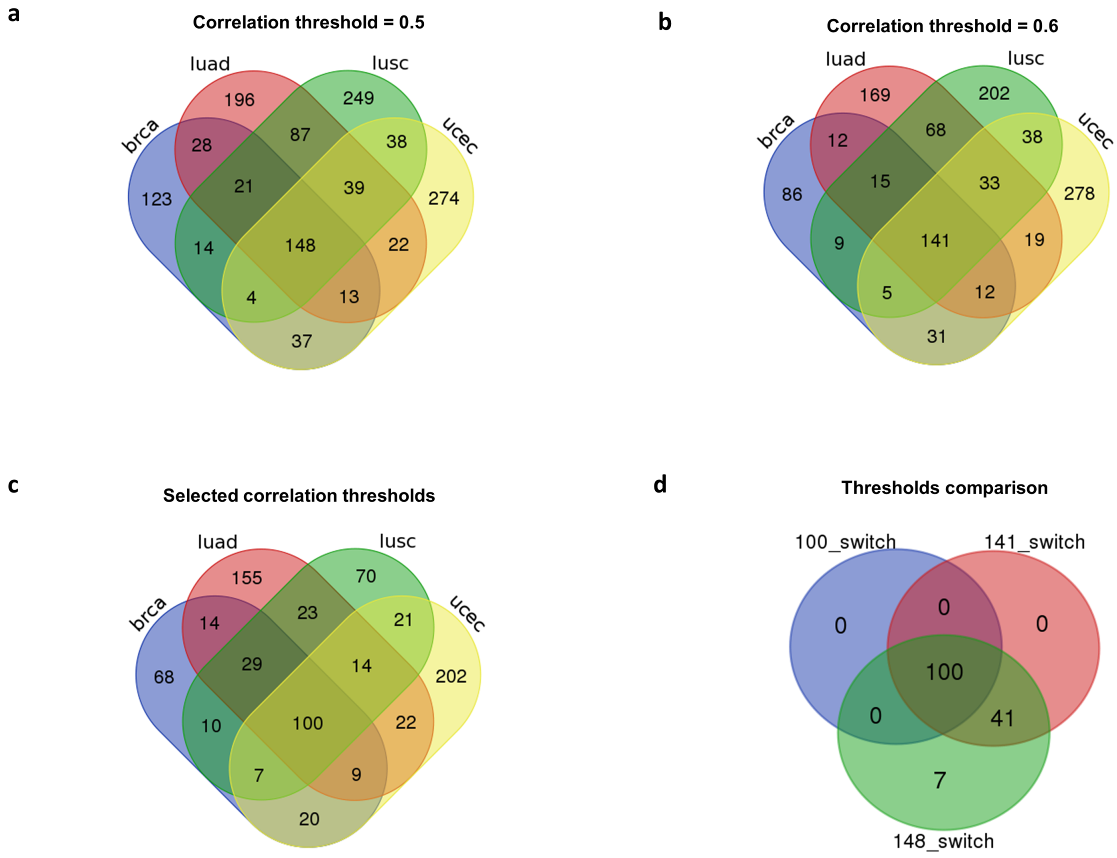

Figure 11. Cancer-recurrent switch genes by varying the Pearson correlation threshold. By applying SWIM on the large panel of TCGA cancer datasets[5] we consistently identified cancer-related switch genes that are later compared in order to group cancer types based on their degree of switch genes similarity (see Figure 3a - Right in the main text). Thus, it became possible to point out a cluster of four cancer datasets (*i.e.* breast invasive carcinoma, lung adenocarcinoma, lung squamous cell carcinoma, and uterine corpus endometrial carcinoma) sharing a substantial number of switch genes (N=100), whose biological relevance is discussed in the main text. In order to show that this finding is not related to the threshold on the Pearson correlation coefficient selected to build each cancer-specific correlation network, we focused on the cluster of the four cancer datasets and for each of these datasets we run SWIM for different values of Pearson correlation threshold, equal to 0.5 and 0.6. Then, we drew the Venn diagrams detailing the counts of cancer-specific or common switch genes among the four grouped cancers for the Pearson correlation threshold equal to 0.5 (panel **a**) and 0.6 (panel **b**). Panel **c** shows the same Venn diagram as in Figure 3b of the main text, where the counts of cancer-specific or common switch genes among the four grouped cancers correspond to the list of switch genes obtained by considering cancer-specific thresholds, *i.e.* 0.62 for breast invasive carcinoma, 0.69 for lung adenocarcinoma, 0.75 for lung squamous cell carcinoma, 0.74 for uterine corpus endometrial carcinoma (see Supplementary Table 1). In panels **a**, **b** and **c** we obtained a list of 148, 141 and 100 cancer-recurrent switch genes, respectively. In panel **d**, the Venn diagram shows the intersection among these three lists of cancer-recurrent switch genes. Of note, the list of 100 cancer-recurrent switch genes, which is obtained with the cancer-specific Pearson correlation threshold and discussed in the paper, is included within the lists of cancer-recurrent switch genes obtained with Pearson correlation thresholds of 0.5 and 0.6. This result proves that our findings are independent from the thresholds chosen to build the correlation networks. Abbreviations. brca: breast invasive carcinoma; luad: lung adenocarcinoma; lusc: lung squamous cell carcinoma; ucec: uterine corpus endometrial carcinoma.

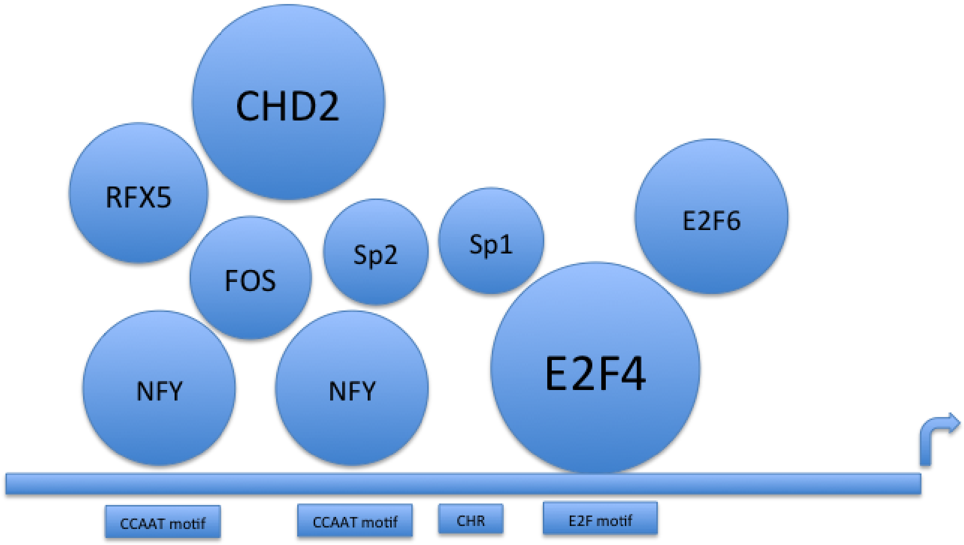

Figure 12. Putative common regulatory module of the promoters of the 100 cancer-recurrent switch genes. The promoter regions of the prevalent set (N=100, see Supplementary Table 4) of switch genes identified as recurrent across multiple tumors (namely, breast invasive carcinoma, lung adenocarcinoma, lung squamous cell carcinoma and uterine corpus endometrial carcinoma) were analyzed by using PscanChip[6] in the K562 cell line, in order to investigate an enrichment in the double-CCAAT box module. This module resulted to be contained in about half of the promoters. Moreover, a significant enrichment for E2F motifs were found mainly outside the NF-Y bound regions (*i.e.* the double-CCAAT box module) rather than within them, suggesting no overlap in binding between the two transcription factors. Circles represent transcription factors, with a size proportional to the number of switch genes they are found in (100% for E2F4). Factor E2F4, with E2F6, can be considered a regulator of virtually every cancer-recurrent switch gene, with the NF-Y module containing FOS, RFX5, SP1/SP2 and CHD2 covers at least half of them.

References

1. Han, J.-D. J. *et al.* Evidence for dynamically organized modularity in the yeast protein-protein interaction network. *Nature* **430**, 88–93 (2004). DOI 10.1038/nature02555.

2. Guimerà, R. & Amaral, L. A. N. Cartography of complex networks: modules and universal roles. *J Stat Mech* **P02001**, P020001–1–P02001–13 (2005).

3. Guimerà, R. & Amaral, L. A. N. Functional cartography of complex metabolic networks. *Nature* **433**, 895–900 (2005).

4. Bollobás, B. Random graphs. *Cambridge University Press* **2end edn** (2001).

5. Cancer Genome Atlas Research Network *et al.* The cancer genome atlas pan-cancer analysis project. *Nat Genet* **45**, 1113–20 (2013). DOI 10.1038/ng.2764.

6. Zambelli, F., Pesole, G. & Pavesi, G. Pscanchip: finding over-represented transcription factor-binding site motifs and their correlations in sequences from chip-seq experiments. *Nucleic acids research* **41**, W535–W543 (2013).
